# Structural Engineering of Layered Nanomaterials for Biomedical Applications

**DOI:** 10.1021/acs.chemrev.5c00193

**Published:** 2025-07-28

**Authors:** Tingting Hu, Yu Yang, Tao Wang, Min Ge, Chaojie Yu, Jiajia Zha, Xiangrong Pan, Zhan Zhou, Lufang Ma, Ruizheng Liang, Chaoliang Tan

**Affiliations:** † Department of Electrical Engineering, 53025 City University of Hong Kong, 83 Tat Chee Ave, Kowloon Tong, Hong Kong SAR 999077, P. R. China; ‡ State Key Laboratory of Chemical Resource Engineering, Beijing Advanced Innovation Center for Soft Matter Science and Engineering, 47832 Beijing University of Chemical Technology, Beijing 100029, P. R. China; § College of Chemistry and Chemical Engineering, Henan Key Laboratory of Function-Oriented Porous Materials, 58292 Luoyang Normal University, Luoyang 471934, P. R. China; ∥ Quzhou Institute for Innovation in Resource Chemical Engineering, Quzhou 324000, P. R. China; ⊥ Department Biomedical Engineering, City University of Hong Kong, 83 Tat Chee Ave, Kowloon Tong, Hong Kong SAR 999077, P. R. China; # Hong Kong Branch of National Precious Metals Material Engineering Research Center (NPMM), City University of Hong Kong, 83 Tat Chee Ave, Kowloon Tong, Hong Kong SAR 999077, P. R. China

## Abstract

Layered nanomaterials have been recognized as promising nanomaterials for biomedical applications due to their tunable crystal phase, easy exfoliation, capability as the host to be intercalated with guest species, and layer-dependent electronic/optoelectronic properties. Recent advances in structural engineering strategies enable manipulating layered nanomaterials at the atomic level, activating and/or optimizing their properties, and overcoming existing limitations for unlocking unprecedented performance in biomedical applications. In this Review, we comprehensively summarize the latest advancements in structural engineering of layered nanomaterials, focusing on their applications in the biomedical field. First, layered nanomaterials explored in the biomedical field enabled by structural engineering are presented based on their composition and structures, followed by highlighting their unique advantages for structural engineering at the atomic level. Then, the structural engineering strategies of layered nanomaterials including crystal phase engineering, defect engineering, heteroatom doping, interlayer engineering, and crystalline-to-amorphous phase engineering are comprehensively discussed, alongside insights on the advanced characterization techniques. Moreover, the transformative potential of structural engineering to optimize the performance of layered nanomaterials for diverse biomedical applications is discussed in depth. Finally, this Review is concluded with perspectives on the key challenges and bottlenecks of structural engineering of layered nanomaterials in the biomedical field, providing potential solutions and outlining future directions.

## Graphical abstract

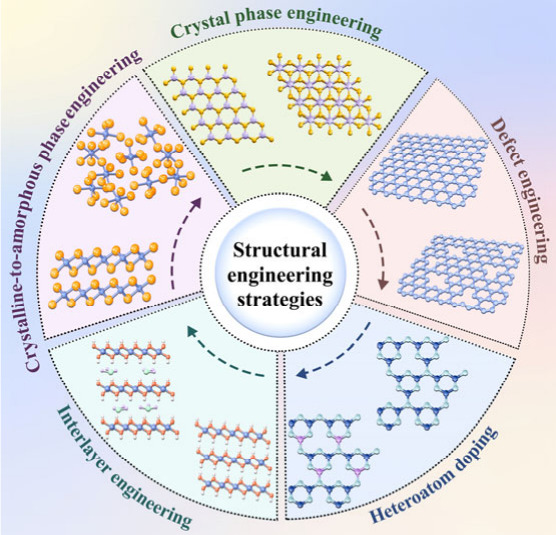

## 1 Introduction

Biomedicine stands as a pivotal subject that is intrinsically linked to human health and well-being.^1^ In recent years, the field of biomedical technology has witnessed remarkable advancements, offering vast potential for applications in medical diagnosis, therapeutic interventions, and health management.^2–4^ These innovations promise to provide more convenient, efficient and superior healthcare services, thereby enhancing the overall quality of medical care. The tremendous advances in fields such as gene research, biomedical engineering, and life sciences have further underscored the critical importance and urgency of this field.^5–8^ Amidst a growing global emphasis on public health, the healthcare industry is undergoing transformative changes. In this context, materials science has emerged as a crucial enabler for diverse biomedical applications, leveraging their unique properties such as drug delivery capacities, therapeutic efficacy, antibacterial activity, and/or tissue regeneration potential.^9–13^ Considerable progress has been achieved in the design of various types of materials, ranging from traditional materials to cutting-edge nanomaterials and advanced polymers, all of which are finding direct applications in addressing complex biomedical challenges.^14–17^

Especially, with the swift advancements in nanotechnology, nanomaterials such as inorganic nanomaterials, organic nanomaterials, and hybrid organic–inorganic nanomaterials have become cutting-edge scaffolds for designing bioactive materials due to their exceptional physicochemical properties (e.g., high surface-area-to-volume ratio, quantum effect, small size effect, surface effect) arising from their nanoscale sizes, providing a new horizon for biomedicine.^18–22^ In particular, inorganic nanomaterials possess unique material- and size-dependent physicochemical properties and versatile morphologies/sizes, characteristics that organic nanomaterials lack, showing promising potential in the biomedical field.^19,23^ Among them, layered nanomaterials featured with unique layered structure, such as graphene and its derivatives,^24,25^ layered double hydroxides (LDHs),^26,27^ transition metal dichalcogenides (TMDs) (e.g., MoS_2_, WS_2_, MoSe_2_, WSe_2_, TiS_2_, and TaS_2_),^28–30^ layered metal oxides (MoO_3_ and WO_3_),^31,32^ graphitic carbon nitride (g-C_3_N_4_),^33,34^ and metal carbides and nitrides (MXenes),^35,36^ have been recognized as promising nanomaterials for biomedical applications by virtue of their tunable crystal phase, easy exfoliation, capability as the host to be intercalated with various guest species (e.g., anions, cations, conductive polymers, and organic molecules), and layer-dependent electronic/optoelectronic properties, providing unprecedented opportunities in drug delivery, bioimaging, cancer therapy, theranostics, biosensing, and antibacterial application.^37–41^

Alongside the pursuit of new layered nanomaterials and the investigation of their unique characteristics, significant efforts are directed toward precisely controlling and tailoring their properties in a predictable manner. A major focus lies in structurally modifying these layered nanomaterials or engineering them into specific structures at the atomic level to meet higher requirements for biomedical applications, known as structural engineering at the atomic level. In the current age of nanotechnology and nanoscience, structural engineering plays a critical role in tailoring the properties of individual material components and optimizing performance toward functional systems.^42–46^ Especially, layered nanomaterials have great advantages in structural engineering at the atomic level, as they can be intercalated with various kinds of guest species, e.g., ions and molecules, to regulate their crystal phase, interlayer spacing, and electronic structure, or rich defects can be generated on them through etching and doping.

Recent progress in structural engineering strategies has shown significant potential for precisely engineering layered nanomaterials at the atomic level, activating and/or optimizing material properties, and overcoming current material limitations by tuning their crystal phase, oxygen vacancies (OVs), interlayer distances and/or crystallinity as well as heteroatom doping, showing great promise for activating or optimizing consequent performance to a new level in biomedical applications.^47–51^ For example, the introduction of OVs into ultrathin BiO_2*–x*_ nanosheets using sonication-assisted liquid-phase exfoliation significantly promoted the separation of ultrasound (US)-irradiated electrons and holes, with electrons being captured by H_2_O_2_ and O_2_ to augment hydroxyl radicals (·OH) and superoxide anion (·O_2_^–^) generation based on peroxidase and oxidase-like activities.^52^ The aqueous intercalation of layered MoO_3_ nanobelts not only expanded interlayer spacing but also introduced rich defects and facilitated partial reduction of Mo^6+^ to Mo^5+^, thus activating the MoO_3_ nanobelts as an efficient nanozyme for high-efficiency photothermally enhanced catalytic therapy.^53^ There are a wide range of structural engineering strategies have been developed and they can be classified based on how they modify the structure of layered nanomaterials: crystal phase engineering, defect engineering, heteroatom doping, interlayer engineering, and crystalline-to-amorphous phase engineering.^45–48,54,55^ Grasping the fundamental mechanisms behind these strategies is essential for advancing structural design and property optimization, which is also crucial for expanding the biomedical applications of layered nanomaterials.

Over the past decade, our group has contributed significantly to this field. For instance, we pioneered the development of defect-rich CoMo-LDH nanosheet, activated through acid etching-mediated defect engineering, as a highly effective inorganic photosensitizer (PS) for photodynamic therapy (PDT) in the second near-infrared (NIR-II) window (1000–1870 nm).^56^ Most recently, we proposed a crystalline-to-amorphous phase transformation strategy to engineer CoW-LDH and CoCuMo-LDH nanosheets as highly active sensitizers for sonodynamic therapy (SDT) and NIR-II PDT, respectively.^57,58^ We have also reported the structural engineering like crystal phase engineering, defect engineering and interlayer engineering of layered TMDs (e.g., MoS_2_) and layered metal oxides (MoO_3_) for high-performance photothermal therapy (PTT), catalytic therapy and SDT to against cancer or bacteria.^53,59–64^ Considering the remarkable progress made in the field, we believe that providing a timely and comprehensive review on this emerging topic is crucial for advancing this field. Nevertheless, to the best of our knowledge, several Review papers on the structural engineering of two-dimensional (2D) nanomaterials focus exclusively on specific applications, such as energy storage, catalysis, electronics/optoelectronics, and sensing. While there are some published Review papers on the biomedical applications of modified layered nanomaterials, these often fall short in thoroughly summarizing and discussing the structural engineering strategies involved.

In this Review, we aim to give a comprehensive, authoritative and critical Review article to summarize the latest advancements in structural engineering of layered nanomaterials for biomedical applications (Figure 1). First, the layered nanomaterials that have been explored in the biomedical field enabled by structural engineering are presented based on their composition, structure and properties. Then, the unique advantages of layered nanomaterials for structural engineering at the atomic level are highlighted. Subsequently, the structural engineering strategies of layered nanomaterials including crystal phase engineering, defect engineering, heteroatom doping, interlayer engineering, and crystalline-to-amorphous phase engineering are comprehensively discussed. Moreover, the well-developed techniques, such as powder X-ray diffraction (XRD), transmission electron microscopy (TEM)/scanning transmission electron microscopy (STEM), X-ray photoelectron spectroscopy (XPS), electron spin resonance spectroscopy (ESR), atomic force microscopy (AFM), X-ray absorption fine structure spectroscopy (XAFS), Raman spectroscopy, and nuclear magnetic resonance (NMR) spectroscopy for characterizing the structural characteristics of these layered nanomaterials induced by structural engineering are systematically summarized. Furthermore, the great potential of structural engineering strategies to optimize the performance of layered nanomaterials for diverse biomedical applications (e.g., drug delivery, bioimaging, cancer therapy, theranostics, biosensing, and antibacterial application) are discussed in depth. Finally, this Review is concluded with perspectives on the key challenges and bottlenecks of structural engineering of layered nanomaterials in the biomedical field, providing potential solutions and proposing future perspectives.

**Figure 1:**
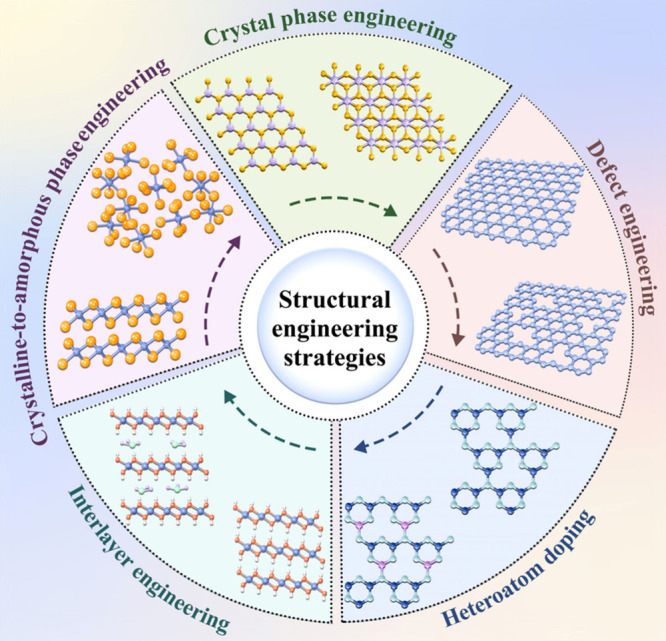
Overview of structural engineering strategies for layered nanomaterials, including crystal phase engineering, defect engineering, heteroatom doping, interlayer engineering, and crystalline-to-amorphous phase engineering.

## 2 Composition, Structure, and Properties of Layered Nanomaterials

So far, large quantities of layered nanomaterials with varying compositions and crystal structures have been prepared through various synthesis methods. In general, the commonality of layered nanomaterials is that in-plane atoms are strongly connected to each other by chemical bonds in each layer, and these layers are weakly stacked together by van der Waals (vdW) interactions, forming bulk crystals with a unique layered structure.^65,66^ As a typical example, graphite is a star-level layered carbon material and graphene, a single atomic thick carbon film, can be obtained by simple mechanical exfoliation of graphite by scotch tape.^67^ Besides, other layered nanomaterials with enormous application potential in the biomedical field have grown rapidly, including LDHs, TMDs, layered metal oxides, g-C_3_N_4_, and MXenes. Their layered nature enables them to be exfoliated into ultrathin 2D nanosheets through top-down methods, e.g., micromechanical cleavage, mechanical force-assisted liquid exfoliation, and ion intercalation-assisted liquid exfoliation.^66^ In addition, layered nanomaterials can form different crystal phases depending on atomic arrangement, coordination modes, and/or the stacking order between layers.^68,69^ These crystal phases determine the properties and functions of nanomaterials.^70–72^ In this section, we mainly provide a detailed introduction to the crystal structures and properties of these extensively studied layered nanomaterials on the basis of their composition (Figure 2).

**Figure 2:**
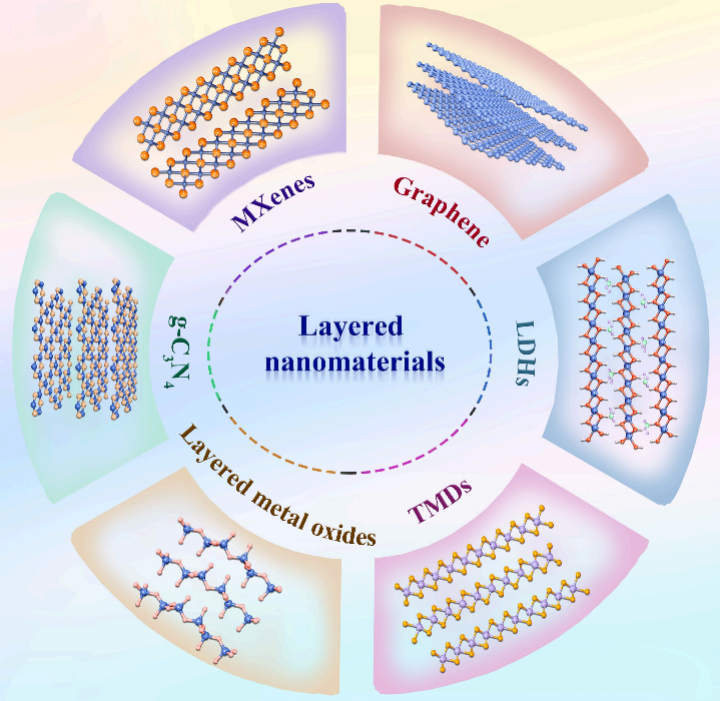
Schematic illustration of layered nanomaterials including graphene, LDHs, TMDs, layered metal oxides, g-C_3_N_4_, and MXenes.

### 2.1 Graphene and Its Derivatives

Graphene, an allotrope of carbon in 2D structure form,^24^ is constituted by sp^2^-bonded carbon atoms arranged in a hexagonal-close-packed honeycomb lattice, where each carbon atom is covalently bound to three adjacent ones through σ-bond at a distance of 1.42 Å.^73–75^ Individual layers are stacked and connected by vdW force to form the three-dimensional (3D) bulk graphite with a distance of 3.35 Å between adjacent layers.^74^ The interesting graphene derivatives, such as graphene oxide (GO) and reduced graphene oxide (rGO), have also garnered significant research attention.^76,77^ GO is an oxygen-containing functional group derivative with hydroxyl, carboxyl, and epoxy functional groups distributed on the basal plane and its edges, presenting a nonaromatic surface of sp^3^ carbon atoms with an isolated small aromatic sp^2^ domains. The strong hydrogen bonding interaction between the epoxy oxygen of GO and water molecules maintains its stacked structure.^78–80^ rGO prepared by the removal of the oxygenated groups of GO possesses medium oxygen-containing functional groups between graphene and GO and has more structural defects than graphene and GO.^81–83^ In addition, graphene possesses the remarkable ability to undergo controlled curvature, enabling its transformation into carbon nanotubes.^84^ This transition from a 2D sheet to a one-dimensional (1D) tubular nanostructure endows carbon nanomaterials with exceptional properties (e.g., high aspect ratio and tunable surface chemistry), which are highly advantageous for nanomedical applications such as targeted drug delivery. Collectively, the aforementioned naked graphene materials, characterized by their large surface area, efficient charge-transfer capability, high elasticity, significant laser absorption rate, curving capability, and ferromagnetic properties, have gained wide popularity in biomedical applications.^76,85^

### 2.2 Layered Double Hydroxides

LDHs, described as [M^2+^_1–*x*_M^3+^_*x*_(OH)_2_]^*x*+^[A^*n*‑^]_*x*/*n*_·*m*H_2_O, are a type of layered materials consisting of positively charged host layers and intercalated guest molecules.^86–88^ In general, M^2+^ and M^3+^ stand for divalent (e.g., Zn^2+^, Mg^2+^, Cu^2+^, Co^2+^, Ni^2+^) and trivalent metal cations (e.g., Al^3+^, Mn^3+^, Fe^3+^, Ga^3+^, In^3+^), respectively, occupying octahedral pores in the brucite-like layer. A^*n*‑^ represents guest molecules located in LDHs interlayer, such as CO_3_^2–^, NO_3_^–^, and Cl^–^. The value of *x* determined by the molar ratio of M^3+^/(M^2+^+M^3+^) is normally within the range of 0.2–0.33, in which case LDHs with high crystallinity and purity can be obtained.^89–95^ The diversity of metal cations and interlayer guest molecules, together with the value of *x*, endow LDHs with a large array of isostructural materials that show great potential for biomedical applications by virtue of their high biocompatibility, tunable chemical composition, pH-responsive biodegradability, and exceptional intercalation properties.^38,96–99^

### 2.3 Transition Metal Dichalcogenides

TMDs are a type of layered materials with a common formula of MX_2_, where X stands for a chalcogen (e.g., Te, Se, S) and M represents a transition metal element (e.g., W, Nb, Mo).^100–102^ TMDs possess a sandwich structure with a transition metal layer positioned between two chalcogen layers.^54^ Each layer is stacked vertically by vdW force, while transition metal atoms within the layer form strong covalent bonds.^68^ In particular, a distinctive characteristic of TMDs is their capability to form various crystal polytypes determined by the stacking orders between layers and/or coordination models between chalcogen atoms and transition metal atoms.^103–106^ The composition, morphology and unique planar structure of TMDs impart it a large specific surface area, strong NIR absorbance, thickness-dependent bandgap, remarkable photostability, and good biodegradability, enabling its utilization for various applications in biomedicine.^107–110^

### 2.4 Layered Metal Oxides

Metal oxides with a general formula of MO_3_ (M = Mo, W, Ta) are reported to have a layered structure consisting of transition metals and oxygen atoms.^111,112^ The electrons of transition metals can be strongly polarized by the oxygen atoms, causing a nonlinear and nonuniform charge distribution in layered metal oxides, which leads to electrostatic shielding in the lattices over scales of 1–100 nm, granting layered metal oxides exceptional local surface and interfacial properties.^113,114^ Similar to LDHs, most layered metal oxides have charged host layers, although there are some exceptions, such as V_2_O_5_.^115,116^ The charged layers are interwoven with cations or anions, or their metal oxide sheets alternate with covalent bonding interlayers to form layer structure. The fundamental properties of layered metal oxides are governed by the transition metal cation species and their oxidation state, resulting in a variable bandgap and tunable stability.^117,118^

### 2.5 Graphitic Carbon Nitride

As a graphene analogue, g-C_3_N_4_ has a vdW layered structure composed of N-substituted graphite skeleton formed by the sp^2^ hybridization of carbon and nitrogen atoms.^119–122^ The g-C_3_N_4_ contains two different structural models: 1) s-triazine formed by the periodic arrangement of condensed s-triazine units with the existence of single-carbon vacancies, and 2) tri-s-triazine subunits linked by tertiary amines accompanied by larger periodic vacancies.^123^ Due to the existence of weak vdW bonding between the sheets, a graphite-like structure known as a honeycomb arrangement is formed in g-C_3_N_4_, with strong covalent connections between each layer of atoms.^123–125^ The semiconducting properties, natural photoresponsiveness, and good biocompatibility resulting from the unique composition and structure of g-C_3_N_4_ make it suitable for diverse biomedical applications.^40,126–128^

### 2.6 Metal Carbides and Nitrides

MXenes are a large family of layered transition metal nitrides, carbides, and carbonitrides, typically represented by the formula of M_n+1_X_n_T_*x*_ (n = 1–6), where M represents the transition metal (Ti, Sc, Zr, V, Cr, Hf, Ta, Nb, W, Mo, etc.), X denotes C and/or N atoms, and T_*x*_ stands for surface terminations (e.g., Te, Se, S, O, OH, F, Cl, and/or Br).^35,129–131^ MXenes are usually prepared by selectively etching the A atomic layers (group IIIA to VIA elements like Ge, Ga, Si, and Al) from the MAX phases, where the M layers are close-packed and unit cell is composed of edge-shared [M_6_X] octahedra.^132–134^ The arrangement of M atoms depends on the stoichiometric parameter n, which plays a crucial role in determining the stability of MXenes.^135–139^ Benefiting from the diversity of elemental composition and the presence of terminated functional groups like fluorine, oxygen, and hydroxyl, MXenes uniquely exhibit tunable electronic conductivity, broad optical absorption properties, and superior hydrophilicity, allowing their burgeoning applications in biomedicine.^140–142^

## 3 Advantages of Layered Nanomaterials for Structural Engineering

Owing to their distinctive electronic, chemical and physical properties, layered nanomaterials exhibit unique advantages for structural engineering, especially at the atomic level (Figure 3), in comparison with traditional materials with 3D crystal structures. These can be summarized as follows:

**Figure 3:**
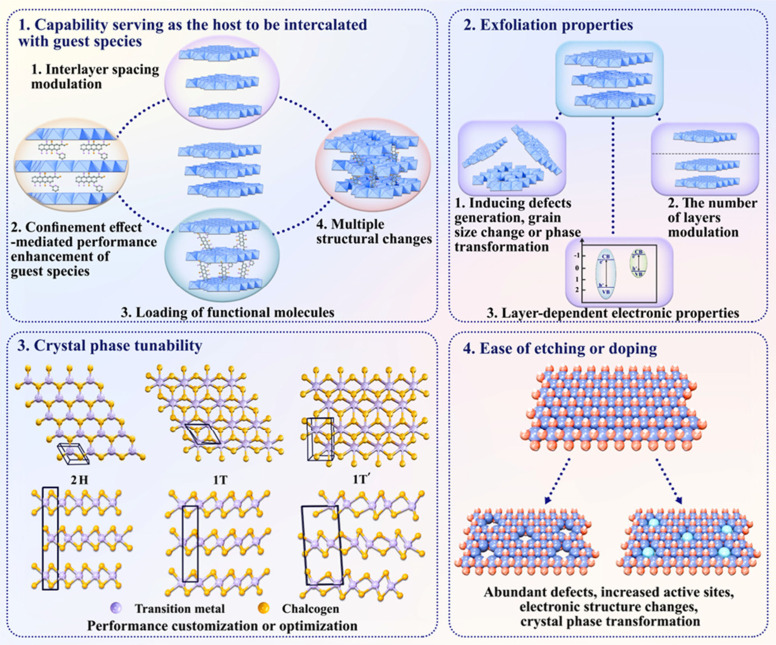
Schematic demonstration of the advantages of layered nanomaterials for structural engineering.

### 3.1 Capability Serving as the Host to Be Intercalated with Various Guest Species

(1) The most unique feature of layered nanomaterials is that they can be intercalated with a variety of guest species due to their unique layered structure, which is not available in 3D materials.^45,143–145^ The type or quantity of intercalated guest species can be altered to modulate the interlayer spacing and/or grain size of layered nanomaterials, potentially tailoring their properties, activating and/or optimizing their performance for specific biomedical applications. For instance, the cointercalation of H_2_O and Na^+^ into layered MoO_3_ nanobelts triggered two structural alterations: the expansion of interlayer spacing and the reduction in length. These changes, coupled with the introduction of abundant defects and partial Mo^6+^→Mo^5+^ reduction, activated the enzyme-mimicking catalytic activity of MoO_3_ nanobelts for tumor catalytic therapy.^53^
(2) In addition to utilizing the intercalation of guest species to alter the properties of layered nanomaterials, layered nanomaterials acting as the hosts can also regulate the properties of guest molecules in turn by virtue of confinement effects (space-confinement effect),^146,147^ thus achieving performance enhancement for biomedical applications. As a typical example, the intercalation of isophthalic acid (IPA) molecules into ZnAl-LDHs yielded a first near-infrared (NIR-I: 750–1000 nm) activated supramolecular PS (IPA/LDH) for efficient two-photon PDT.^148^ Thanks to the space-confinement effects of rigid LDH layers, the intercalated IPA molecules were arranged orderly in LDH interlayer space with prolonged triplet excited lifetime, resulting in efficient singlet oxygen (^1^O_2_) generation under 808 nm laser irradiation with a ^1^O_2_ quantum yield of 0.74. Furthermore, layered nanomaterials have demonstrated significant potential as interlayer reactors for the synthesis of new composite layered nanomaterials. Liu et al. synthesized the ultrathin carbon nitride within the 2D confined interlayer space of MgAl-LDH (CN/LDH) by triggering the interlayer condensation reaction between urea and citric acid (CA).^149^ The obtained CN/LDH possessed an absolute solid-state quantum yield of 95.9 ± 2.2% due to the confinement effect of the host–guest interaction and could function as a fluorescent material for fluorescence imaging. Similarly, Bai et al. demonstrated the synthesis of N-doped amorphous monolayer carbon by polymerizing pyrrole within the confined interlayer space of NiAl-LDHs, demonstrating the versatility of layered nanomaterials as nanoreactors for precise material synthesis.^150^
(3) Besides, the intercalation of ions (cations, anions) or molecules (organic molecules, conductive polymers, therapeutic drugs, imaging contrast agents, etc.) to remain in the interlayer can endow layered nanomaterials with additional functions, thereby broadening the application scope of layered nanomaterials in the biomedical field.^38,83,151,152^ For instance, the intercalation of Nile blue (NB) dye molecules into layered MoO_3_ to prepare NB-intercalated MoO_3*–x*_ (NB-MoO_3*–x*_) nanoparticles not only expanded their interlayer spacing and activated their catalytic activity, but also endowed them with fluorescence imaging capability, achieving fluorescence imaging-guided catalytic therapy.^63^ The introduction of para-aminosalicylic acid (antituberculosis drug) into the interlayer of magnesium layered hydroxide (MgLH) conferred antimycobacterial activity and anti-inflammatory effect, equipping MgLH with the potential to treat tuberculosis.^153^
(4) In addition, the intercalation process usually requires the help of external forces, which may cause the change of valence state, generation of defects, transformation of crystal phase, delamination of layers or doping of heteroatoms. More importantly, the above changes occur simultaneously on the host layered nanomaterials, providing great possibilities to adjust the valence band, electronic structure and crystal phase of layered nanomaterials for various biomedical applications. For instance, the polyaniline (PANI) intercalation successfully induced the transformation of micrometer-long MoO_3_ nanobelts into 2D PANI/MoO_3*–x*_ nanosheets accompanied by the generation of rich OVs and partial reduction of Mo^6+^ to Mo^5+^, thus activating the Fenton-like catalytic activity of MoO_3_ nanobelts for enhanced chemodynamic therapy (CDT).^62^ Similarly, the Li intercalation of bulk MoS_2_ induced synchronous exfoliation and phase transformation, yielding chemically exfoliated MoS_2_ nanosheets with a mixed crystal phase, which engineered MoS_2_ as a NIR photothermal agent for PTT.^154^

### 3.2 Exfoliation Properties

(1) Compared with traditional materials with a 3D crystal structure, layered nanomaterials are more conducive to realizing the precise modulation of the layer numbers and can be delaminated into single-layered or few-layered ones through a variety of strategies.^155–159^ The single-layered or few-layered nanomaterials have a larger specific surface area, which is beneficial for more efficient drug loading and delivery. For example, Gd^3+^-doped MgAl-LDH monolayer nanosheets were demonstrated in coloading of indocyanine green and doxorubicin with an ultrahigh encapsulation efficiency of 99.67% and drug loading content of 797.36%.^160^ Gd^3+^/Yb^3+^ codoped MgAl-LDH nanosheets achieved an ultrahigh loading of a hydrophobic drug (SN38) with a loading content of 925%.^161^ Moreover, compared with the bulk layered nanomaterials, single-layered or few-layered nanomaterials show better dispersion stability and cytocompatibility, facilitating blood circulation and tumor cell uptake.^162^ More importantly, many defects can be introduced, grain size can be controlled, active edges can be increased, or the phase transition can occur during the exfoliation process, enhancing the electrical, optical and biological properties of layered nanomaterials and/or endowing them with new properties, thereby achieving new biomedical applications. For instance, by direct exfoliation of MoS_2_ in pure water, the prepared few-layered MoS_2_ nanosheets exhibited excellent dispersion stability within 3 weeks and layer-dependent cytotoxic modulation effect.^163^ It was found that MoS_2_ nanosheets with 2–5 layers exhibited significantly enhanced bactericidal effects in comparison with the bulk MoS_2_ by inducing membranes mechanical injury and oxidative stress, which may be due to the fact that few-layered MoS_2_ nanosheets could serve as nanoblades to cut the external cell wall of *Salmonella* as its thickness is less than or approximately the same as that of the walls. Recently, the successful transformation of bulk 2H-phase MoS_2_, WS_2_ and MoSe_2_ nanosheets into few-layered 1T-phase TMDs nanosheets through exfoliation method endowed the metallic 1T-phase TMDs with effective antibacterial capacity, which could be attributing to their enhanced chemical oxidation property and electrical conductivity that induced the charge transfer from bacterial membrane to TMDs, causing the continuous disruption of bacteria.^164^ Similarly, the combination of ball milling and Li-intercalation engineered bulk 2H-phase TMD into single-layer 1T-phase TMD nanodots (including MoSe_2_, MoS_2_, MoSSe, Mo_0.5_W_0.5_S_2_, and WS_2_) with high-density exposed active edge sites.^165^ The obtained ultrasmall-sized TMD nanodots would be beneficial for cellular uptake and in vivo metabolism.
(2) In addition, since the electronic/optoelectronic properties of layered nanomaterials are highly sensitive to their thickness, they also possess layer-dependent electronic/optoelectronic properties benefiting from their exfoliation properties.^66,166^ In other words, the electronic band structures of layered nanomaterials can be modulated during the exfoliation process. With layer-dependent and tunable electronic band structures, the bandgap of layered nanomaterials can be regulated by changing the layer numbers or interlayer spacing through external physical or chemical methods, thereby achieving control over material properties, such as tuning the absorbable spectral range from ultraviolet (UV) or visible light to NIR region to boost the PTT, PDT or SDT performance. For example, the micrometer-long MoO_3_ nanobelts with a thickness of 60–130 nm were successfully transformed into defective, thin, short, interlayer gap-expanded MoO_3*–x*_ nanobelts with a thickness of 4–13 nm through lithium treatment.^60^ Compared with MoO_3_ nanobelts, the obtained MoO_3*–x*_ nanobelts with narrowed bandgap exhibited enhanced absorption in the NIR-II region, resulting in a high photothermal conversion efficiency (PCE) of 46.9% at 1064 nm for tumor PTT.

### 3.3 Crystal Phase Tunability

Layered nanomaterials, particularly TMDs, possess diverse crystal phases (e.g., 2H, 1T and 1T’ phases) stemming from their distinct atomic configurations, which directly correlate with their unique physicochemical properties.^46,47,54^ The tunable and even reversible nature of these crystal phases provide great opportunities for the performance customization or optimization of layered nanomaterials, enriching their functional versatility and expanding their applications in the biomedical field. For instance, metallic 1T-phase MoS_2_ nanodots demonstrated strong NIR absorption, whereas the 2H-phase one showed negligible NIR absorption. As a result, 1T-MoS_2_ nanodots gave a PCE of 43.3%, much higher than that of the 2H-phase one with a PCE of 21.3%, achieving enhanced PTT.^59^ Recently, a series of N-doped 1T@2H MoS_2_ with varied degrees of defects were synthesized by adjusting the molar ratios of Mo/S, which exhibited superior reactive oxygen species (ROS) generation performance compared with bulk 2H-MoS_2_, exerting the commendable antimicrobial activity.^167^ This dynamic phase engineering strategies not only optimize material properties but also open avenues for designing multifunctional platforms tailored to specific biomedical challenges, thereby advancing their potential in biomedical innovation.

### 3.4 Ease of Etching Or Doping

Layered nanomaterials, particularly LDHs and MXenes, exhibit significantly higher susceptibility to etching or heteroatom doping compared to traditional materials with a 3D crystal structure. For instance, LDHs, composed of stacked double metal hydroxide layers, are inherently alkaline, rendering them prone to acid-induced etching in environments such as acidic solutions.^38^ Similarly, in M_n+1_AX_n_ (MAX) phase precursors, the weakly bonded A-layer atoms can be selectively etched away using hydrofluoric acid (HF), where these A-layer atoms react with HF to form soluble fluorides, leaving behind the robust M-X layers to form 2D MXenes.^168^ These structural modifications, whether through etching or heteroatom doping, can generate abundant defects, enhance active site density, and induce electronic structure reorganization and/or crystal phase transformation (e.g., crystalline-to-amorphous). Such engineered changes can activate, amplify, or introduce novel functionalities in layered nanomaterials, not only optimizing their therapeutic performance mediated by PTT, PDT, CDT, SDT, or immunotherapy, but also broadening their diagnostic and therapeutic applications in precision cancer treatment, offering versatile platforms for next-generation biomedical technologies. As a typical example, the ROS generation activity of CoW-LDH and NiW-LDH was successfully activated through the simple acid etching, during which crystalline LDH was transformated into amorphous phase, accompanied by defect generation and electronic structure changes (e.g., narrow bandgap, negatively shifted conduction band potential), ultimately achieving high-efficiency SDT.^57^ By etching Ti_3_AlC_2_ MXene, the obtained few-layered Ti_3_C_2_T_*x*_ exhibited superior catalytic activity than the pristine Ti_3_AlC_2_ due to the increased specific surface area and active sites.^169^ The doping of Ca^2+^ into MgFe-LDH not only regulated the energy band and efficiently promoted e^–^–h^+^ separation under US irradiation, but also provoked an immune effect through Ca^2+^-mediated polarization of M1 macrophages and activation of CD8^+^ T cells.^170^

Overall, layered nanomaterials have some unique advantages over traditional materials with a 3D crystal structure in terms of capability as the host to be intercalated with guest species, exfoliation properties, crystal phase tunability, and ease of etching or doping, exhibiting broad application prospects in drug delivery, bioimaging, cancer treatment, theranostics, biosensing, and antibacterial applications. Based on these advantages, structural engineering can modify these layered nanomaterials structurally or engineer them into specific architectures to achieve desired functionalities and meet higher requirements for biomedical applications. Specifically, structural engineering strategies can regulate the electronic properties, physicochemical properties, energy band structure of layered nanomaterials, thus enhancing their performance. Moreover, even inert layered nanomaterials can be activated by structural engineering strategies, thereby endowing them with new activities, properties, functions, and biomedical applications. For example, acid etching and crystalline-to-amorphous phase transformation strategies could significantly activate the activity of LDHs to generate ROS under external stimuli (light, US, etc.), making them a sensitizer for high-efficiency PDT and SDT.^56–58^ Structural engineering brings great potential for promoting the performance of layered nanomaterials to a new level in biomedical applications.

## 4 Structural Engineering Strategies

Owing to the aforementioned significant advantages of structural engineering, this section will systematically review various structural engineering strategies, such as crystal phase engineering, defect engineering, heteroatom doping, interlayer engineering, and crystalline-to-amorphous phase engineering, for engineering layered nanomaterials at the atomic level to tune their crystal phase (e.g., 2H- to 1T-phase), OV (the typical defects in layered oxides), interlayer distances and/or crystallinity, thus optimizing their properties and performance for biomedical applications. We will highlight and compare the advantages and disadvantages of different strategies for the structural engineering of layered nanomaterials.

### 4.1 Crystal Phase Engineering

Phase engineering has emerged as a highly effective and powerful way to enforce the catalytic activities of layered nanomaterials, especially TMDs (e.g., MoS_2_).^46,47,54^ TMDs usually exist as the hexagonal 2H-phase with thermodynamic stability, where the unit cell composed of S–Mo–S monolayer with D_3h_ symmetry, and the active sites locate only at the edges. In contrast, 1T-MoS_2_ holds in an octahedral coordination with thermodynamic instability, in which the active sites proliferate both the edges and the basal plane, thereby boosting its catalytic activity.^66^ The phase transformation from the 2H-phase into the metallic 1T-phase can be achieved by intralayer atomic plane gliding, where one S-layer shifts transversely under harsh conditions to occupy the hollow center of the 2H hexagon.^68^ According to reports, several crystal phase engineering strategies, e.g., intercalation, direct synthesis (atomic doping, solvothermal method, combustion approach), etc., have been developed to engineer the crystal phase of layered nanomaterials for biomedical applications.

#### 4.1.1 Intercalation

Intercalation techniques (e.g., Li-intercalation, molten-metal-assisted intercalation) are conventional methods for achieving the transformation of the 2H-phase MoS_2_ into the metallic 1*T*/1T′-phase. Among them, the most typical one is the Li intercalation strategy, which was first proposed by Joensen et al. in 1986 for the preparation of monolayer MoS_2_.^171^ In the biomedical field, Chou et al. first employed this method in 2013 to prepare chemically exfoliated MoS_2_ with a mixed 1*T*/1T′-phase as a photothermal agent.^154^ The synthesis process includes the Li intercalation followed by ultrasonication. The MoS_2_ powder was immersed in *n*-butyllithium (*n*-BuLi) solution to intercalate Li ions into interlayers of MoS_2_ and then subjected to ultrasonic treatment in water for exfoliation due to the ultrasonication and the formation of H_2_ breaking the weak interlayer forces in bulk MoS_2_. During the intercalation process, the electron transfer from *n*-BuLi to MoS_2_ could induce the transformation of 2H-phase to metallic 1*T*/1T′-phase, thus endowing the MoS_2_ nanosheets with excellent photothermal performance. However, in this study, the crucial role of the metallic phase in enhancing its photothermal performance was not yet revealed. Since then, Li intercalation has been widely used to engineer 2H-phase TMDs (e.g., MoS_2_, MoSe_2_, WS_2_, WSe_2_) into 1T-phase for various biomedical applications.^59,164,172,173^

For example, in 2019, Kim et al. employed this Li intercalation-induced exfoliation method to transform the bulk 2H-phase MoS_2_, WS_2_ and MoSe_2_ nanosheets into few-layered 1T-phase nanosheets, improving their chemical oxidation property and electrical conductivity.^164^ Especially, Zhou et al. reported the preparation of ultrasmall single-layer 1T-phase MoS_2_ (1T-MoS_2_) nanodots from the microsized 2H-phase MoS_2_ crystals by combining simple ball milling process and Li intercalation-induced exfoliation method in 2020 (Figure 4a), and revealed that the photothermal performance of 1T-MoS_2_ outperformed that of 2H-MoS_2_ owing to the metallic phase-enabled strong NIR-II absorption.^59^ Later, Chen et al. also utilized the same method to prepare metallic 1*T*/1T′-phase MoS_2_ nanosheets with US-excited ROS generation activity and NIR-II excited photothermal effect.^172^ It is worth noting that 1T-phase MoS_2_ nanodots/nanosheets prepared by Li intercalation could also be transformed into semiconducting 2H-phase MoS_2_ nanodots/nanosheets through hydrothermal treatment under N_2_ atmosphere. Most recently, Huang et al. also developed 1T-phase MoSe_2_ nanosheets with abundant defects by Li intercalation-assisted sonicated exfoliation method, which not only exhibited evidently enhanced NIR-II photothermal performance owing to the 1T-phase but also amplified oxidative stress by depleting glutathione (GSH) by virtue of rich exposed active Mo centers.^163^

**Figure 4:**
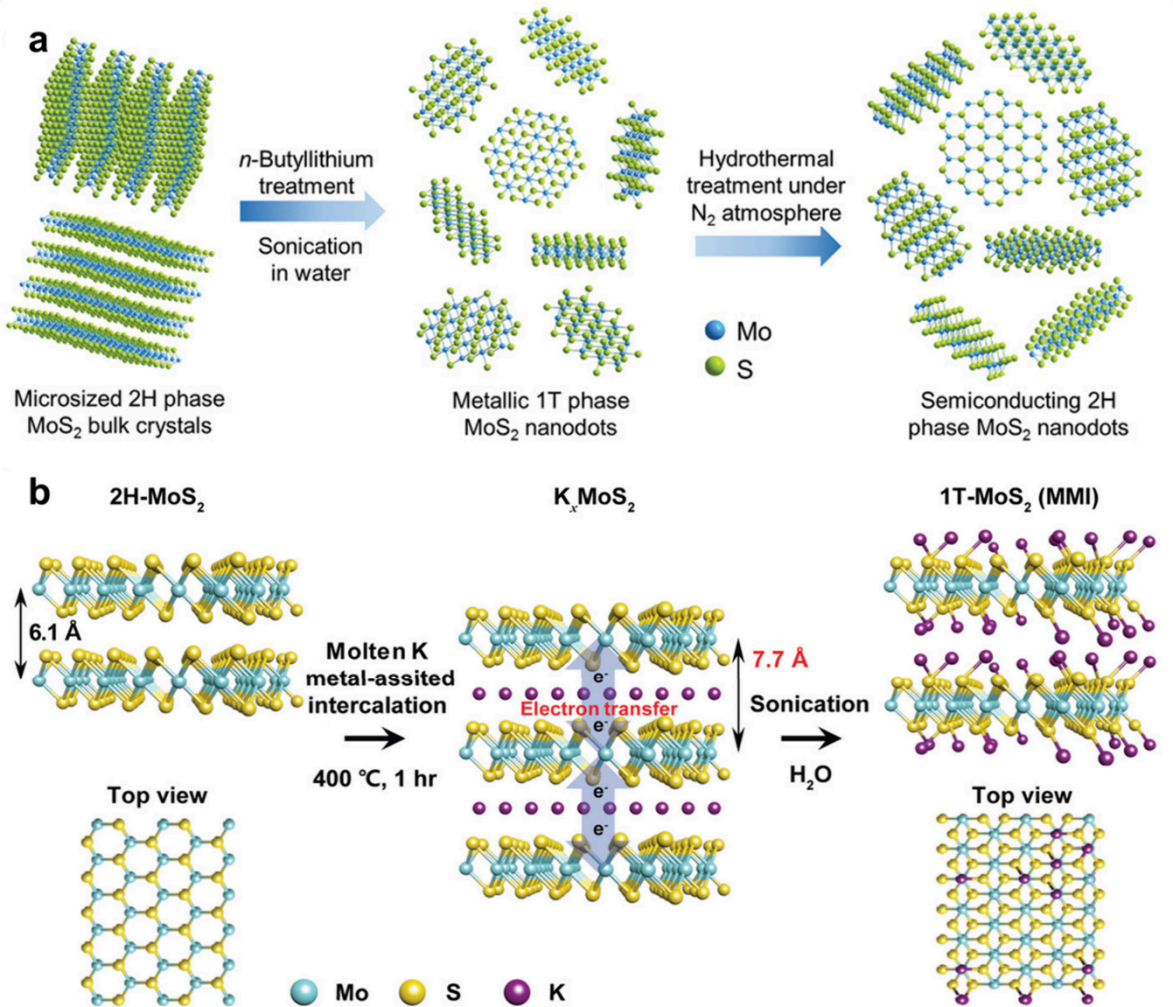
(a) Schematic diagram of preparation of metallic 1T-phase MoS_2_ nanodots from microsized 2H-phase crystals and its further transformation to semiconducting 2H-phase MoS_2_ nanodots. Reproduced with permission from ref 59. Copyright 2020 John Wiley and Sons. (b) Schematic diagram of preparation of 1T-MoS_2_ from bulk 2H-MoS_2_ via the molten-metal-assisted intercalation approach. Reproduced with permission from ref 174. Copyright 2020 John Wiley and Sons.

It can be seen that Li intercalation has strong effectiveness in fabricating metallic 1T-phase or mixed 1*T*/1T′-phase TMDs for biomedical applications. The most prominent advantage of this strategy lies in its operational simplicity, which involves soaking TMDs with *n*-BuLi followed by ultrasonic treatment. However, a critical drawback is the use of *n*-BuLi, a highly flammable and explosive compound in air, posing substantial safety risks. Additionally, the preparation process refers to *n*-BuLi treatment for 48 h and needs to be carried out in the glovebox. Such a long reaction time and violent reaction process are not conducive to the large-scale preparation of metallic TMDs through Li intercalation.

In addition to *n*-BuLi intercalation route, Park et al. also introduced a molten-metal-assisted intercalation approach to synthesize 1T-phase TMDs (1T-MoSe_2_, 1T-MoS_2_, 1T-WSe_2_, and 1T-WS_2_).^174^ This strategy utilized highly reactive molten potassium metal, which was not only efficiently intercalated into the TMDs interlayer but also doped into the basal plane of TMD layers. The d-orbital electron state in the Mo (or W) center was modulated by electron transfer through K–S ionic bonding, resulting in 1T-phase TMDs. Moreover, the suppression of electron emission mediated by K–S ionic bonding ensured a high electron density in the Mo-d (or W-d) orbitals, conferring thermal stability, excellent photostability, and long-term stability. Taking 1T-MoS_2_ as an example (Figure 4b), bulk MoS_2_ and potassium metal were filled into a glass tube and heated at 400 °C, during which the MoS_2_ powder absorbed molten potassium metal via capillary action, resulting in electron transfer from potassium to MoS_2_. The obtained potassium-intercalated MoS_2_ was then exfoliated by sonication to collect the final 1T-MoS_2_. Using the same experimental protocols, 1T-WS_2_, 1T-MoSe_2_, and 1T-WSe_2_ could be synthesized from bulk WS_2_, MoSe_2_, and WSe_2_ powders, demonstrating its universality in the synthesis of 1T-phase TMDs. Interestingly, it was found that 1T-MoS_2_ synthesized by molten-metal-assisted intercalation exhibited much better catalytic performance than 2H-MoS_2_ and 1T-MoS_2_ prepared by *n*-BuLi intercalation, implying the great advantages of molten-metal-assisted intercalation in optimizing the performance of layered nanomaterials. However, the potassium metal employed in this strategy has highly reactive chemical properties and undergoes violent reactions with water, necessitating the use of specialized equipment such as gloveboxes for safe handling. Furthermore, the reaction conditions of this strategy are harsh, as the synthesis process involves heating to temperatures as high as 400 °C.

#### 4.1.2 Direct Synthesis

Different from the above intercalation strategies that involve synthesis of 2H-phase TMDs first followed by intercalation and exfoliation, the direct synthesis strategy can prepare 1T-phase TMDs in one step by adjusting the proportion of raw materials. For example, Basu et al. proposed an atomic doping strategy for stable conversion of the 2H-phase MoS_2_ into the 1T-phase by controlling in situ nitrogen doping in a sulfur-rich environment (Figure 5a).^167^ That is, thiourea solution was mixed with MoO_3_ solution and heated in an oven at a specific temperature (e.g., 200 °C) for a certain time (e.g., 24 h). By adjusting the molar ratios of Mo/S, a series of N-doped 1T@2H MoS_2_ with varied degrees of defects could be obtained, which exhibited superior ROS generation performance in the dark/light compared with bulk 2H-MoS_2_, exerting the commendable antimicrobial activity against *Alternaria alternata*. Similarly, Xia et al. also proposed a phase engineering strategy to prepare 1T-MoSe_2_ nanosheets from the 2H-phase through Co doping (Figure 5b).^175^ With the increase of Co doping concentration, the phase transformation degree of MoSe_2_ from the original 2H-phase to 1T-phase will gradually increase. Notably, in comparison to the aforementioned intercalation strategies, these direct synthesis methods feature simpler preparation steps, relatively mild reaction conditions, and strong feasibility. However, although the proportion of 1T-phase can be enhanced by increasing the doping content of N or Co, the resulting products typically consist of a mixture of 2H- and 1T-phase, making it challenging to obtain pure 1T-phase TMDs.

**Figure 5:**
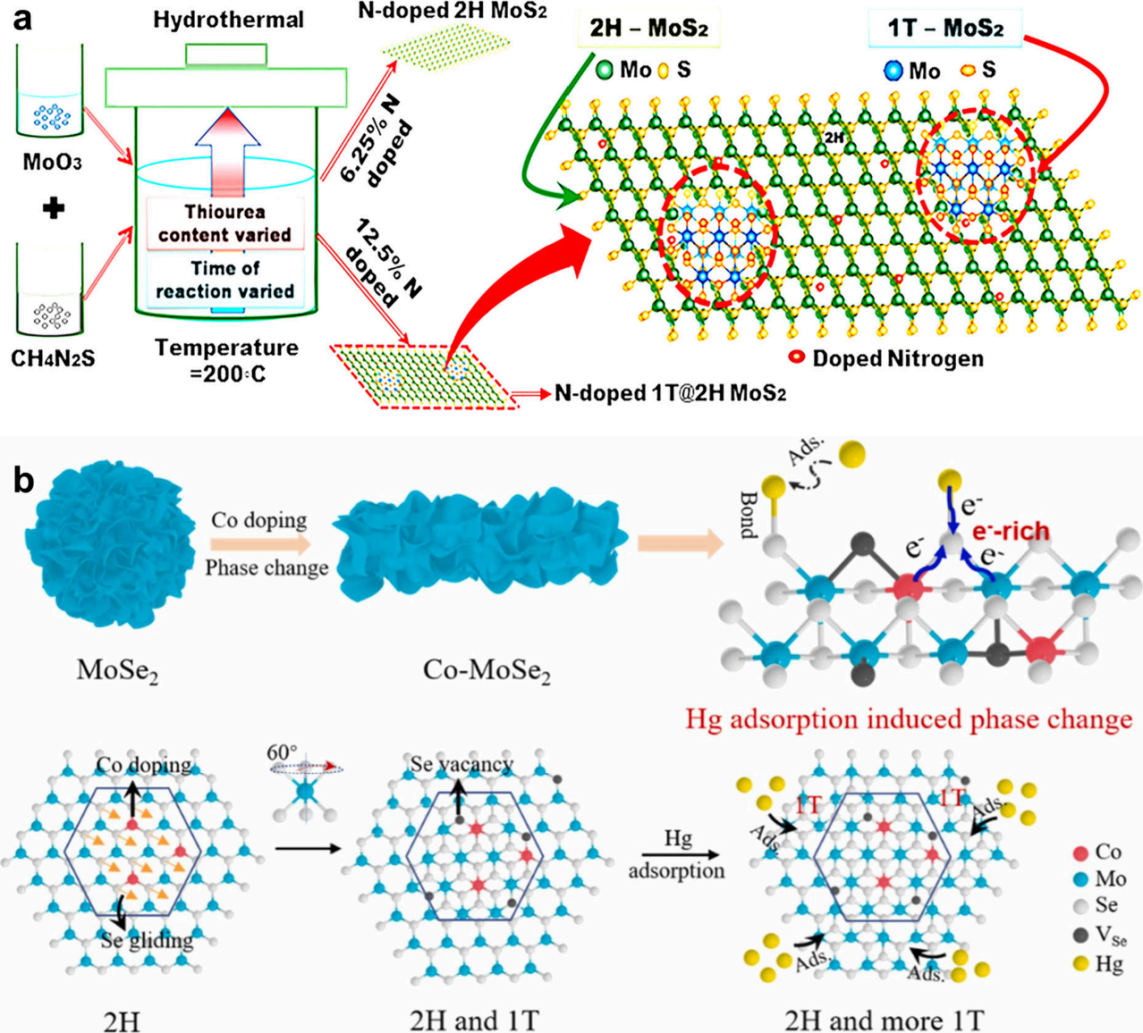
(a) Schematic diagram of synthesis of 1T-phase in 2H MoS_2_ structure through nitrogen doping. Reproduced with permission from ref 167. Copyright 2019 American Chemical Society. (b) Schematic diagram of constructing 1T-MoSe_2_ nanosheets from the 2H-phase through Co doping. Reproduced with permission from ref 175. Copyright 2024 Elsevier.

Recently, Mutalik et al. reported a one-step direct synthesis of pure 1T-MoS_2_ nanosheets through solvothermal method.^176^ In a typical process, thiourea and molybdic acid were dissolved in deionized water before treatment with sonication at a certain time. The resulting homogeneous solution was then heated at a specific temperature (e.g., 180 °C) in an autoclave reactor. In this case, the carbon fiber paper was also placed in the autoclave reactor to provide a platform onto which the 1T-MoS_2_ nanosheets could adhere. In addition, 2H-MoS_2_ nanosheets could be synthesized by subjecting 1T-MoS_2_ nanosheets to a combustion process and annealing it in an incinerator at a high temperature (e.g., 300 °C). Using the same methods, they also successfully prepared 1T-MoS_2_ nanoflowers and 2H-MoS_2_ nanoflowers.^177^ This solvothermal strategy has the advantages of mild conditions, simple operation, high implementability, and the ability to produce pure 1T-phase MoS_2_. However, this method requires the use of additional carbon fiber paper as the substrate material to provide a stable support structure for the uniform growth of MoS_2_, which means that when the synthesized MoS_2_ needs to be peeled off from the carbon fiber paper for collection, there may be a potential risk of yield loss or the introduction of carbon impurities.

#### 4.1.3 Summary

This section provides an overview of recent advancements in crystal phase engineering of layered nanomaterials. Despite significant progress, the field still faces numerous challenges. Although various layered nanomaterials with unconventional phases have been reported, in-depth studies on their crystal phase-dependent properties, performance and applications are still urgently desired. Moreover, stability remains a critical concern. The metastable nature of unconventional phases of layered nanomaterials may give rise to unique physical and chemical properties, while countermeasures need to be taken to stabilize the prepared unconventional phases for further applications. In addition, achieving simultaneous control over both the crystal phase and morphology of layered nanomaterials remains a formidable challenge. For example, the synthesis of nanostructures with identical or similar morphologies but different crystal phases are still difficult, hindering the study of their phase-dependent performance. In-depth understanding of the transformation and formation mechanisms of unconventional phases in layered nanomaterials will help reveal key factors affecting the stability of unconventional phases and pave the way for precise control over their size and morphology. Notably, the synthesis of unconventional crystalline layered nanomaterials primarily depends on the empirical knowledge of researchers, without the support of universal guidelines. To tackle this challenge, a proactive strategy involves identifying and focusing on promising upgradable processes that are currently documented in the literature, especially those that show potential for scalability, address supply demand discrepancies, and prioritize safety. These selected pathways will undergo a systematic prioritization, integrating literature data with experimental validation. The results will then be organized into a structured database, which will serve as a foundational resource for developing future universal guiding principles.

### 4.2 Defect Engineering

In crystal structures, usually there is a considerable density of structural irregularities, commonly referred to as “defects”.^48^ Defects play a crucial role in materials science, significantly influencing properties like thermal, optical, electrical, magnetic, and mechanical behaviors in crystalline materials.^178–181^ By altering electronic structures and chemical properties of layered nanomaterials, defects can introduce new physicochemical characteristics or enhance synergistic effects, thereby optimizing material performance.^182,183^ Therefore, defect engineering is a powerful strategy for tailoring the chemical/physical properties of layered nanomaterials through precise control of defect types and concentrations.^184,185^ Defects can generally be classified into zero-dimensional (0D) point defects (e.g., anion and cationic vacancies), 1D line defects (e.g., dislocation), 2D plane defects (e.g., grain boundaries), and 3D body defects (e.g., lattice disorder and voids).^182^ Among them, vacancy defect is most commonly used in the biomedical field to regulate the biological activity of layered nanomaterials, which involves the elimination of one or more atoms to create more unsaturated active sites.^186–188^ Defect engineering strategies related to vacancies include exfoliation, etching, reduction approach, direct synthesis (one-step hydrothermal treatment, solid-state mechanochemical method, bottom-up method), pulsed laser-mediated method, calcination and quenching process, exfoliation and high-temperature treatment. Here, we briefly discuss how the above strategies have been applied for various layered nanomaterials and their far-reaching effects in biomedical applications.

#### 4.2.1 Exfoliation

As mentioned earlier, a prominent feature of layered nanomaterials is the ability to undergo exfoliation, during which rich defects can be introduced. Sonication-assisted liquid-phase exfoliation is the most simple and efficient strategy for the exfoliation of layered nanomaterials, during which ultrasonic waves provide energy to solvent molecules (e.g., methylpyrrolidone (NMP), ethylene glycol, ethanol), enabling them interacting with the layered nanomaterials to break the weak vdW forces between layers and obtain single-layered or few-layered nanosheets.^189,190^ This strategy was first proposed by Hernandez et al. in 2008, by which they successfully prepared high-quality monolayer graphene.^191^ Since then, this method has been popularized for the preparation of single-layered or few-layered TMDs, layered metal oxides, and α-GeTe nanosheets, which were mainly applied in the fields of electrocatalysis, electronic/photoelectric devices, and energy storage.^189,190,192,193^ In recent years, this strategy has also begun to be applied in the biomedical field.

In 2018, Cao et al. proposed a novel ethylene glycol-assisted liquid exfoliation method to prepare acetaldehyde-modified ultrathin defective g-C_3_N_4_ nanosheets in water.^194^ Typically, bulk g-C_3_N_4_ was dispersed in ethylene glycol under stirring for intercalation and then heated at 160 °C to obtain the acetaldehyde-modified g-C_3_N_4_. The acetaldehyde-modified g-C_3_N_4_ was then ultrasonicated for 10 h to collect ultrathin-layered defective acetaldehyde-modified g-C_3_N_4_ nanosheets. The in situ formation of acetaldehyde in the interlayer not only adjusted the surface energy of g-C_3_N_4_ to better match that of water, thus improving the exfoliation efficiency, but also introduced rich defects into g-C_3_N_4_ acting as excitation energy traps, leading to significant variation in fluorescence emission. Such an environmentally friendly approach simultaneously accomplishes the exfoliation and modification of g-C_3_N_4_ in aqueous solution, offering a versatile method for designing defective carbon nanomaterials. Nevertheless, the process involves multiple steps, including intercalation, in situ hydrothermal treatment and exfoliation, making it relatively more complex compared to other sonication-assisted liquid-phase exfoliation methods that typically require only intercalation and exfoliation steps.

Recently, Yuan et al. prepared OV-rich bismuth-based nanosheets (BiO_2*–x*_) by engineering pristine BiO_2*–x*_ precursor presynthesized via a hydrothermal method through a sonication-assisted liquid-phase exfoliation (Figure 6a).^195^ That is, the pristine BiO_2*–x*_ precursor was added to the exfoliation solvent (50% ethanol solution) in N_2_ atmosphere for deoxygenation and then sonicated in an ice-bath for a certain time (e.g., 8 h). It was found that the OV content increased almost 3-fold after exfoliation (30.47% for BiO_2*–x*_ nanosheets vs. 11.26% for BiO_2*–x*_), which could be attributed to the fact that the thickness of BiO_2*–x*_ nanosheets decreases to the monolayer scale during the exfoliation process, and the more exposed Bi atoms are easier to escape from the lattice, while carrying O atoms out of the lattice, resulting in an increase in defects. The significant elevation of OVs endowed BiO_2*–x*_ nanosheets with numerous active sites, reduced the activation energy barrier of O_2_ dissociation reaction, and promoted electron transfer from electronic orbitals of Bi to O_2_ to form ·O_2_^–^, which is similar to the function of natural oxidases. Similarly, Yang et al. also prepared ultrathin BiO_2*–x*_ nanosheets containing rich vacancies using the same sonication-assisted liquid-phase exfoliation (Figure 6b).^52^ Compared with defect-free BiO_2_ nanoparticles, oxygen-defect-rich BiO_2*–x*_ has a reduced forbidden bandwidth and additional electron-occupying states within the bandgap, providing a higher carrier density and lower adsorption energy of H_2_O_2_ and O_2_. Meanwhile, when exposed to US irradiation, the presence of OVs on the surface of BiO_2*–x*_ trapped electrons and significantly inhibited the recombination of e^–^–h^+^. Accordingly, electrons could be captured by H_2_O_2_ and O_2_ to generate ·OH and ·O_2_^–^, respectively. Liu et al. also synthesized monolayer BiO_2*–x*_ nanosheets with oxygen-rich vacancies via sonication-assisted liquid-phase exfoliation, which not only exhibited effective antibacterial effects using oxidase (OXD)-like activity but also scavenged ROS and mitigated systemic hyperinflammation by mimicking catalase (CAT) and superoxide dismutase (SOD) activities.^196^

**Figure 6:**
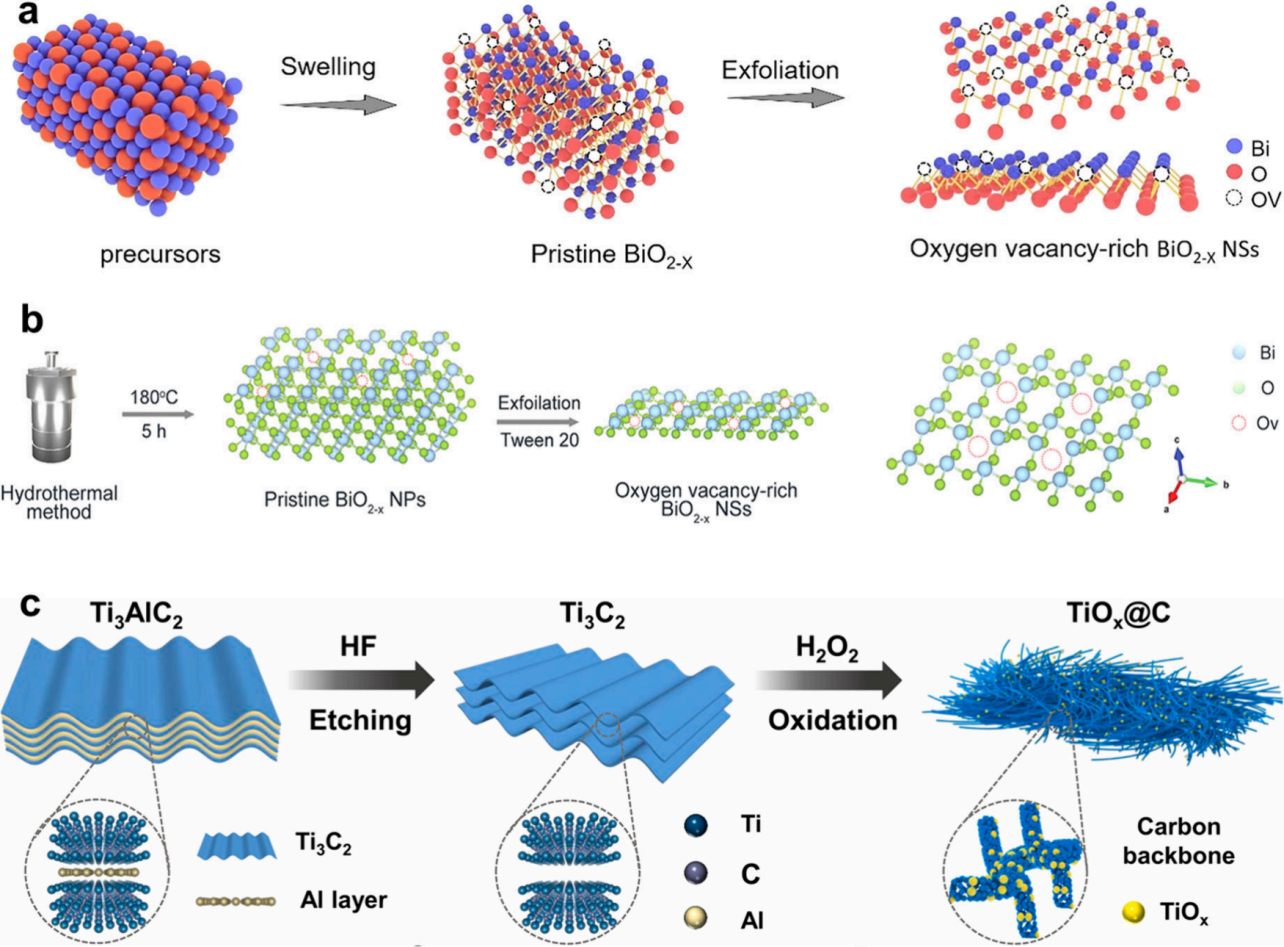
(a) Schematic diagram of preparation of BiO_2*–x*_ nanosheets through sonication-assisted liquid-phase exfoliation. Reproduced with permission from ref 195. Copyright 2021 John Wiley and Sons. (b) Schematic diagram of synthetic process of BiO_2*–x*_ nanosheets. Reproduced with permission from ref 52. Copyright 2023 John Wiley and Sons. (c) Schematic description of preparation of TiO_*x*_@C through HF exfoliation and H_2_O_2_ oxidation. Reproduced with permission from ref 199. Copyright 2023 National Academy of Sciences.

Recently, Miao et al. proposed a safe-by-design exfoliation strategy that integrates cryo-pretreatment with DNA-assisted exfoliation to prepare atomically thin NbSe_2_ nanosheets with abundant atomic defects.^197^ Pristine NbSe_2_ powder was soaked in liquid nitrogen for 1 h to modify its mechanical properties through ultralow temperature treatment. Subsequently, the cryo-pretreatment NbSe_2_ powder was immediately transferred into an aqueous solution of Salmon sperm DNA and treated with alternating bath ultrasonication and probe ultrasonication for 4 h to reduce the layer number and size of NbSe_2_ nanosheets via ultrasonication-induced lattice breakup, resulting in rich atomic defects. The obtained NbSe_2_ nanosheets effectively eliminated various ROS and reactive nitrogen species (RNS) via redox reaction and hydrogen atom transfer. Such high RONS scavenging efficiency likely stemmed from the atomic defects and ultrathin structure of the NbSe_2_ crystals, which down-regulated the adsorption energy. The sonication-assisted exfoliation strategy proposed in this work is implemented in DNA aqueous solution, offering a safer alternative to other exfoliation solvents such as ethanol and enhancing its suitability for biomedical applications. However, this approach also incurs higher preparation costs.

In addition to sonication-assisted liquid-phase exfoliation, Li et al. also proposed a defect repair strategy to construct GSH-Ti_3_C_2_ quantum dots (QDs) as an electrochemiluminescence (ECL) biosensor through microwave-assisted liquid-phase exfoliation.^198^ In a typical process, Ti_3_AlC_2_ dissolved in NaOH solution was heated at 180 °C for 1 h under microwave-assisted condition. The resulting precipitate was mixed with TMAOH solution and GSH solution successively, followed by heating at 110 °C for 1 h under microwave-assisted condition to collect GSH-Ti_3_C_2_ QDs product. The binding of metal atoms on Ti_3_C_2_ to the sulfhydryl group of GSH reduced the defects in the structure, significantly improving the luminescence performance of GSH-Ti_3_C_2_ QDs. This microwave-assisted exfoliation strategy facilitates the rapid completion of the exfoliation process and is relatively straightforward to execute. However, the required microwave equipment is typically costly, and some materials may not be compatible with this method. Notably, the development of larger-scale microwave reactors indicates their potential adoption in routine industrial manufacturing. This structural engineering technique for layered nanomaterials deserves specific focus in future scale-up research.

Additionally, Jiang et al. also reported a defect engineering strategy that combined HF exfoliation and H_2_O_2_ oxidation to fabricate TiO_*x*_ nanoclusters anchoring on amorphous carbon (TiO_*x*_@C) with abundant surface defects (Figure 6c).^199^ Bulk Ti_3_AlC_2_ with Al layer was exfoliated by HF to obtain Ti_3_C_2_ MXene with Ti-deficit vacancies. The redox reaction between H_2_O_2_ molecules and the exfoliated Ti_3_C_2_ MXene promoted the in situ generation of TiO_*x*_ nanoclusters and turned the surface defects into discrete Fenton-like catalytic sites. According to the positron annihilation life spectroscopy that offers information about the relative concentration of defects, Ti_3_C_2_ MXene had a higher amount of surface defects than Ti_3_AlC_2_, while TiO_*x*_@C possessed the highest amount of surface defects, indicating the effectiveness of HF exfoliation and H_2_O_2_ oxidation processes in creating surface defects. The numerous surface defects within TiOx@C significantly promoted ROS generation mediated by Fenton-like catalytic reaction. This work presents a straightforward and versatile strategy for engineering defective Ti_3_C_2_ MXene, which holds potential for extension to other MXene family such as Nb_2_C and V_2_C. However, the HF exfoliation method is highly corrosive and requires stringent experimental conditions and specialized equipment to ensure both safety and experimental efficacy. Additionally, the waste liquids and exhaust gases produced during the HF treatment process pose a certain pollution risk to the environment.

#### 4.2.2 Etching

Etching strategy plays a pivotal role in the fabrication of defect-rich layered nanomaterials and is a crucial step in constructing complex and fine structures, significantly influencing the properties and applications of layered nanomaterials.^200^ Etching strategies encompass a variety of methods, such as traditional etching (HF or high-temperature molten salt etching), low-temperature molten salt etching, one-pot etching, surface confined etching (using chemical or physical methods to locally etch the material surface), dry etching (reactive gases and plasma etching), wet etching (corrosive solution etching), etc., which are primarily utilized in the fields of catalysis, energy storage and conversion, and sensing.^201–203^ With ongoing technological advancements and innovations, certain etching strategies have also been applied to engineer layered nanomaterials for biomedical applications.

Shen et al. engineered ultrathin CoMo-LDH nanosheets through acid etching as a highly effective inorganic PS for NIR-II PDT (Figure 7a).^56^ Specifically, 2D CoMo-LDH nanosheets prepared by hydrothermal method were dispersed in a pH 4.0 buffer solution and etched for 2 h to yield defect-rich CoMo-LDH nanosheets. The effective creation of defects is due to the inherent nature of LDHs as double metal hydroxides, rendering them highly responsive to acidic conditions. The successful acid etching of LDHs is based on acid–base reaction, in which protonation of the M–OH groups occurs and H_2_O molecules form in acidic environments, resulting in the production of rich defects in LDHs. Thanks to the etching-induced electronic structure changing, e.g., narrow bandgap, more negative conduction band position and suppressed e^–^–h^+^ recombination, the defect-rich CoMo-LDH nanosheets displayed enhanced ROS generation performance when exposed to 1567 nm laser with a relative ^1^O_2_ quantum yield of 0.87. Using the same strategy, Zhang et al. prepared defect-rich CoZnW-LDH (DR-CoZnW-LDH) as a highly active PS for NIR-II PDT-mediated Hypertrophic obstructive cardiomyopathy treatment.^204^ Similarly, Li et al. also employed the same acid etching method to prepare defect-rich multifunctional Cu-doped LDH (Cu-LDH) for enhanced acid-responsive PTT.^205^ Due to the use of buffer solution as the etchant in this acid etching strategy, the preparation conditions are mild with low cost. Moreover, the defect density can be regulated by controlling the pH of the buffer solution and the etching duration. However, a notable limitation is the lack of control over the etching direction, making it challenging to accurately tailor the number and site distribution of defects in layered nanomaterials.

**Figure 7:**
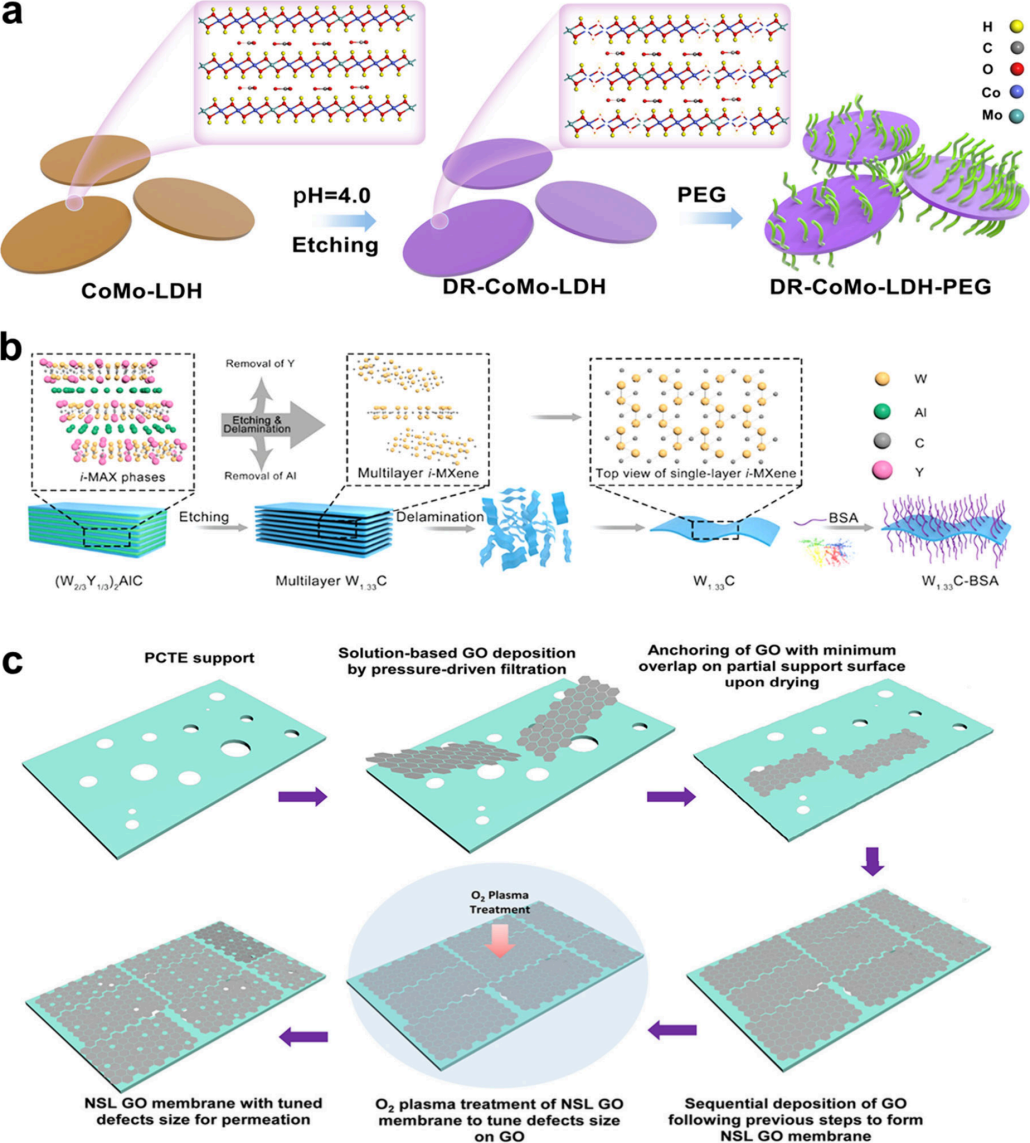
(a) Schematic diagram of the defect engineering of CoMo-LDH nanosheets. Reproduced with permission from ref 56. Copyright 2022 Springer Nature. (b) Schematic illustration of the fabrication of W_1.33_C nanosheets through an etching strategy. Reproduced with permission from ref 207. Copyright 2021 John Wiley and Sons. (c) Schematic diagram of preparation of nominal single-layered GO membrane. Reproduced with permission from ref 211. Copyright 2023 American Chemical Society.

Beyond acid etching via buffer solutions, individual reactants can induce ligand-specific etching in layered nanomaterials based on the Hard–Soft Acid–Base (HSAB) principle. Saikia et al. demonstrated this by synthesizing mesoporous hydroxidic nanosheets. They reacted ZnCr-LDH with β-diketonate ligands (e.g., 2,4-pentanedione, 1,1,1-trifluoro 2,4-pentanedione, 1-phenyl-1,3-butanedione), utilizing their keto–enol tautomerism.^206^ The etching reactivity of these β-diketonate ligands with ZnCr-LDH correlated directly with the enolic acid strength, following the order: 1,1,1-trifluoro 2,4-pentanedione >2,4-pentanedione >1-phenyl-1,3-butanedione. This reactivity gradient dictated distinct structural outcomes: The strongest 1,1,1-trifluoro-2,4-pentanedione caused immediate structural disintegration of ZnCr-LDH at room temperature, resulting in the formation of a segregated Cr^3+^-diketo complex. 2,4-Pentanedione selectively extracted Cr^3+^ ions at 45 °C, generating a retained LDH framework with mesoporous holes. The weakest 1-phenyl-1,3-butanedione failed to etch out Cr^3+^ ions due to insufficient acid strength. According to the HSAB principle, the conjugate base AcAc^–^ from 2,4-pentanedione could act as a hard base and selectively etch out Cr^3+^ (a harder acid than borderline Zn^2+^ due to its higher charge density and smaller ionic radii), thereby enabling the formation of mesoporous ZnCr-LDH.

Recently, Zhou et al. fabricated W_1.33_C *i*-MXene nanosheets featuring ordered divacancies through a HCl/HF etching strategy (Figure 7b) by immersing (W_*2/3*_Y_*1/3*_)_2_AlC *i*-MAX phase precursor into HCl aqueous solution containing LiF for selective removal of Y and Al elements.^207^ Thanks to the ordered basal-plane vacancies, ultrathin 2D W_1.33_C nanosheets featured excellent photothermal performance under both NIR-I and NIR-II laser irradiation with a PCE of 32.5% at 808 nm laser and 49.3% at 1064 nm laser. Similarly, Mao et al. fabricated Ti_3_C_2_[Ti_3_C_2_–SD­(Ti^3+^)] nanosheets containing abundant Ti^3+^ species by etching Ti_3_AlC_2_ powder using HF solution.^208^ It was found that 2D planar defects (slip dislocations) could not only promote the phonon–electron coupling and accelerate electron transfer by virtue of the phonon drag effect, but also reduce the O_2_ activation energy barrier, thereby facilitating ^1^O_2_ generation through the REDOX reaction between electrons and O_2_. It is important to note that HCl or HF is highly corrosive and must be handled under appropriate experimental conditions and with specialized equipment to ensure safety. Additionally, measures must be taken to mitigate the environmental pollution risks associated with the waste liquids and exhaust gases generated during the process.

In addition to acid etching, alkaline etching has also been proposed to create a high density of defects. Zhu et al. applied a biomineralization-assisted bottom-up approach to fabricate defective MoS_2_ QDs (B-QDs), which began with the bottom-up synthesis of pristine MoS_2_ QDs via chemical reactions involving S and Mo ions, followed by alkaline etching and Na^+^ intercalation to obtain B-QDs with rich S-vacancy (SV) defects.^209^ The alkaline etching treatment brought about an increase in defect density, which could be explained as follows: under alkaline conditions (Na^+^ and OH^–^ as the counterion), SH^–^ was deprotonated into S^2–^, forming S^2–^···Mo^4+^···OH^–^···Na^+^. Then, ultrafiltration removed excess ions to yield a neutral colloidal solution of B-QDs, likely generating SH^δ‑^···Mo^4+^···OH^–^···Na^+^. This process restored the defect traps and induced defect increment in B-QDs, leading to an increase in active sites and a decrease in the bandgap energy of MoS_2_ QDs. The narrow bandgap facilitated e^–^–h^+^ separation and strengthened the binding affinity between B-QDs and O_2_, thus promoting the generation of ROS under white light (200–1000 nm) irradiation. The proposed alkaline etching strategy is simple and universal, with potential applicability to other TMDs family.

In addition, oxygen plasma etching has also been demonstrated as an effective method for engineering layered nanomaterials for biomedical applications. This dry etching technique involves the dissociation of oxygen molecules into highly reactive oxygen atoms and ions, which chemically interact with the target material to produce volatile byproducts, thereby achieving etching and introducing defects.^210^ For example, Behera et al. proposed an oxygen plasma etching strategy to precisely tune structural defects on nominal single-layered GO membrane (Figure 7c).^211^ GO was prepared through a modified Hummer’s method. The fabrication of nominal single-layered GO membranes was conducted on a polycarbonate substrate via a sequential deposition approach employing pressure-assisted self-assembly. Subsequently, oxygen plasma etching was performed on the nominal single-layered GO membranes to adjust the size of structural defects on the GO. The structural defects on GO could serve as the major transport pathway for the selective separation of varied-sized proteins (e.g., immunoglobulin G, myoglobin, lysozyme, and bovine serum albumin (BSA)), as the production and/or enlargement of structural defects on GO could generate appropriate pore sizes on nominal single-layered GO membranes. The oxygen plasma etching strategy offers several advantages, including high etching rate, excellent selectivity, and minimal damage to substrate. However, the generation of byproducts such as ozone and carbon monoxide during the etching process may potentially impact both the etching efficiency and the equipment.

#### 4.2.3 Chemical Reduction Method

Chemical reduction method is a simple and facile strategy for introducing defects into layered nanomaterial through a reduction process, typically based on chemical redox reaction between highly reducing reagents (such as hydrogen peroxide, hydrazine hydrate, sodium borohydride (NaBH_4_), etc.) and the material, resulting in the generation of OVs.^212–215^ For instance, Ma et al. prepared bismuth oxyiodide (BiOI) nanosheets using a solvothermal strategy, and then fabricated BiOI-DE nanosheets with oxygen defects and Bi decoration by NaBH_4_ reduction approach.^216^ The proposed defect engineering strategy induced the changes of band structure and electron distribution of BiOI-DE nanosheets, thus expanding its light absorption ability to improve the utilization of light energy and induce hyperthermia. Moreover, the introduction of oxygen defects and semimetal bismuth in BiOI-DE nanosheets was conducive to oxygen adsorption and electron transfer, resulting in a significant amount of ROS. When exposed to 808 nm laser irradiation, the obtained BiOI-DE nanosheets exhibited higher PCE and stronger photocatalytic performance than the pristine BiOI nanosheets. Similarly, Cao et al. fabricated a homologous Bismuth (Bi)-based Schottky heterojunction (Bi-HJ) by NaBH_4_ reduction of Bi_2_O_3_ nanospheres.^217^ The Bi–O bond in Bi_2_O_3_ was much weaker than conventional metal oxides and vulnerable to attack to produce oxygen defects. NaBH_4_ as a strong reducing agent could deprive the oxygen in the Bi–O bond of Bi_2_O_3_ to form Bi-HJ composed of metal Bi and oxygen-deficient Bi_2_O_3_. The generated metal Bi inhibited the recombination of e^–^–h^+^, while the introduced OVs narrowed the bandgap to promote charge separation. Moreover, the localized plasmon resonance effect of Bi-HJ not only promoted rapid electron transfer when exposed to US irradiation, but also enhanced its NIR-II absorption, allowing the implementation of SDT/PTT. Sun et al. designed BiOBr with oxygen-poor vacancies (BBP) and oxygen-rich vacancies (BBR) to evaluate their photocatalytic activities.^218^ The BBP sample was prepared through hydrothermal treatment, while the BBR sample was fabricated from BBP sample by a facile reduction action (BBP was mixed with C_2_H_6_O_2_ and N_2_H_4_·H_2_O under magnetic stirring for 4 h) at room temperature. By virtue of the rich OVs on BBR, the charge carrier lifetime of BBR was increased, and the carrier separation efficiency and O_2_ molecules activation were also promoted in comparison with BBP, generating a substantial amount of ROS under 808 nm laser irradiation, thus significantly improving the photocatalytic antibacterial performance. In general, chemical reduction strategies are typically carried out at room temperature, eliminating the need for expensive equipment. Whereas a notable limitation is that the number of defects formed is highly susceptible to chemical equilibrium.

#### 4.2.4 Direct Synthesis

##### 4.2.4.1 One-Step Hydrothermal Method

Hydrothermal synthesis is a highly versatile and robust chemical synthesis technique that holds significant importance in the fields of chemistry and materials science.^219–221^ Hydrothermal synthesis refers to the chemical reaction of substances in an aqueous solution under high temperature (100 ∼ 1000 °C) and high pressure (1 MPa ∼ 1 GPa) within specialized reactor, such as stainless-steel autoclave, which enables the creation of new compounds and new materials with tailored structures and properties.^222^ Since scientists established the foundational theory of hydrothermal synthesis in 1900, this method has yielded hundreds of crystalline materials for a variety of applications.^223^ In the biomedical field, hydrothermal method is also extensively employed to fabricate defect-rich layered nanomaterials.

For example, Mo et al. constructed a series of CuS nanosheets with abundant sulfur vacancies (SVs) by a one-pot hydrothermal method.^224^ The synthesis process can be summarized as follows: Cu­(CH_3_COO)_2_·H_2_O was mixed with thioacetamide and heated in an autoclave at 180 °C for 20 h. The presence of high concentration V_S_ narrowed the band gap, boosted the light absorption capacity, and increased the e^–^–h^+^ separation efficiency, thereby leading to improved photothermal and photodynamic performance. Chen et al. reported a similar hydrothermal approach for preparing defect-rich MoS_2_ QDs using Na_2_MoO_4_·2H_2_O and GSH as reaction substrates.^225^ The defect sites significantly induced charge transfer between K_2_S_2_O_8_ coreactant and MoS_2_ QDs due to the strong binding affinity of sulfur defects toward SO_4_^•‑^, allowing MoS_2_ QDs to emit NIR ECL emission at ∼810 nm. Jaiswal et al. fabricated defect-rich 2D MoS_2_ nanoassemblies by restricting lattice growth to form atomic vacancies (Figure 8a).^226^ By adjusting the precursor molar ratios of S:Mo (1:1 to 2:1, 4:1, and 6:1), a range of MoS_2_ nanoassemblies with varying defect densities could be obtained. The atomic vacancies on the MoS_2_ lattice planes could act as active sites for vacancy-driven gelation with thiol-activated terminals like poly­(ethylene glycol)-thiol through chemisorption.

**Figure 8:**
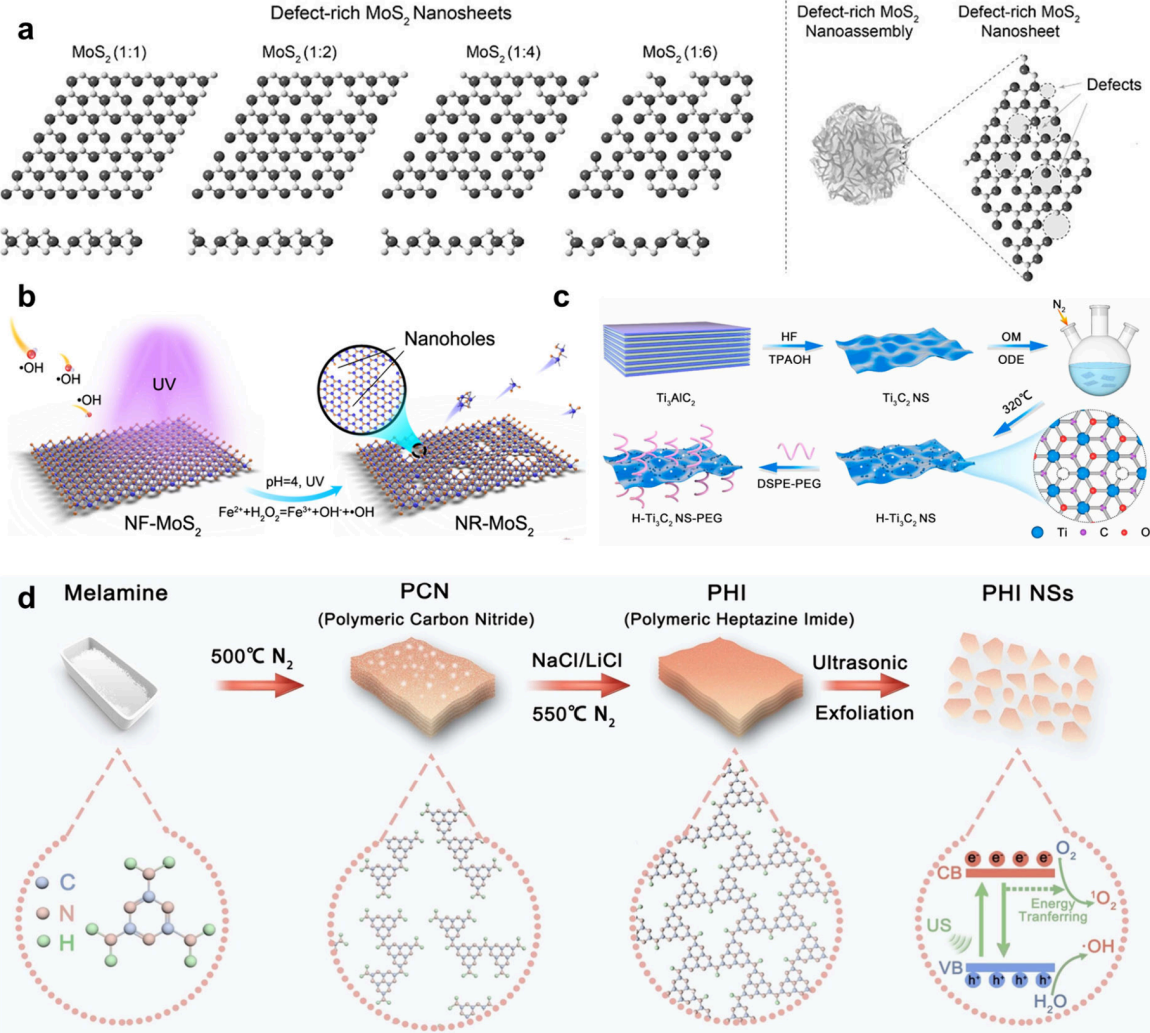
(a) Schematic diagram of MoS_2_ nanoassemblies with different degrees of defects. Reproduced with permission from ref 226. Copyright 2017 John Wiley and Sons. (b) Schematic diagram of preparation of NR-MoS_2_ through UV-Fenton reaction. Reproduced with permission from ref 238. Copyright 2021 Springer Nature. (c) Schematic illustration of the fabrication of H–Ti_3_C_2_ nanosheets by high-temperature treatment. Reproduced with permission from ref 244. Copyright 2022 Elsevier. (d) Schematic diagram illustrating the preparation steps of PHI nanosheets via high-temperature calcination and liquid-phase exfoliation. Reproduced with permission from ref 245. Copyright 2024 John Wiley and Sons.

Guo et al. also designed carbon defect-enriched boron carbide nanomaterial (denoted as B_4_C@C) by one-step hydrothermal treatment of glucose and B_4_C.^227^ There was a carbon layer in the epitaxial region of the B_4_C surface with noticeable distortion at the interface, leading to abundant carbon defects, which could easily capture photogenerated electrons, thus enhancing the NIR-II light absorption. Compared with B_4_C (17.7%), B_4_C@C had a higher PCE (45.4%) under 1064 nm laser irradiation, which was also superior to other carbon-based nanomaterials in the NIR-II region. In addition, Gong et al. proposed a high-temperature organic-phase synthesis strategy to synthesize oxygen-deficient MnWO_*x*_ nanoparticles.^228^ In a typical process, 1,2-dodecanediol, benzyl ether and W­(CH_2_O)_6_ were heated at 120 °C under N_2_ atmosphere. After that, oleylamine and oleic acid were added into the mixture and heated at 260 °C, followed by addition of Mn­(acac)_3_ powder for a 30 min reaction. Compared with nondeficient MnWO_4_ nanoparticles, MnWO_*x*_ exerted a higher ROS (^1^O_2_ and ·OH) generation activity under US irradiation, which could be attributed to the fact that oxygen-deficient sites could enhance the separation of e^–^–h^+^ and MnWO_*x*_ may serve as an electron trap to prevent e^–^–h^+^ recombination. This group also employed the similar method to prepare oxygen-deficient FeWO_*x*_ nanosheets except changing Mn­(acac)_3_ into Fe­(acac)_3_.^229^

It is evident that the hydrothermal method is both simple and convenient, offering additional advantages of high product purity, excellent dispersibility, and ease of particle size control. Nevertheless, hydrothermal synthesis is carried out in a closed reactor, making it impossible to directly observe the crystal growth and material synthesis processes, which complicates research and optimization efforts. Furthermore, the reaction mechanisms governing crystal growth have not been fully elucidated. Existing theories and models are insufficient to comprehensively explain the relationships between crystal structure, defects, growth morphology, and growth conditions, posing significant limitations for practical applications.

##### 4.2.4.2 Solid-State Mechanochemical Method

Solid-state mechanochemical method, first proposed by Gerhard M. J. Schmidt,^230^ is a technique that employs mechanical energy to drive chemical reactions on solid substances, typically involving processes such as ball milling, shearing, and impact, without the need for solvents,^231^ during which abundant defects can be introduced into the materials. For instance, Zhou et al. reported the synthesis of oxygen-deficient tungsten oxide nanobelts (WO_*x*_) using solid-state mechanochemical method.^232^ Specifically, H_2_C_2_O_4_ and Na_2_WO_4_ were uniformly mixed and milled with a planetary ball-mill at room temperature, followed by centrifugation to remove large aggregates to obtain purified WO_*x*_ nanobelts (chartreuse powder). Compared with nondefective WO_3_, defective WO_*x*_ nanobelts showed a narrower bandgap and a more negative conduction band position, resulting in better sonodynamic performance under US irradiation, which could be attributed to the presence of oxygen defects as charge traps to inhibit the recombination of e^–^-h^+^ and enhance the catalytic activity of the excited e^–^ to form ^1^O_2_.

Liu et al. synthesized porous ultrathin g-C_3_N_4_ nanosheets with abundant nitrogen defects by thermal condensation of freeze-dried HCl-pretreated urea. Specifically, urea powders were treated with diluted HCl and then freeze-dried at −50 °C for 12 h.^233^ The resulting powders were heated in a crucible at 550 °C for 4 h, followed by grinding, ultrasonication and centrifugation to obtain porous defective g-C_3_N_4_ nanosheets. Compared with pristine g-C_3_N_4_ nanosheets, the introduction of nitrogen defects endowed g-C_3_N_4_ with more exposed active sites, faster mass diffusion and transfer, enhanced light absorption, improved separation and suppressed recombination of photogenerated carriers. When exposed to visible light irradiation (300 W xenon arc lamp), the defective g-C_3_N_4_ nanosheets mediated the generation of h^+^ and ·O_2_^–^, which could oxidize and tear the cell membrane of *Staphylococcus aureus* (*S. aureus*) and *Escherichia coli* (*E. coli*).

Solid-state mechanochemical method features the following advantages: environmentally friendly: it operates under solvent-free or low-solvent conditions, minimizing environmental pollution; energy efficient: mechanical energy directly activates chemical reactions, reducing energy consumption; easy to operate: no complex equipment or intricate operation steps are required; wide applicability: suitable for the synthesis and modification of a variety of materials. However, the drawback lies in the high requirement of specialized equipment (e.g., high-energy ball mills) and the complexity of parameter control (such as temperature, pressure, ball milling duration, etc.).

##### 4.2.4.3 Bottom-Up Method

Bottom-up method starts from the microscopic atoms, ions or molecules, and polymerizes these microscopic ions into larger cluster structures through certain physical or chemical bonding methods, ultimately assembling and synthesizing the desired nanostructured materials.^234,235^ This method has broad applicability but involves relatively complex procedures. It has been demonstrated that by controlling the feeding ratio of raw materials during the bottom-up synthesis process, defect structures can be effectively formed and regulated. Zhu et al. proposed a biomineralization-assisted bottom-up approach to prepare RuS_*x*_ nanoclusters.^236^ By varying the initial molar ratios of Ru^2+^ and S^2–^, a range of RuS_*x*_ nanoclusters hybridized with different oxygen contents and sulfur defects were obtained. It was found that as the molar ratio of Ru to S increased during synthesis, the insufficient sulfur content limited the reaction between RuO_4_^2–^ and S^2–^, and the residual Ru–O bonds originating from RuO_4_^2–^ were subsequently employed within the RuS_*x*_ structure, resulting in more oxygen substitutions and sulfur defects, further leading to a significant enhancement of PCE. Similarly, Ding et al. fabricated a series of defect-rich MoS_2_ QDs via bottom-up strategy by regulating the relative molar concentration of Mo/S (4:8, 4:4 and 4:2, denoted as MoS_2_-D_L_, MoS_2_-D_M_ and MoS_2_-D_H_).^237^ According to high-resolution transmission electron microscopy (HRTEM) images, strongly disordered arrangement of nanodomains were observed in MoS_2_-D_H_ sample while relatively perfect single crystal without obvious disorder were found in MoS_2_-D_L_ sample. The defective crystal structures were obtained via MoO_4_^2–^ + S^2–^ → MoO_*x*_S_2*–x*_ pathway, where the lower the sulfur content, the less sufficient the reaction, with residual Mo–O bonds originating from MoO_4_^2–^ still embedding in the crystalline MoS_2_ structure, resulting in distortion defects.

#### 4.2.5 Other Strategies

Aside from the aforementioned methods, other strategies such as laser-assisted synthesis, calcination and quenching process, exfoliation and high-temperature treatment, etc., have also been employed to engineer layered nanomaterials with abundant defects. Shi et al. introduced nanoholes into MoS_2_ nanosheets through UV-Fenton reaction to obtain vacancy-enriched MoS_2_ nanosheets (NR-MoS_2_) for antibacteria (Figure 8b).^238^ In a typical process, nanohole-free MoS_2_ powder was mixed with FeSO_4_ and H_2_O_2_, and then HCl was added to adjust the pH to 4.0. The mixture underwent UV-Fenton reaction in the dark for 4 h, followed by freeze-drying for 48 h to obtain nanohole-enriched NR-MoS_2_. The UV-Fenton reaction generated a large number of ·OH, which attacked the Mo–S bond, leading to a V_*x*Mo+*y*S_ structure. Thus, these nanoholes, essentially atomic vacancies of S and Mo, served as electronic donors to enhance the electron transport efficiency between NR-MoS_2_ and biofilms, thereby inhibiting the formation of biofilm.

Joshi et al. reported a pulsed laser-mediated defect engineering strategy to optimize the microscopic and macroscopic defect densities of rGO by varying the energy density and pulse number of excimer laser.^239^ Briefly, laser annealing of the amorphous carbon coatings was carried out using an ArF laser (λ = 193 nm, 20 ns, 0.4–1.2 J/cm^2^) with both single and multiple pulses at ambient temperature and pressure to obtain the defect-rich rGO films. The defects in rGO could serve as active sites for electrocatalytic reactions, which are beneficial for selective and sensitive detection of diverse biologically active chemicals. This laser-assisted synthesis strategy represents an advanced technology characterized by ultrashort processing time (ranging from femtoseconds to milliseconds) and ultrahigh power density. The high-speed and ultraquenching characteristics of laser radiation trigger the breaking of chemical bonds, resulting in the formation of a large number of defects.^240,241^ Notably, the generation of defects depends on the frequency and intensity of the high-energy laser. However, this method involves expensive laser equipment and requires skilled operators. Additionally, laser irradiation may generate a heat-affected zone, which has a certain impact on the properties of materials and also produces harmful byproducts such as smoke and dust.

Wu et al. synthesized three engineered g-C_3_N_4_ QDs (pure g-C_3_N_4_ (CN), g-C_3_N_4_ functioned with a monomer with a rich nitrogen element (CN-THDT), and g-C_3_N_4_ functioned with a monomer with phenyl ending (CN-DPT)) through calcination and subsequent simple quenching process.^242^ The addition of THDT or DPT introduced lattice defects and disordered structure into the g-C_3_N_4_ framework, resulting in defect energy level between the conduction and valence bands to suppress photogenerated carriers recombination and enhanced ROS generation. Most recently, Zhou et al. first reported the synthesis of OV-rich WO_3*–x*_ nanosheets by simple calcination of WO_3_·H_2_O nanosheets presynthesized by hydrothermal method at 400 °C under N_2_ atmosphere.^64^ The introduction of oxygen defects boosted the ROS generation performance of WO_3*–x*_ nanosheets by accelerating the electron transfer and inhibiting the recombination of e^–^–h^+^ under US irradiation.

Du et al. designed CaBi_2_Nb_2_O_9_ nanosheets (CBNO) with adjustable OVs through the in situ hydrothermal treatment with different volumes of glyoxal.^243^ It was found that the concentration of OVs in CBNO-OV_*x*_ nanosheets increased with the addition of glyoxal during preparation. The piezocurrent response maps showed that the introduction of surface OVs could elevate the charge separation efficiency by suppressing e^–^–h^+^ recombination, thus enhancing the piezoelectric catalytic activity of CBNO to generate ROS under US irradiation. Recently, Li et al. developed a new MXene-based sonosensitizer (H–Ti_3_C_2_ nanosheets) by a two-step process of chemical exfoliation and high-temperature treatment (Figure 8c).^244^ That is, Ti_3_C_2_ nanosheets prepared by etching and chemical exfoliation were mixed with oleylamine/1-octadecene and then heated to 320 °C in a nitrogen atmosphere, thereby forming a large number of TiO_*x*_ and OVs. The obtained TiO_*x*_/Ti_3_C_2_ structure not only promoted the separation of US-excited e^–^–h^+^, but also prevented the recombination of e^–^–h^+^ through the capture of e^–^ by OVs, thus exhibiting excellent ROS generation activity under US irradiation.

In addition to strengthening the biomedical-related performances of layered nanomaterials by introducing appropriate surface defects through the aforementioned strategies, the electronic, chemical, and physical properties of layered nanomaterials can also be optimized via defect repair strategy, which involves reducing bulk defects. As a typical example, Yang et al. fabricated defect-repaired g-C_3_N_4_ nanosheets with a poly heptazine imide structure (PHI nanosheets) via high-temperature calcination and liquid-phase exfoliation technique (Figure 8d).^245^ Compared with polymeric carbon nitride (PCN) with numerous defects, PHI nanosheets exhibited stronger ROS generation activity when exposed to US irradiation owing to its fewer bulk defects.

#### 4.2.6 Summary

As previously discussed, there are numerous exemplary cases of defect engineering in regulating the properties of layered nanomaterials. The common defect types include OV, SV and metal cation vacancy, whose functions can be broadly categorized as follows: Band structure modulation: Compared with defect-free layered nanomaterials, those engineered with defects exhibit a reduced bandgap, facilitating e^–^–h^+^ separation. Electronic structure regulation: The formation of OV, SV or metal cation vacancy in layered nanomaterials can yield variable valence metal ions, which generally possess high catalytic, GSH consumption, Fenton reaction activity, or electron trapping capabilities, thereby endowing layered nanomaterials with special properties. Nevertheless, defect engineering also triggers side reactions that may generate impurities and even compromise the structural integrity of layered nanomaterials. More importantly, the synthesis and stabilization of layered nanomaterials with long-range ordered defects remains a significant challenge. Accurately identifying various types of defects and achieving controlled manufacturing of their distribution and orientation continue to be major hurdles. In this regard, substantial efforts are required to further advance defect engineering in layered nanomaterials for broader biomedical applications.

### 4.3 Heteroatom Doping

Heteroatom doping refers to incorporating foreign atoms into layered nanomaterials, which is an effective strategy to modify their physicochemical and electronic properties.^246–248^ Typically, heteroatom doping can be accomplished through two approaches: by introduction/substitution of heteroatoms in the lattice or by surface functionalization with electron-donating or electron-withdrawing molecules.^249^ The success of heteroatom doping in layered nanomaterials has given rise to the design of new layered nanomaterial-based systems for diverse biomedical applications. In this section, we mainly provide a detailed introduction to heteroatom doping strategies, e.g., hydrothermal synthesis, ion exchange method, high-temperature calcination, direct synthesis (one-step hydrothermal method, bottom-up method, coprecipitation method, pyrolysis method, CVD), etc.

#### 4.3.1 Hydrothermal Synthesis

As discussed above, hydrothermal synthesis refers to the chemical reaction of substances in an aqueous solution under high temperature and high pressure within a special reactor, which can produce new layered nanomaterials with specific structures and properties, possessing the advantages of high product purity, good dispersibility, and easy particle size control.^219–222^ In addition to fabricating defect-rich layered nanomaterials, hydrothermal synthesis can also be employed to construct heteroatom-doped layered nanomaterials for biomedical applications. Wu et al. synthesized a highly active S-doped rGO (S-rGO) via a facile hydrothermal reaction by employing GO and Na_2_S as precursors.^250^ The peroxidase-like activity of S-rGO was significantly enhanced in comparison with the pristine rGO, which could be attributed to the sulfur-containing groups providing abundant peroxidase-like active sites. Biswas et al. utilized a similar hydrothermal one-pot technique to prepare Fe-doped rGO nanosheets using FeCl_3_ and rGO as precursors.^251^ The combination of the inherent surface corrugation of 2D graphene sheets and the magnetic entity of Fe could make the Fe-doped rGO nanosheets robust in chemical redox state-mediated antibacterial responses. Thomas et al. also proposed a low-cost and sustainable hydrothermal method for the preparation of N-GO, P-GO and NP-GO from fossil fuel coke (Figure 9a).^252^ Taken NP-GO as an example, GO was first extracted from fossil fuel coke using an improved Hummers’ method, then mixed with phosphoric acid and ethylenediamine, and heated in an autoclave at 180 °C for 12 h. A similar procedure was employed to prepare P-GO and N-GO using phosphoric acid and ethylenediamine as phosphorus and nitrogen sources, respectively. The doping of heteroatoms modulated the emission properties of coke-derived GO, giving rise to extended fluorescence lifetime and increased quantum yield of N-GO, P-GO, and NP-GO. Among them, N-GO achieved better fluorescent characteristic in cellular bioimaging.

**Figure 9:**
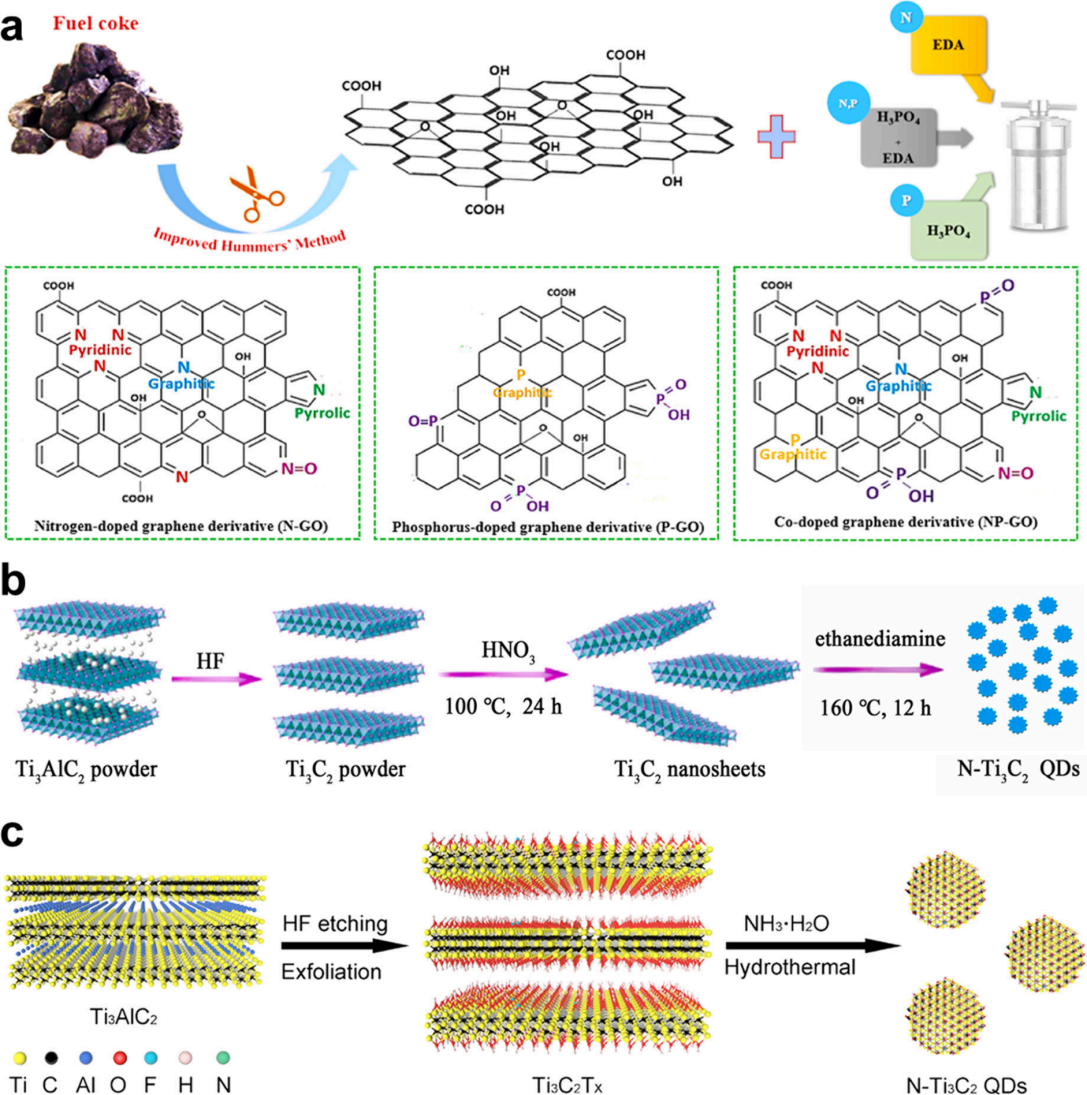
(a) Schematic diagram of the synthesis procedure of N-GO, P-GO and NP-GO from fossil fuel coke through hydrothermal method. Reproduced with permission from ref 252. Copyright 2023 Elsevier. (b) Schematic diagram of preparation of N-Ti_3_C_2_ QDs via hydrothermal method using ethylenediamine as nitrogen source. Reproduced with permission from ref 253. Copyright 2022 Elsevier. (c) Schematic diagram of preparation of N-Ti_3_C_2_ QDs via hydrothermal method using NH_3_·H_2_O as nitrogen source. Reproduced with permission from ref 254. Copyright 2021 American Chemical Society.

Recently, Jiang et al. synthesized N-doped Ti_3_C_2_ QDs (N-Ti_3_C_2_) using a hydrothermal method with ethylenediamine as the nitrogen source and Ti_3_AlC_2_ as the precursor (Figure 9b).^253^ Compared with pure Ti_3_C_2_ QDs, N-Ti_3_C_2_ QDs possessed higher ECL quantum efficiency owing to nitrogen doping. Wang et al. also employed the same method to prepare N-Ti_3_C_2_ QDs using NH_3_·H_2_O as the nitrogen source (Figure 9c).^254^ The doping of N atoms promoted the electrochemical interaction between free radicals and N-Ti_3_C_2_ QDs, thus enhancing their antioxidant performance. Yan et al. synthesized S and N codoped Nb_2_C MQDs (S, N-MQDs) using the following hydrothermal method.^255^ l-cysteine, urea and preprepared Nb_2_C MQDs were mixed into water and transferred to a stainless-steel autoclave at 160 °C for 12 h. The resulting S, N-MQDs exhibited excellent green fluorescence, excitation-dependent photoluminescence, and antiphotobleaching, with a quantum yield of 17.25%. Given these unique characteristics, S, N-MQDs were effectively utilized as a fluorescent probe for cellular bioimaging. Taken together, hydrothermal synthesis offers an exciting avenue for the development of heteroatom-doped layered nanomaterials (e.g., GO, rGO, MXene). It is crucial to note that the reaction mechanisms governing crystal growth have not yet been fully elucidated. Current theories and models are insufficient to comprehensively explain the relationships between crystal structure, heteroatom distribution, defects, growth morphology, and growth conditions, which brings great limitations to practical applications.

#### 4.3.2 Ion Exchange

Ion exchange is another effective method to construct heteroatom-doped layered nanomaterials, especially LDHs. The host matrix of LDHs shows the brucite-like structure consist of edge-sharing octahedral units, with each metal cation coordinated by six hydroxyl groups.^38^ The unique structure enables LDHs to be easily doped with various metal ions (such as Mn^2+^, Zn^2+^, Cu^2+^, Ca^2+^, etc.) to prepare functional nanomaterials. In particular, if the metal cations M^2+^ and M^3+^ have similar radii to Mg^2+^ and Al^3+^, it can preferentially undergo isomorphic substitution from the LDHs matrix to form heteroatom-doped products, i.e. [Mg+M]­Al-LDH or Mg­[Al+M]-LDH.

Zhang et al. and Li et al. reported an ion exchange strategy for synthesizing Mn-doped MgAl-LDH nanoparticles (Figure 10a).^256,257^ Initially, the MgAl-LDH (Mg^2+^/Al^3+^ = 3.0) precursors prepared by coprecipitation method, were mixed with MnCl_2_ solution under vigorous stirring and N_2_ bubbling at room temperature for 12 h. The composition of parent Mg_3_Al-LDH ([Mg^2+^_1–*x*_Al^3+^_*x*_(OH)_2_]^*x*+^[CO_3_^2–^]_*x*/2_·*m*H_2_O, *x* = M^3+^/(M^2+^+M^3+^) = 0.25) was remained within the solid solution range (*x* = 0.2–0.33) established by Allmann et al.,^89^ ensuring structural integrity and uniform host layers suitable for ion exchange. The isomorphic substitution of partial Mg^2+^ in MgAl-LDH by Mn^2+^ endowed it with magnetic resonance imaging contrast capability. Sun et al. also applied the same ion exchange technique to create Cu-doped MgAl-LDH nanoparticles using CuCl_2_ and MgAl-LDH nanoparticles (Mg^2+^/Al^3+^ = 3.0).^258^ The Cu doping introduced coordination defects, enhancing the photothermal performance and ROS generation of these nanoparticles. Moreover, the introduction of Cu^2+^ enabled the occurrence of Fenton-like reaction, thus generating abundant ·OH for CDT. It is important to note that Mg^2+^ (d^0^ electronic configuration and spherical symmetry) forms nearly perfect octahedra in the parent Mg_3_Al-LDH,^259^ while the Jahn–Teller distortion of Cu^2+^ (d^9^ electronic configuration) would disrupt the local lattice symmetry and bond lengths, inducing lattice strain, which theoretically hinders its stable substitution at the high-symmetry Mg^2+^ sites.^260^ When a small amount of Cu^2+^ was introduced, the host layer could adapt to the Jahn–Teller distortion of Cu^2+^ by locally adjusting the bond lengths and angles of adjacent Mg/Al octahedra. The low-concentration Cu^2+^ sites were isolated by Mg^2+^ and Al^3+^ octahedra, and the distortion effect was confined locally without causing the collapse of the entire layer.^261^ Meanwhile, water molecules and interlayer anions may also contribute to stabilizing the distorted CuO_6_ octahedra. Consequently, the isomorphous substitution of Cu^2+^ in the MgAl-LDH layers is a controlled and localized process. Similarly, Zhang et al. fabricated Zn^2+^-doped MgAl-LDH (Zn-LDH) as an immunomodulating adjuvant using the same isomorphic substitution strategy with MgAl-LDH (Mg^2+^/Al^3+^ = 3.0) and ZnCl_2_ as precursors.^262^ The composition of parent Mg_3_Al-LDH, falling within the solid solution range, provided the structural stability basis enabling the isomorphous substitution of Zn^2+^ (d^10^ electronic configuration without distortion) for Mg^2+^. The Zn–OH bonds in Zn-LDH were readily cleaved through hydrolysis in the acidic tumor microenvironment (TME), which could raise the pH of tumor tissues and release Zn^2+^, thereby activating tumor-resident immune cells. Additionally, Zn-LDH could be internalized by cancer cells, where acid-responsive Zn^2+^ release from endo/lysosomes activated the cyclic GMP-AMP synthase-stimulator of interferon genes (cGas-STING) signaling pathway, inducing immunogenic cell death (ICD).

**Figure 10:**
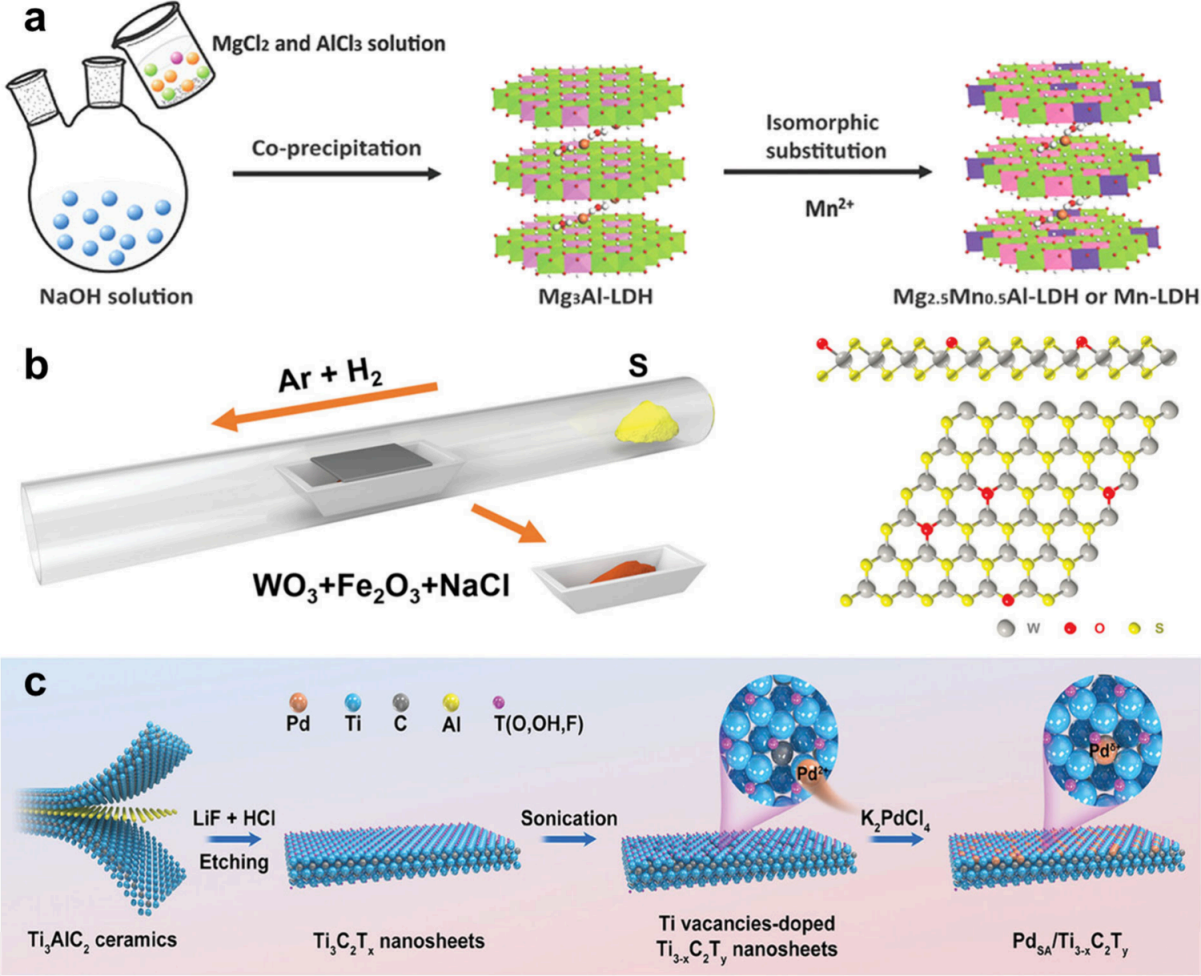
(a) Schematic illustration of synthetic procedure of Mn-doped MgAl-LDH nanoparticles by ion exchange. Reproduced with permission from ref 257. Copyright 2017 John Wiley and Sons. (b) Schematic diagram for the synthesis of O-doped WS_2_ monolayers via CVD strategy. Reproduced with permission from ref 298. Copyright 2021 John Wiley and Sons. (c) Schematic illustration of the preparation of the Pd_SA_/Ti_3*–x*_C_2_T_*y*_ nanosheets. Reproduced with permission from ref 299. Copyright 2024 John Wiley and Sons.

From the examples discussed above, it is evident that the ion exchange method is simple and enables isomorphic substitution at room temperature, eliminating the need for high temperature, high pressure, or specific reaction vessels. However, this method is typically limited to doping metal ions with radii similar to Mg^2+^ or Al^3+^ ions, and it faces challenges in achieving the doping of larger-radius metal ions such as Mo^6+^ or W^5+^.

#### 4.3.3 High-Temperature Calcination

High-temperature calcination, also referred to as high-temperature heat treatment, involves heating materials to elevated temperatures. The calcination temperature typically exceeds 400 °C and can reach several thousand degrees, usually remaining below the melting point of the material.^263,264^ By engineering layered nanomaterials in this way, their molecular structure, crystal structure, and chemical composition can be altered (such as the introduction of heteroatoms or defects) and their chemical and physical properties can be improved.

Yang et al. synthesized a CAT and SOD-mimicking Co-SA-NSG nanozyme consist of atomically dispersed Co and N, S codoped graphene by high-temperature calcining graphene nanosheets and cobalt sulfonated phthalocyanine (TSCoPc) at 800 °C in flowing Ar for 3 h.^265^ In comparison with pristine graphene, Co-SA-NSG nanozyme contained increased defects owing to S and N doping, which facilitated the deposition of Co single atoms and further enhancing SOD and CAT-mimicking activities. Kim et al. employed a similar calcination strategy to prepare N- and B-*co*-doped rGO (NB-rGO).^266^ In a typical process, the GO precursor prepared by the modified Hummers method were mixed with melamine and calcinated at 900 °C for 1 h in Ar filled atmosphere to prepare N-rGO. Then, N-rGO and H_3_BO_3_ were mixed together and calcinated at 900 °C for another 1 h in Ar filled atmosphere to obtain NB-rGO. Compared with undoped rGO, NB-rGO exhibited higher peroxidase-like activity due to the increased active site density caused by N and B doping. Zhang et al. also prepared N-doped rGO (N-rGO) through the pyrolysis of poly­(p-phenylenediamine)-rGO (PpPD-rGO) at 800 °C in N_2_ atmosphere for 4 h.^267^ The incorporation of N atoms into the rGO matrix largely improved its physicochemical properties, such as oxidation stability, conductivity and catalytic activity, ensuring the application of N-rGO for electrochemical detection.

Li et al. employed the following procedures to synthesize Zn^2+^-doped g-C_3_N_4_.^268^ The preprepared g-C_3_N_4_ powder and ZnCl_2_ were dispersed in ethyl alcohol and heated to 80 °C. After thorough vacuum drying, the resulting powder was transferred into a crucible and heated to 400 °C in a tube furnace for 4 h to collect Zn^2+^-doped g-C_3_N_4_. It was found that Zn^2+^-doped g-C_3_N_4_ produced more ROS than g-C_3_N_4_ both under 660 nm laser irradiation or 660 nm plus 808 nm laser irradiation. The increased ROS yields could be attributed not only to enhanced optical absorption and e^–^–h^+^ separation with the assistance of Zn^2+^ capturing electrons from the conductive band, but also to the generation of more defects caused by Zn^2+^ doping. Recently, Wu et al. designed and fabricated Zn^2+^, K^+^ codoped g-C_3_N_4_ (MCN) nanosheets through calcination and ultrasonication-assisted exfoliation.^269^ Melamine was ground with KCl and ZnCl_2_ and then calcined to 823 K under constant Ar flow for 2 h. The product was stirred with KOH solution overnight and then sonicated for 12 h to obtain MCN. The doping of Zn^2+^ and K^+^ expanded the absorption edge of MCN nanosheets from 460 to 663 nm with a decrease in bandgap to 1.94 eV, optimized charge carrier separation and transport, thereby inhibiting e^–^–h^+^ recombination and enhancing ROS generation.

Apart from fabricating heteroatom-doped carbon-based layered nanomaterials, the high-temperature calcination strategy is also suitable for MXenes and TMDs. For example, Deng et al. designed a gallium-doped V_2_C MXene nanoenzyme (Ga/V_2_C) through a thermal sintering process.^270^ That is, the V_2_C powder was mixed with liquid gallium and calcined in a muffle furnace at 400 °C for 3 h. The successful doping of gallium into V_2_C MXene via a lattice substitution mechanism could enhance the NIR laser absorption ability of V_2_C MXene, thereby elevating its PCE. Recently, Yang et al. prepared Fe-doped MoS_2_ (Fe–MoS_2_) through the calcination of MoS_2_ nanosheets and Fe-powders at 550 °C under N_2_ atmosphere for 2 h.^271^ The obtained Fe–MoS_2_ with low-valence Mo^δ+^ (0 < δ < 4) exhibited significantly improved Fenton catalytic activity. Huang et al. constructed Er-doped WSe_2_ nanosheets by calcination-induced cation exchange and ultrasonication-assisted liquid-phase exfoliation.^272^ The doping of Er ions enabled the luminescence of WSe_2_ nanosheets to extend into the NIR-II window.

High-temperature calcination offers dual benefits: it not only enhances the purity and quality of raw materials but also significantly improves their thermal stability while allowing for precise regulation of their chemical composition and/or crystal structure. This process consequently expands the material’s application scope and increases its value. However, calcination presents several challenges, including substantial energy consumption, the need for specialized equipment, and the generation of harmful exhaust gases. Furthermore, while the calcination stage typically lasts only a few hours, the subsequent cooling stage requires a considerably longer duration, resulting in an extended overall processing time.

#### 4.3.4 Direct Synthesis

##### 4.3.4.1 One-Step Hydrothermal Method

Beyond the aforementioned approach of incorporating heteroatoms into precursors, hydrothermal synthesis offers a more straightforward alternative, enabling the one-step fabrication of heteroatom-doped layered nanomaterials. Jana et al. prepared a Cu-doped MoO_*x*_ (CMO) nanozyme using a hydrothermal method.^273^ Briefly, CuCl_2_ solution was added to a mixed solution of Na_2_MoO_4_·7H_2_O and tannic acid. After adding HCl, the mixture was sealed into a Teflon-lined autoclave to crystallize at 180 °C for 10 h. The coexistence of abundant OVs and Cu substitutions within a single nanostructure provided cooperative active sites, resulting in a high efficiency nanozyme for ROS generation. Moreover, Cu doping engineering led to a strong NIR-II absorption of CMO due to the intensified electron delocalization, resulting in significant photothermal effect under 1064 nm laser irradiation. Similarly, Yu et al. synthesized Fe-doped MoO_*x*_ (Fe-MoO_v_) nanozyme using the same method.^274^ The resulting Fe-MoO_v_ nanozyme possessed reinforced enzyme-mimicking activity, which could be attributed to the partial Fe doping in MoO_*x*_ activating structural reconstruction and generating numerous defect sites, such as OVs and Fe substitution. More intriguingly, Fe doping-mediated structural reconstruction led to the appearance of plenty of delocalized electrons, inducing significant surface plasmon resonance effect in the NIR-II region. Recently, Fe-doped bismuth tungstate nanosheets (BWO-Fe) with OVs were synthesized by Ding et al. using one-pot hydrothermal method.^275^ Due to the introduction of oxygen defects by Fe doping, the bandgap of BWO-Fe was significantly narrowed, making it easier to be activated by US. Moreover, Fe doping conferred BWO-Fe Fenton activity to convert H_2_O_2_ into ·OH, thereby amplifying the ROS generation. Tewari et al. reported a cost-effective and eco-friendly one step hydrothermal route for the preparation of K-doped GO from *Quercus ilex* Fruit.^276^ The doping of K could enhance the optical properties of K-doped GO, resulting in bright blue photoluminescence under UV-light.

##### 4.3.4.2 Bottom-Up Method

As previously outlined, the bottom-up approach operates through the polymerization of microscopic ions into clusters, which subsequently assemble into targeted nanostructured materials.^234,235^ Beyond regulating the feed ratio of precursors to engineer defect structures, this methodology also facilitates precise heteroatom doping within layered nanomaterials. Cheng et al. first developed a bottom-up solution-phase method to fabricate a series of transition metal-doped WS_2_ nanoflakes (such as Co^2+^, Mn^2+^, Ni^2+^, Fe^3+^, and Gd^3+^).^277^ A typical synthesis procedure is described as follows: a mixed solution of WCl_6_:MCl_*x*_ (M = Co^2+^, Mn^2+^, Ni^2+^, Fe^3+^, and Gd^3+^) at desired ratios was added into a mixed solvent of 1-octadecene and oleylamine and heated at 150 °C in N_2_ atmosphere to remove O_2_ and water. Afterward, the temperature of the mixture was rapidly raised to 300 °C under N_2_ protection, followed by the addition of sulfur solution vigorously stirring for 30 min to obtain WS_2_:Co^2+^, WS_2_:Mn^2+^, WS_2_:Ni^2+^, WS_2_:Fe^3+^, and WS_2_:Gd^3+^ nanoflakes. Lei et al. also employed a similar bottom-up organic-solution method to construct Fe-doped VS_2_ nanosheets (Fe-VS_2_), demonstrating the universality of this strategy on TMDs.^278^ With Fe doping, the sonodynamic performance of Fe-VS_2_ nanosheets was significantly strengthened due to the prolonged e^–^–h^+^ recombination time, accompanied by the introduction of Fenton catalytic activity.

Peng et al. also reported a new bottom-up method to fabricate Gd^3+^-doped MgAl-LDH nanosheets, where Gd^3+^ ions were located in the LDH host matrix, endowing LDH with magnetic resonance imaging (MRI) contrast ability.^160^ Simply putting, a Mg/Al/Gd nitrate salt solution and a NaOH solution were slowly added into NaNO_3_ solution containing 25% formamide, and stirred at 80 °C for 0.5 h. Similarly, Liu et al. employed a bottom-up method with the aid of H_2_O_2_ for solvent-free synthesis of ultrathin Mn-doped LDH nanosheets, in which O_2_ molecules stemmed from in situ H_2_O_2_ decomposition facilitated the complete exfoliation of LDH layers. Specifically, Mg/Al/Mn chloride salt solution was added into NaOH solution containing H_2_O_2_ to form Mn-LDH slurry, followed by heating in a Teflon-lined stainless-steel autoclave at 100 °C for 24 h to obtain final Mn-doped LDH nanosheets. The doping of Mn ions significantly contributed to GSH consumption and ·OH generation. Employing the similar method, Mei et al. successfully prepared Gd^3+^/Yb^3+^ codoped MgAl-LDH monolayer nanosheets.^161^ It should be noted that while the bottom-up method demonstrates broad applicability across various material systems, its implementation typically involves relatively complex operational procedures.

##### 4.3.4.3 Co-Precipitation Method

Coprecipitation represents a significant and versatile approach for the direct synthesis of layered nanomaterials with homogeneous chemical composition through solution-based chemical reactions.^279^ This method involves the addition of a precipitant to a solution containing two or more homogeneously distributed cations, initiating a precipitation reaction that converts the dissolved ions into insoluble compounds, thereby isolating and concentrating the target material.^280,281^ Historically, coprecipitation gained prominence in radiochemistry when Marie and Pierre Curie employed this technique to separate and extract polonium and radium from pitchblende ore.^282^ With advancements in nanotechnology, coprecipitation has evolved into a widely adopted technique for fabricating diverse layered nanomaterials, offering the capability to introduce defects or heteroatoms for applications spanning catalysis, electrochemistry, energy storage, and biomedicine.^283–285^ The primary advantage of coprecipitation lies in its ability to directly produce nanomaterials with uniform chemical composition, controlled particle size, and homogeneous distribution through various solution-phase chemical reactions. Whereas the shortcomings of this method include relatively low selectivity, operational complexity, multiple experimental steps, and stringent requirements for high-quality precipitants.

Wang et al. employed a coprecipitation strategy to prepare Mn-doped MgFe-LDH (MMF) by adding NaOH solution as a precipitant into Mg/Mn/Fe nitrate salt solution and heating at 65 °C for 24 h.^286^ It was found that Mn substitution modulated the Fe–O bond length to activate H_2_O_2_ for generating O_2_, narrow the band gap for facilitating e^–^–h^+^ separation, and prolong the lifetime of e^–^–h^+^ under US irradiation, endowing MMF with efficient ROS generation activity. Wu et al. synthesized Ca-doped MgFe-LDH (MCF) using a similar coprecipitation approach.^170^ The doping of Ca^2+^ not only effectively adjusted the energy band of MCF and enhanced e^–^–h^+^ separation under US irradiation, but also induced an immune response through Ca^2+^-mediated polarization of M1 macrophages and activation of CD8^+^ T cells.

##### 4.3.4.4 High-Temperature Calcination

High-temperature calcination enables heteroatom doping through two distinct approaches: either by subjecting presynthesized precursors and heteroatom sources to thermal treatment, or by directly heating a homogeneous mixture of all constituent raw materials at elevated temperatures. Liu et al. reported a flash pyrolysis method for the synthesis of a series of metal–nitrogen-doped graphene (MNGR: ZnNGR, FeNGR, CuNGR, CoNGR, and MnNGR) materials.^287^ Specifically, the powders of Zn­(II) zeolitic imidazolate framework (ZIF-8), 1,10-phenanthroline (phen) and transition metal (M­(II)) acetate (M= Fe, Co, Cu or Mn) were mixed together and then placed in a tube furnace at 1050 °C in Ar atmosphere for 1 h. Co-doping of nitrogen and transition metal into graphene could promote the catalytic activity toward nicotinamide adenine dinucleotide (NADH) oxidation. Similarly, Jiao et al. synthesized Fe single atoms on hierarchically S/N codoped porous carbon (FeSNC SACs) by high-temperature calcination, employing colloidal silica as a template.^288^ It was found that FeSNC characterized by unsymmetrically coordinated Fe–N_3_S_1_ sites exhibited much higher peroxide oxidase (POD)-like activity in comparison with FeNC containing Fe–N_4_ sites. This enhancement was due to the S doping-induced electronic/geometric effects, which lengthened the O–O bond distance of adsorbed H_2_O_2_, accelerated electronic transfer between O and Fe, and reduced the energy barrier for forming active intermediate.

Rohaizad et al. fabricated niobium-doped TiS_2_ (Ti_1–*x*_Nb_*x*_S_2_) by controlled heating of elements in stoichiometric ratios.^289^ Simply putting, niobium, titanium, and sulfur were weighed in stoichiometric amounts and mixed into a quartz glass ampule. After melt-sealing with an oxygen–hydrogen welding torch, the ampule was heated in a muffle furnace to 425 °C for 24 h, and then heated to 500 °C for 24 h, 600 °C for 48 h, 800 °C for 48 h, and 850 °C for 12 h to obtain the final Ti_1–*x*_Nb_*x*_S_2_ sample. The doping of niobium with appropriate concentration could enhance the conductivity of Ti_1–*x*_Nb_*x*_S_2_ for biosensing.

##### 4.3.4.5 Chemical Vapor Deposition

CVD is a sophisticated materials synthesis technique that involves introducing two or more gaseous precursors into a reaction chamber, in which they undergo controlled chemical reactions to form new materials that deposit onto a substrate surface.^290–293^ The CVD process typically comprises four sequential stages: (1) vaporization of reactants through heating to achieve sufficient vapor pressure, (2) transportation of the vaporized reactants into the reaction chamber using carrier gases (e.g., argon or hydrogen), (3) surface-mediated chemical reactions leading to the formation and deposition of solid materials on the substrate, and (4) evacuation of gaseous byproducts from the reaction system.^294–296^ This method offers exceptional scalability and enables precise control over complex nanostructures through systematic modulation of process parameters, including precursor composition, temperature, and pressure, thereby tailoring the properties and functionality of layered nanomaterials.^297^ However, CVD presents several technical challenges, particularly the difficulty in controlling rapid gas-phase dynamics and high-temperature effects, which may adversely impact material structure and properties. Furthermore, the applicability of this technique is inherently limited to materials that can be effectively vaporized, excluding certain classes of substances such as refractory metals and nonvolatile organic compounds.

Cui et al. reported a CVD strategy for the synthesis of O-doped WS_2_ monolayers (Figure 10b).^298^ In a typical process, the sulfur and mixture of WO_3_/NaCl/Fe_2_O_3_ were placed in separate crucibles within a two-zone tube furnace, where the sulfur was positioned upstream while the SiO_2_/Si substrate was placed face-down above the crucible containing the mixture. The tube was pumped with Ar gas for 30 min and then the mixture zone was heated to 825 °C within 30 min under Ar/H_2_ atmosphere. When the temperature reached 700 °C, the sulfur zone was heated to 200 °C for 5 min. The final O-doped WS_2_ monolayers were obtained after cooling the furnace. By introducing O atoms into the SV of WS_2_ lattice, the photoluminescence quantum yield was significantly enhanced by nearly two orders to 9.3%.

#### 4.3.5 Other Strategies

In addition, Geng et al. fabricated Pd single atom-doped Ti_3*–x*_C_2_T_*y*_ nanosheets (with abundant Ti vacancies) by adding K_2_PdCl_4_ aqueous solution to Ti_3*–x*_C_2_T_*y*_ suspension (presynthesized by acid etching and sonication) under strong stirring for 4 h at room temperature, in which positively charged Pd^δ+^ single atoms were immobilized at Ti vacancies and coordinated with three nearest C atoms (Figure 10c).^299^ The obtained Pd_SA_/Ti_3*–x*_C_2_T_*y*_ nanosheets gave rise to much higher ROS generation activity than undoped Ti_3*–x*_C_2_T_*y*_ nanosheets, attributing to the fact that the highly atomically dispersed Pd^δ+^ single atoms could function as electron traps to prevent e^–^–h^+^ recombination and also serve as active sites for absorbing O_2_ molecules and transferring US-excited electrons to O_2_. Similarly, Wu et al. designed and synthesized a novel multifunctional Fe­(II)-Ti_3_C_2_ nanoshell by anchoring Fe^2+^ into the layer of ultrathin Ti_3_C_2_ nanosheets through interlayer electrostatic adsorption.^300^ The doping of Fe^2+^ could not only promote the interfacial transfer efficiency of carriers to enhance the lateral conductivity of Fe­(II)-Ti_3_C_2_, but also increased its PCE. While this method demonstrates operational simplicity, its applicability is inherently limited due to the stringent requirement for doped heteroatoms to possess specific physicochemical properties that are compatible with the host material system.

Hai et al. employed a one-pot acid-free microwave method to synthesize boron-doped graphene QDs (B-GQDs) using borax as a boron source and GO as a carbon source.^301^ In a typical process, Na_2_B_4_O_7_ solution was mixed with GO solution and heated at 230 °C in a COOLPEX microwave chemical reaction apparatus for 30 min, followed by filtration, dialysis, and rotary evaporation to collect B-GQDs. B-GQDs showed favorable photoluminescence behaviors due to the doping of boron atoms restoring the defects in the graphene structure. However, this method presents significant operational complexities and requires specialized microwave equipment that typically entails substantial capital investment, making it less feasible for large-scale industrial production.

#### 4.3.6 Summary

Over the past decade, significant advancements have been made in the research of heteroatom-doped layered nanomaterials. Heteroatom doping introduces an additional degree of freedom for precisely modulating the electronic structure and proton dynamics of layered nanomaterials at the nanoscale, thereby facilitating the development of diverse biomedical nanomaterials. These novel heteroatom-doped layered nanomaterials have established a versatile platform enabling multiple biomedical applications, including but not restricted to drug delivery, diagnostic imaging, cancer therapy, theranostics, biosensing, and antimicrobial applications. The future progress in heteroatom doping technology will critically depend on achieving atomic-level precision in controlling both the spatial distribution and concentration of dopant atoms within the layered nanomaterial matrix.

### 4.4 Interlayer Engineering

Interlayer engineering represents a sophisticated approach that involves the intercalation of guest species into crystalline host materials while preserving the fundamental structural integrity of the host layers.^45,51^ Host lattices are typically classified into 1D chain, 2D layer and 3D framework based on their structural characteristics, with 2D layered systems having received the most extensive research attention in previous decades. Recently, there has been a resurgence of interest in intercalation engineering of layered nanomaterials, driven by its significant contributions to both fundamental scientific understanding and technological advancements.^144,145^ A distinctive advantage of layered nanomaterials lies in their inherent vdW gaps, which facilitate the facile intercalation of diverse guest species, including anions, cations, conductive polymers, and organic molecules.^302,303^ This unique characteristic provides an effective strategy for precisely tuning and optimizing the physicochemical and electronic properties of layered nanomaterials, thereby enhancing their application performance across various fields.

The significance of intercalation methods in structural engineering stems from their ability to induce controlled geometric, physical, and chemical perturbations within the crystalline host framework. These perturbations, which can be precisely modulated through careful selection of guest species, enable the customization of host material properties for targeted applications.^143–145^ Furthermore, layered nanomaterials serving as host matrices can reciprocally influence the properties of intercalated guest molecules through nanoconfinement effects, thereby synergistically enhancing performance for biomedical applications.^146,147^ In this context, we present a comprehensive discussion of state-of-the-art interlayer engineering strategies, including hydrothermal treatment, lithium intercalation, aqueous intercalation, direct synthesis, and direct exfoliation for layered nanomaterials, with particular emphasis on their biomedical applications through most up-to-date studies.

#### 4.4.1 Hydrothermal Treatment

Hydrothermal synthesis has emerged as a versatile and robust synthetic approach, offering exceptional flexibility and broad applicability for the fabrication of defect-engineered and heteroatom-doped layered nanomaterials.^226–228,250–255^ This method also enables precise intercalation of guest molecules into the interlayer spaces of layered nanomaterials, making it a powerful tool for materials engineering. For instance, Zhang et al. synthesized porous V_2_O_5_ nanosheets cointercalated with Ce ions and PANI using a one-step hydrothermal method.^304^ Simply putting, porous V_2_O_5_ precursor and H_2_O_2_ were dispersed in deionized water, followed by addition of Ce­(NO_3_)_3_•6H_2_O and aniline. The resulting mixture was heated in an autoclave at 120 °C for 24 h to collect the final product (Ce/PANI/V_2_O_5_ nanocomposites). The cointercalation of Ce ions and PANI greatly increased the interlayer spacing of porous V_2_O_5_ nanosheets. Moreover, the synergistic interaction between Ce ions and PANI notably enhanced the electrical conductivity of porous V_2_O_5_ nanosheets, thus enhancing their structural stability and electrochemical kinetics.

#### 4.4.2 Lithium Intercalation

As previously discussed, Li intercalation represents the most conventional technique for engineering 2H-phase TMDs into 1T-phase.^59,164,172,173^ Besides, this strategy has been proven effective in activating the photothermal performance of layered metal oxide such as MoO_3_ and WO_3_ through interlayer engineering. Zhou et al. first reported the intercalation engineering of layered MoO_3_ nanobelts through lithium treatment to activate their NIR-II absorption.^60^ In a typical process, micrometre-long crystalline MoO_3_ nanobelts prepared by hydrothermal method were grounded in a ball-milling pot and then immersed in an *n*-BuLi hexane solution under stirring for 10 min to obtain the defective, thin, short, and interlayer-expanded MoO_3*–x*_ nanobelts, which exhibited the high PCE of 46.9% under 1064 nm laser irradiation. Such a lithium treatment strategy can also be employed to engineer layered WO_3_ nanoplates. The primary advantage of this method lies in its operational simplicity, however, it is significantly constrained by the inherent safety risks associated with the use of *n*-BuLi, which is not only highly toxic but also pyrophoric and potentially explosive upon exposure to air.

#### 4.4.3 Aqueous Intercalation

Apart from Li intercalation, the same group also reported aqueous intercalation as another promising approach to enhance the enzyme-mimicking catalytic activity of layered MoO_3_ nanobelts.^53^ Micrometer-long single-crystalline MoO_3_ nanobelts were ground using dry ball milling and subsequently intercalated with H_2_O and Na^+^ through a reaction with sodium dithionite and sodium molybdate in aqueous solution, yielding the short NH-MoO_3*–x*_ nanobelts. The cointercalation strategy not only induced interlayer expansion but also generated abundant defects and partially reduced Mo^6+^ to Mo^5+^ in MoO_3_ nanobelts. Furthermore, this approach significantly narrowed the bandgap of MoO_3_ nanobelts, enhancing their absorption of NIR-II light. The resulting presence of Mo^5+^, abundant OVs, and an optimized bandgap through intercalation engineering, activated NH-MoO_3*–x*_ nanobelts into highly efficient nanozymes for ROS generation. This activity was further amplified by the photothermal effect under exposure to a 1064 nm laser. Li et al. also demonstrated that aqueous intercalation strategy could activate MoO_3_ nanobelts as a NIR Type I PS.^61^ The structural changes caused by Na^+^/H_2_O cointercalation induced the color of MoO_3_ nanobelts changing from white to dark blue, showing strong NIR absorption. Under 808 nm laser irradiation, the intercalated MoO_3*–x*_ nanobelts exhibited much higher activity for ·O_2_^–^ generation than the pristine MoO_3_ nanobelts, as the narrow bandgap of intercalated MoO_3*–x*_ nanobelts facilitated the electron transfer with surrounding oxygen molecules to generate ·O_2_^–^.

In addition, Hu et al. successfully fabricated PANI- or dye molecule-intercalated MoO_3*–x*_ organic/inorganic superlattices using the above Na^+^/H_2_O cointercalation strategy combined with ion exchange method (Figure 11a).^62,63^ That is, the obtained NH-MoO_3*–x*_ nanobelts were further mixed with aniline solution or dye solution and stirred at room temperature for a certain time. The intercalation of PANI with good conductivity could facilitate electron transport during the MoO_3*–x*_-mediated Fenton-like reaction, while the intercalation of dye molecules (e.g., NB, TPE-I, TPA-I, and PHC-I) endowed MoO_3–*x*_ with the fluorescence imaging capability. Such an aqueous intercalation approach offers several distinct advantages, including operational simplicity, mild reaction conditions, enhanced safety, and environmental friendliness. However, its application scope is considerably limited, primarily effective for the intercalation of layered MoO_3_ and WO_3_.

**Figure 11:**
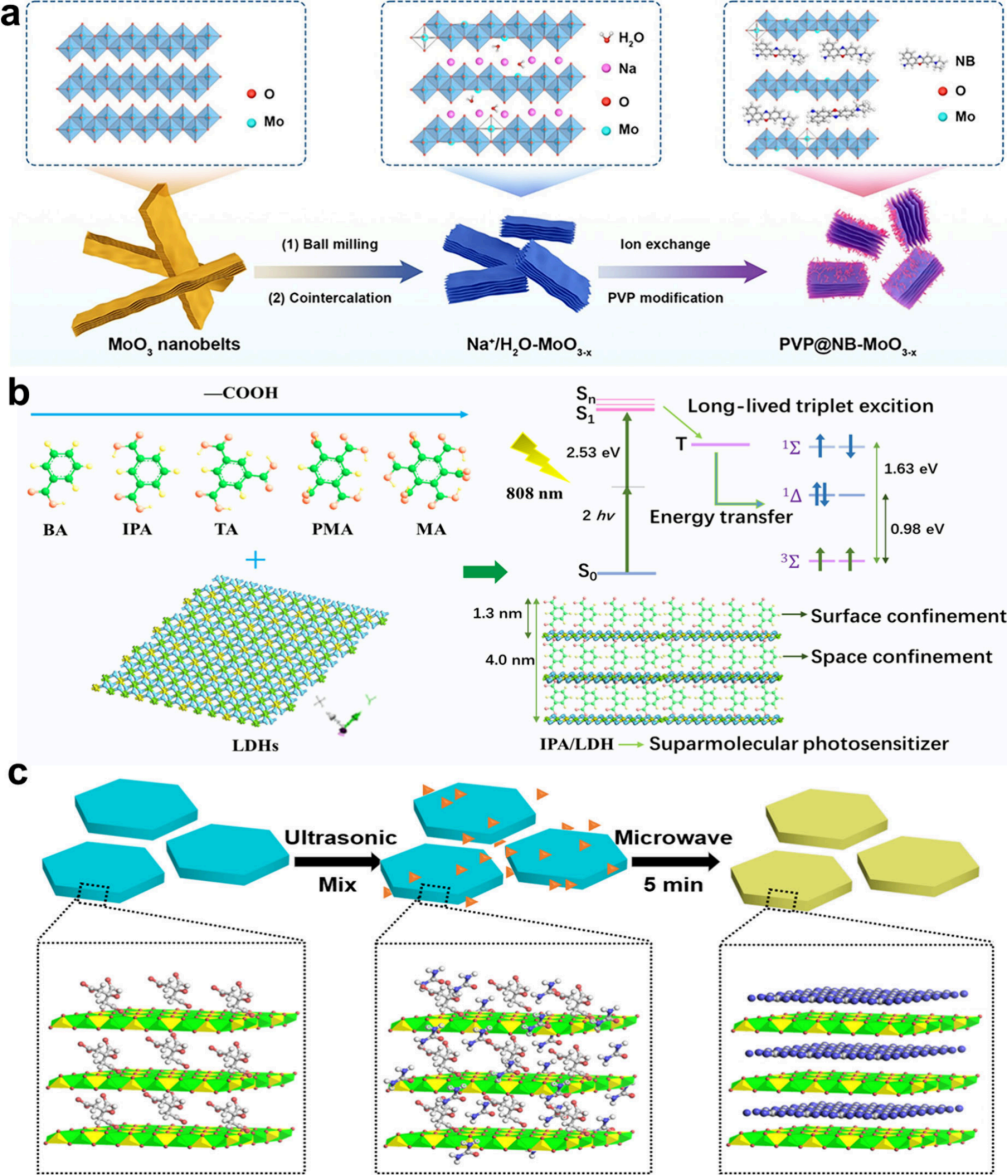
(a) Schematic illustration of preparation of NB-intercalated MoO_3*–x*_ by Na^+^/H_2_O cointercalation combined with ion exchange. Reproduced with permission from ref 63. Copyright 2022 John Wiley and Sons. (b) Schematic diagram of the intercalation of IPA into ZnAl-LDHs. Reproduced with permission from ref 148. Copyright 2018 Springer Nature. (c) Schematic illustration of preparation of CN/LDH by triggering the interlayer condensation reaction between CA and urea. Reproduced with permission from ref 149. Copyright 2018 John Wiley and Sons.

#### 4.4.4 Direct Synthesis

##### 4.4.4.1 Co-Precipitation

Coprecipitation represents a significant and versatile synthesis approach for the direct fabrication of layered nanomaterials, enabling the incorporation of structural defects, heteroatom doping, and guest molecular intercalation during the synthesis process.^279–285^ Gao et al. developed a NIR-I activated supramolecular PS (IPA/LDH) by intercalating IPA into ZnAl-LDHs using a coprecipitation method (Figure 11b).^148^ Zn/Al nitrate aqueous solution was added dropwise to NaOH solution containing IPA, and then heated at 60 °C for 24 h to collect IPA/LDH. Thanks to the surface- and space-confinement effects of rigid LDH sheets, the intercalated IPA molecules were arranged orderly in LDH interlayer space with prolonged triplet excited lifetime, resulting in efficient ^1^O_2_ generation under 808 nm laser irradiation with a ^1^O_2_ quantum yield of 0.74. Similarly, Guo et al. and Jeyakumar et al. also employed this coprecipitation method to prepare nitroprusside-intercalated MgMnFe-LDH supramolecular nanoagent for RNS-augmented cascade CDT and thymoquinone-intercalated ZnFe-LDH for enhanced osteogenesis.^305,306^

Liu et al. designed and synthesized the ultrathin carbon nitride within the 2D confined interlayer space of MgAl-LDH (CN/LDH) by initiating the interlayer condensation reaction between CA and urea (Figure 11c).^149^ The CA-intercalated MgAl-LDH (CA/LDH) was preprepared via the separate nucleation and aging steps method. In a typical process, Mg/Al nitrate aqueous solution and NaOH solution were concurrently added to a colloid mill and blended for 1 min (3000 rpm). The resulting colloid suspension was crystallized in an autoclave to obtain CA/LDH. After mixing with urea solution, the mixture was heated in a microwave oven to trigger condensation reaction and further heated in an oven to acquire the final CN/LDH sample. The synthesized CN/LDH possessed an absolute solid-state quantum yield of 95.9 ± 2.2% due to the confinement effect of the host–guest interaction and could function as a fluorescent material for fluorescence imaging. Similarly, Bai et al. reported the synthesis of N-doped amorphous monolayer carbon by polymerizing pyrrole within the confined interlayer space of NiAl-LDHs.^150^ It is evident that while the coprecipitation method demonstrates broad applicability across various material systems, it is inherently limited by low reaction selectivity, complex operational procedures, multistep experimental protocols, and stringent requirements for high-purity precipitants.

##### 4.4.4.2 High-Temperature Calcination

High-temperature calcination, a thermal treatment process typically conducted at elevated temperatures exceeding 400 °C, serves as an effective approach for both heteroatom doping and guest molecule intercalation within layered nanomaterials.^263–266^ This thermal process facilitates atomic-level modifications through controlled heat treatment, enabling precise structural engineering of nanomaterials.^267–272^ Yasmeen et al. constructed chlorine-intercalated porous g-C_3_N_4_ coupled with MnO_2_ (MnO_2_/g-C_3_N_4_–Cl) through calcination strategy.^307^ The synthesis steps can be summarized as follows: the mixture of Dicyandiamide and NH_4_Cl was calcined in a muffle furnace at 500 °C for a certain time to obtain g-C_3_N_4_–Cl. Then, the mixture of g-C_3_N_4_–Cl and MnO_2_ nanowires was heated in a muffle furnace at 450 °C for 2 h to collect MnO_2_/g-C_3_N_4_–Cl. Compared with pristine g-C_3_N_4_, MnO_2_/g-C_3_N_4_–Cl possessed enhanced photocatalytic activity, owing to the increase in internal layer conductivity after intercalating Cl as interlayer electron channels and the improvement in charge separation caused by heterojunction formation after introducing MnO_2_ as energy platform. Recently, Liu et al. employed similar calcination method to prepare K^+^ and I^–^ intercalated g-C_3_N_4_ (CN-KCl/KI) by calcining the mixture of melamine, KI and KCl at 550 °C for 4 h.^308^ The ion (K^+^ and I^–^) intercalation could enhance the carrier separation and transfer in g-C_3_N_4_, thereby boosting its photocatalytic performance. It should be emphasized that the calcination process necessitates substantial energy consumption and specialized instrumentation, while simultaneously generating environmentally hazardous exhaust emissions. These inherent limitations pose significant challenges for large-scale industrial applications and require careful consideration of energy efficiency and environmental impact mitigation strategies.

##### 4.4.4.3 One-Step Hydrothermal Method

In contrast to conventional hydrothermal synthesis, which requires presynthesized precursors and guest molecules for intercalation processes, the one-step hydrothermal approach enables direct interlayer engineering through simultaneous thermal treatment of raw layered nanomaterial constituents and guest molecules within a single reaction system. Moses et al. reported a one-step hydrothermal method for the preparation of polyvinylpyrrolidone (PVP)-intercalated 1T- and 2H-WSe_2_ nanosheets.^309^ Se powder, WCl_6_ and PVP were dissolved in N, N-dimethylformamide and then heated in an autoclave at 220 °C for 24 h to obtain 1T-WSe_2_@PVP nanosheets. 2H-WSe_2_@PVP nanosheets were prepared by calcining 1T-WSe_2_@PVP at 400 °C for 2 h. The intercalation of PVP enhanced the optical absorption of WSe_2_ nanosheets and also improved their biocompatibility and physiological stability, achieving NIR photothermal tumor treatment.

#### 4.4.5 Other Strategies

Mahar et al. reported the synthesis of V_2_CT_*x*_ with improved interlayer spacing using a soft condition approach, in which HCl/HF mixture was adopted as the etchant and triethylamine (TEA) was employed as an intercalant to delaminate V_2_AlC flakes.^310^ The delamination with TEA resulted in a decrease in interlayer spacing of V_2_CT_*x*_ to 8.13 Å and enhanced its shelf life by 6 weeks. Song et al. and Zhou et al. used a similar method to prepare Li-ion-intercalated Ti_3_C_2_T_*x*_ MXene and polypyrrole (PPy)/phosphomolybdic acid (PMo_12_) coembedded Ti_3_C_2_T_*x*_ (PPy@Ti_3_C_2_T_*x*_/PMo_12_).^169,311^ Li-intercalated Ti_3_C_2_T_*x*_ MXene exhibited superior catalytic activity than the pristine Ti_3_C_2_T_*x*_ due to the synergistic contributions from expanded interlayer spacing, abundant intercalated Li ions and increased active sites. The PPy@Ti_3_C_2_T_*x*_/PMo_12_ displayed a superior electrochemical sensing to PPy@Ti_3_C_2_T_*x*_ and Ti_3_C_2_T_*x*_@PMo_12_ owing to the synergistic effect among these components. This technique encompasses a two-step procedure involving etching followed by exfoliation (or intercalation), which is comparatively complex and time-consuming.

Kaur et al. proposed a green route for the scalable preparation of defect-free and few-layered MoS_2_ nanosheets through direct exfoliation in pure water.^163^ That is, the commercialized bulk MoS_2_ powder was exfoliated in pure water using a tip sonicator for a certain time, during which the temperature was maintained using an ice–water bath. The few-layered MoS_2_ nanosheets exhibited excellent dispersion stability within 3 weeks and layer-dependent cytotoxic modulation effect. Moreover, MoS_2_ nanosheets with 2–5 layers exhibited significantly enhanced bactericidal effects than bulk MoS_2_ nanosheets by inducing membranes mechanical injury and oxidative stress, which may be due to the fact that few-layered MoS_2_ nanosheets could serve as nanoblades to cut the external cell wall of *Salmonella* as its thickness is less than or approximately the same as that of the walls. This direct exfoliation approach offers distinct advantages in terms of simplicity, operational convenience, safety, and environmental friendliness. However, its application is significantly constrained when dealing with layered nanomaterials characterized by strong interlayer interactions, as it presents substantial challenges for effective interlayer engineering.

Recently, Ning et al. developed a supramolecular assembly strategy to fabricate Fe-TCPP-intercalated NiAl-LDH by combing hydrothermal method, ion exchange, exfoliation, and assembly processes.^312^ In a typical procedure, Ni/Al nitrate salt solution and urea were heated in an autoclave at 190 °C to obtain CO_3_^2–^-intercalated LDH (NiAl-CO_3_-LDH). NiAl-CO_3_-LDH was then dispersed in a mixture containing HNO_3_ and methanol to obtain NO_3_^–^-intercalated LDH (NiAl-NO_3_-LDH). NiAl-NO_3_-LDH was mixed with formamide and agitated vigorously in a mechanical shaker for 2 days, followed by addition of Fe-TCPP solution to obtain the final Fe-TCPP-intercalated NiAl-LDH. The host–guest interaction between NiAl-LDH and Fe-TCPP promoted photoresponsiveness and photocatalytic activity. Similarly, an arsenic-intercalated NiTi-LDHs film was constructed by Xing et al. by combining hydrothermal method, calcination and ion exchange.^313^ The cleaned Ni–Ti discs and the precursor solution containing nickel chloride, titanium tetrachloride, HCl, and urea were heated at 120 °C for 24 h. The resulting NiTi-LDHs film was calcined at 250 °C for 2 h, and then soaked in sodium arsenite solution for 24 h to obtain the final arsenic-intercalated NiTi-LDHs film. The NiTi-LDHs could act as a “processor” to catalyze the oxidation of intercalated As­(III) ions to As­(V) ions due to the formation of Ni^3+^ from Ni^2+^ losing electron and control their responsive release. The methods discussed herein are characterized by intricate processing requirements and inherent limitations in scalability, which significantly hinder their potential for large-scale industrial implementation.

#### 4.4.6 Summary

Owing to the remarkable advantages of layered nanomaterials and significant advances in recent years, interlayer engineering has emerged as a transformative approach for precisely tailoring the structure of layered nanomaterials and altering their physicochemical properties. While significant breakthroughs have been achieved in interlayer engineering of layered nanomaterials, numerous scientific challenges and promising opportunities persist in this field. For example, the intercalation of guest molecules with different functions may tailor the inherent properties of the layered crystal, however, this process may concurrently compromise the structural integrity of the host framework. Consequently, meticulous optimization of both the intercalation stoichiometry and spatial configuration of guest molecules is imperative to achieve an optimal equilibrium between enhanced performance and structural stability. Furthermore, theoretical investigations play a pivotal role in elucidating fundamental principles of interlayer chemistry and establishing robust design paradigms for developing novel layered nanomaterials with tailored functionalities. By predicting the change of electronic band structure and host–guest interaction dynamics via theoretical calculations, it is expected to design and explore innovative intercalated biomedical nanomaterials with optimized performance characteristics and targeted functionalities.

### 4.5 Crystalline-to-Amorphous Phase Engineering

Controlled synthesis of low crystallinity layered nanomaterials, such as amorphous or crystalline–amorphous heterophase, is of great significance for engineering phases beyond ordered atomic arrangements.^314–317^ Compared with crystalline layered nanomaterials characterized by highly ordered microstructures, amorphous layered nanomaterials display only short-range order at a few atoms and lack long-range order.^46^ Due to their significant lattice distortion and unsaturated coordination environment, layered nanomaterials with amorphous phase exhibit better performance than crystalline materials in photocatalysis, electrocatalysis, and batteries.^318–320^ In recent years, amorphous layered nanomaterials have also been explored as attractive nanoagents for diverse biomedical applications. Here, we discuss several proposed strategies for preparing layered nanomaterials with amorphous phase or crystalline–amorphous heterophase, including supercritical CO_2_ treatment, etching treatment, annealing, template-assisted method, hydrothermal synthesis, and *n*-BuLi treatment.

#### 4.5.1 Supercritical CO_2_ Treatment

Supercritical CO_2_ treatment primarily involves the pressurization and heating of carbon dioxide beyond its critical temperature and pressure, thereby inducing and maintaining a supercritical state.^321^ This unique fluid state, characterized by its distinctive physicochemical properties combining gas-like diffusivity and liquid-like density, has demonstrated remarkable potential as both a reaction medium and catalyst in advanced material synthesis.^322^ In recent years, the application of supercritical CO_2_ treatment has gained significant momentum in materials science, particularly in the exfoliation of layered nanomaterials and the modulation of their crystal phase.^323,324^

Liu et al. designed a new method for synthesizing amorphous MoO_3_ nanosheets by combining MoS_2_ oxidation and supercritical CO_2_ treatment.^325^ The typical process is as follows: the preprocessed MoS_2_ was annealed in the air at 350 °C for a certain time to prepare MoO_3_. The obtained MoO_3_ was dispersed in a 45% ethanol aqueous solution and transferred to the supercritical CO_2_ apparatus and heated to 80 °C, followed by charging CO_2_ into reactor to the desired pressure (e.g., 16 MPa) for 3 h of reaction to collect amorphous MoO_3_ nanosheets. The crystal phase transformation of MoO_3_ nanosheets enabled the tunable plasmon resonances in the visible and NIR regions under illumination.

Later, the same group also successfully fabricated partial crystallized 2D VO_2_(D) nanosheets and amorphous VS_2_ nanosheets from bulk VS_2_ by precisely altering the temperature and pressure of supercritical CO_2_.^326^ That is, the crystal VS_2_ prepared from bulk VS_2_ was transferred into the supercritical CO_2_ apparatus and heated to 40/80 °C respectively to obtain VO_2_(D) (80 °C) and amorphous VS_2_ nanosheets (40 °C). This is the first case to directly acquire amorphous VS_2_ nanosheets and achieve the phase transition of crystal VS_2_ to VO_2_ from bulk VS_2_. It was found that the amorphization of VS_2_ under supercritical CO_2_ condition is time-dependent, while its morphology is only related to the supercritical CO_2_ pressure. The supercritical CO_2_-induced crystal phase transformation enabled the broadband absorption, intense photoluminescence, and excellent PCE of VO_2_(D) and VS_2_ nanosheets, which could be attributed to the strong carrier localization and quantum confinement.

Supercritical CO_2_ synthesis offers significant advantages, including nontoxicity, chemical stability, and eco-friendliness. The precise tunability of supercritical CO_2_’s solvation properties through pressure and temperature modulation endows this method with exceptional flexibility and control accuracy. Nevertheless, this technique is constrained by several limitations. The generation of supercritical CO_2_ necessitates stringent high-pressure and high-temperature conditions, resulting in increased equipment complexity and operational costs. Furthermore, the relatively low solubility of certain compounds in supercritical CO_2_ may restrict its applicability across diverse material systems. Despite these challenges, supercritical CO_2_ treatment represents a distinctive synthesis approach with substantial potential for various applications. As technological innovations continue to advance, this methodology is anticipated to expand its impact across multiple scientific and industrial domains.

#### 4.5.2 Etching Treatment

As previously discussed, etching strategy serves as a crucial methodology in fabricating defect-rich layered nanomaterials, which has also been successfully extended to engineer layered nanostructures with precisely controlled amorphous phases or crystalline–amorphous heterophase configurations, particularly for advanced biomedical applications. Hu et al. successfully fabricated ultrathin amorphous CoW-LDH and NiW-LDH nanosheets through etching strategy.^57^ The hydrothermal-synthesized CoW-LDH and NiW-LDH nanosheets underwent a crystalline-to-amorphous phase transformation via acid treatment in phosphate buffered solution (PBS, pH 4.0) at room temperature for 6 h. Successful amorphization can be attributed to the fact that LDHs are essentially a type of double metal hydroxides, rendering them responsive to acidic conditions. The successful acid etching of LDHs is based on acid–base reaction, in which protonation of the M–OH groups occurs and H_2_O molecules form in acidic environments, resulting in decreased crystallinity, distorted lattices, dangling bonds, and rich defects in LDHs and thus achieving phase transformation. Due to the defect generation and electronic structure changes (e.g., narrow bandgap, negatively shifted conduction band potential, inhibited e^–^–h^+^ recombination) caused by phase transformation, amorphous CoW-LDH and NiW-LDH nanosheets possessed superior ROS generation activity than the polycrystalline ones under US irradiation.

Most recently, the same group further proposed a series of metal-doped amorphous CoMo-LDH nanosheets (a-M-CoMo-LDH, M = Zn, Mg, Ni, Al, Cu, Mn) through a combination of one-step hydrothermal and acid etching methods.^327^ The generation of a significant quantity of defects and the reduction of bandgap caused by metal doping and acid etching synergistically strengthened the ROS generation performance of LDH. Especially, a-Zn-CoMo-LDH nanosheets exhibited the highest ROS generation activity compared with other a-M-CoMo-LDH nanosheets under 1270 nm laser irradiation with a ^1^O_2_ quantum yield of 1.86, which is the highest among all the reported PSs. Using the same strategy, Cao et al. prepared amorphous CoBiMn-LDH (a-CoBiMn-LDH) and amorphous CoBiFe-LDH (a-CoBiFe-LDH) nanoparticles as multifunctional sonosensitizers.^328,329^

In addition, inspired by the weak acidity of the TME and the production of lactic acid by probiotic metabolism, Yang et al. also reported a TME-responsive PS (*LA*&LDH) through the coupling of *Lactobacillus acidophilus* (*LA*) with CoCuMo-LDH nanosheets.^58^ CoCuMo-LDH nanosheets underwent a crystalline-to-amorphous transformation via in situ etching triggered by *LA* metabolite-induced low pH and overexpressed GSH, which markedly boosted its ROS generation efficiency when subjected to 1270 nm laser, achieving a relative ^1^O_2_ quantum yield of 1.06. Such an acid etching strategy demonstrates remarkable mildness and cost-effectiveness, primarily attributed to the utilization of buffer solutions as etching agents. Moreover, the amorphization degree can be precisely modulated through systematic control of both buffer solution pH and etching duration. Nevertheless, a significant limitation lies in the inherent nondirectionality of the etching process, which poses substantial challenges in achieving precise control over defect density and spatial distribution within the resulting amorphous layered nanomaterials.

In addition, Wei et al. successfully tailored the phase structure of Nb_2_CT_*x*_ MXenes, transitioning them from hexagonal to amorphous through time-dependent persulfate oxidation based on an optimized Lewis acidic molten salt etching strategy.^330^ The hexagonal Nb_2_CT_*x*_ MXenes were preprepared by an optimized Lewis acid etching route, and then reacted with ammonium persulfate solution for varying durations to regulate their crystallinity. For instance, a 2 h duration yielded crystalline Nb_2_CT_*x*_, while a 12 h reaction resulted in amorphous Nb_2_CT_*x*_. This etching strategy employs molten salts (usually Lewis acid salts) to selectively etch MAX phase materials, resulting in the precise removal of the A layer and subsequent generation of MXenes. The technique enables effective modulation of MXenes’ surface functional groups, thereby tailoring their physicochemical properties, while offering significant advantages in terms of process efficiency, operational safety, and environmental friendliness. Furthermore, the amorphization degree can be precisely controlled through systematic regulation of reaction duration.

#### 4.5.3 Annealing

Beyond the aforementioned strategies, annealing represents another robust approach for synthesizing amorphous layered nanomaterials.^331^ This process fundamentally involves three precisely controlled stages: gradual heating to a predetermined temperature, maintenance at this temperature for a specified duration, and subsequent cooling at a regulated rate (typically employing slow cooling protocols, with the possibility of implementing controlled cooling strategies when necessary).^332^ Paolucci et al. successfully synthesized amorphous *a*-SnO_2_ 2D flakes by annealing SnSe_2_ in air.^333^ Briefly, SnSe_2_ crystals were exfoliated by sonication-assisted liquid phase exfoliation and then annealed in air at 250 °C (below the crystallization temperature of SnO_2_, *T* < 280 °C) for 2 weeks. Compared with crystalline SnSe_2_, amorphous *a*-SnO_2_ had more adsorption sites for gas sensing. Similarly, Zhang et al. proposed a simple phosphorization strategy to prepare amorphous CoFeP nanosheets derived from crystalline CoFe-LDHs.^334^ The CoFe-LDHs precursor was phosphated through the thermal decomposition of NaH_2_PO_2_ under Ar flow. In detail, CoFe-LDHs precursor and NaH_2_PO_2_ were placed in two porcelain boats and transferred to a tube furnace with NaH_2_PO_2_ at the upstream side, followed by annealing up to 310 °C for 2 h under Ar flow. The cystalline-to-amorphous phase transformation from LDHs to CoFeP led to more exposed active sites and faster electron transfer.

Recently, Li et al. successfully prepared O-doped crystalline/amorphous g-C_3_N_4_ nanohomojunction through high-temperature annealing.^335^ Typically, the mixture of melamine, cyanuric acid and KCl was calcined in a tube furnace at 550 °C for a certain time to obtain O-doped crystalline/amorphous g-C_3_N. The crystalline/amorphous g-C_3_N_4_ nanohomojunction well integrated the advantages of amorphous g-C_3_N_4_ and crystalline g-C_3_N_4_, exhibiting exceptional photogenerated carrier separation efficiency and a high density of reactive sites. It should be emphasized that the annealing process is inherently energy-intensive and time-consuming, presenting significant challenges in terms of energy efficiency and process duration optimization.

#### 4.5.4 Other Strategies

In addition to supercritical CO_2_ synthesis, etching treatment and annealing, other strategies such as template-assisted method, hydrothermal synthesis, *n*-BuLi treatment, etc., have also been demonstrated to prepare amorphous layered nanomaterials. Jia et al. developed a universal strategy for synthesizing a range of 2D amorphous metal oxide nanosheets (e.g., Al_2_O_3_, SnO_2_, ZrO_2_, Cr_2_O_3_, and Fe_2_O_3_) using the lamellar Cu_2_O-oleate as a confined host template (Figure 12a).^336^ In the presence of strong acid weak alkali salts such as AlCl_3_, SnCl_4_, ZrCl_4_, CrCl_3_, or FeCl_3_, a slow ion exchange reaction occurred between metal ions and Cu_2_O. The resulting metal hydroxide precursor was confined within the interlayers of oleate to ensure the ultrathin sheet structure. After thermal treatment, the M­(OH)_*x*_-oleate complex intermediates were transformed into amorphous metal oxide nanosheets. This facial method establishes a novel pathway for constructing promising amorphous layered metal oxide nanosheets for biomedical applications.

**Figure 12:**
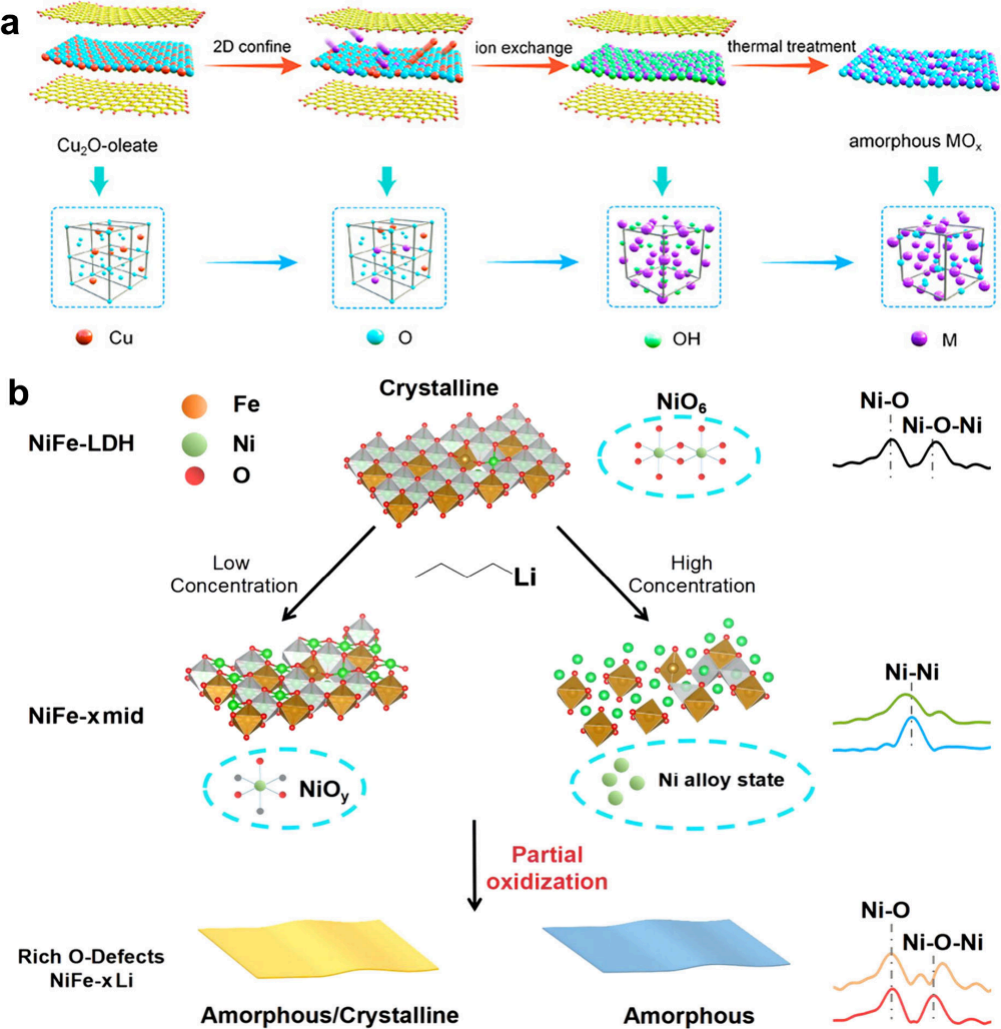
(a) Schematic illustration of preparation of amorphous metal oxide ultrathin nanosheets via template-assisted method. Reproduced with permission from ref 336. Copyright 2020 American Chemical Society. (b) Schematic diagram of the synthesis of NiFe-LDHs by *n*-BuLi treatment. Reproduced with permission from ref 338. Copyright 2023 American Chemical Society.

Xue et al. reported the synthesis of amorphous Ti_3_C_2_ MXene QDs (MQD) using a hydrothermal method with temperature regulation.^337^ The Ti_3_C_2_ MXene powder was mixed with ammonia to adjust the pH to 9 and then was heated in an autoclave at 100 °C, 120 and 150 °C (denoted as MQD-100, MQD-120 and MQD-150) for 6 h, respectively. It was found that MQD-150 displayed an amorphous structure with most of the Ti atoms etched away. Chen et al. proposed an *n*-BuLi treatment method to induce the amorphization of NiFe-LDHs (Figure 12b).^338^ NiFe-LDH powder was treated with *n*-BuLi/hexane at 45 °C under an Ar atmosphere for 48 h and then sonicated in ethanol for 1 h to obtain amorphous NiFe-LDHs. The Ni in NiFe-LDHs was selectively reduced to the alloy state during *n*-BuLi treatment, while Fe remained unchanged. The distinct structural transformations of Ni and Fe effectively induced the formation of OV-rich amorphous/crystalline structure.

#### 4.5.5 Summary

Crystalline-to-amorphous phase transformation strategies have garnered significant attention for fabricating layered nanomaterials with amorphous phase or crystalline–amorphous heterophase. Nevertheless, the inherent high entropy arising from unsaturated bonds and disordered atomic arrangements typically renders amorphous nanomaterials metastable, predisposing them to revert to crystalline states under external stimuli such as pressure or thermal stress. To date, the synthesis of amorphous nanostructures has been confined to a narrow spectrum of nanomaterials, primarily limited to metal oxides and hydroxides. This constraint underscores the critical need for advancing crystalline-to-amorphous phase transformation strategies. Recent studies have demonstrated that amorphous layered nanomaterials can be synthesized through the transformation of crystalline precursors using external forces such as lithiation, pressure, and electricity, which offer precise control over material crystallinity. Moreover, the potential of amorphous nanomaterials in biomedical applications remains largely unexplored. Comprehensive investigations are essential to elucidate how crystallinity (crystalline or amorphous phase) influences the properties of layered nanomaterials in biomedical contexts, thereby unlocking their full potential in this field.

## 5 Characterization

When there is rapid improvement in the breakthrough of technology, several advanced and effective characterization techniques have been well developed and/or identified for analyzing layered nanomaterials, enabling the elucidation of structural changes caused by structural engineering. Particularly, layered nanomaterials subjected to different structural engineering strategies exhibit varying structural characteristics. Precise and detailed characterization of layered nanomaterials is of great significance for clarifying the growth mechanism of material synthesis, understanding the structure–activity relationship between structural characteristics and performance or functions, and facilitating the rational design of layered nanomaterials with tailored structural properties for specific applications. To date, a range of advanced characterization techniques, such as XRD, TEM/STEM, XPS, ESR, AFM, XAFS, Raman spectroscopy, and NMR spectroscopy, have been extensively applied for characterizing the structural changes of layered nanomaterials at the atomic level. In this section, we aim to systematically summarize the well-developed techniques that have been used for structural characterization of layered nanomaterials induced by structural engineering. Moreover, the advantages and limitations of each technique are highlighted and compared.

### 5.1 Powder X-ray Diffraction

XRD has emerged as a powerful nondestructive characterization technique, serving as an essential analytical tool in materials science.^339^ This technique, based on the constructive interference of monochromatic X-rays diffracted by the periodic arrangement of atomic planes in crystalline materials, enables comprehensive structural analysis of both bulk nanomaterials and thin films.^340^ XRD provides critical structural information including interlayer spacing, crystallographic orientation, symmetry elements, and layer stacking sequences, while also facilitating phase identification and detection of secondary phases or impurities.^341^ Furthermore, its exceptional sensitivity to structural modifications makes XRD particularly valuable for investigating perturbation-induced structural changes.^342^ As a cornerstone technique in advanced materials research, XRD has been extensively employed to monitor and characterize structural evolution during various engineering processes, including crystal phase engineering, defect engineering, interlayer engineering, and crystalline-to-amorphous phase engineering, as discussed in previous sections.

Basu et al. proposed an atomic doping strategy for stable conversion of the 2H-phase MoS_2_ into the 1T-phase.^167^ By adjusting the molar ratios of Mo/S (1:6, 1:10), a series of N-doped 1T@2H MoS_2_ (M6, M10) with varied degrees of defects could be obtained. According to XRD spectra (Figure 13a), M6 and M10 displayed downshifted (002) plane diffraction peaks at 13.46° and 13.58° in comparison with the 2H bulk MoS_2_ (M0) at 14.4°. The lattice parameter *a* calculated from the (100) plane diffraction peak was 3.18 Å and 3.19 Å for M6 and M10, respectively, confirming M6 as the semiconducting 2H-phase and M10 as the metallic 1T-phase. Shen et al. adopted XRD to explore the structure changes of CoMo-LDH and NiMo-LDH induced by acid etching-mediated defect engineering.^56^ The pristine CoMo-LDH and NiMo-LDH nanosheets showed typical (003) and (006) diffraction peaks, which were accurately indexed using the standard reference (JCPDS No. 46–0605), indicating their crystal structure. After acid etching, the diffraction peak intensities of defect-rich CoMo-LDH (DR-CoMo-LDH) and defect-rich NiMo-LDH (DR-NiMo-LDH) were obviously lower than that of pristine CoMo-LDH and NiMo-LDH nanosheets, demonstrating a significant decrease in crystallinity of LDH nanosheets after acid etching.

**Figure 13:**
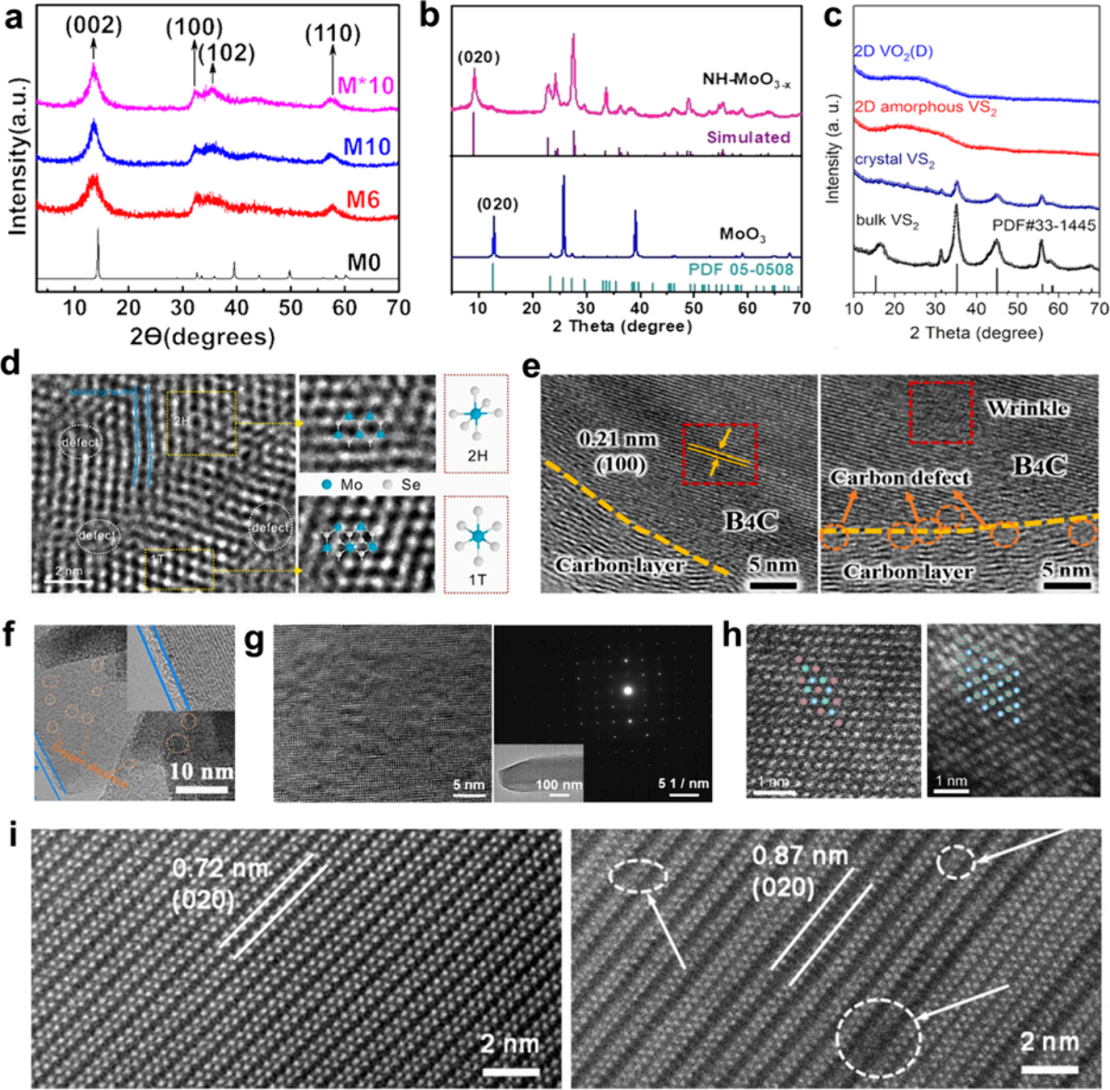
(a) XRD patterns of N-doped 1T@2H MoS_2_ samples. Reproduced with permission from ref 167. Copyright 2019 American Chemical Society. (b) XRD patterns of MoO_3_ and NH-MoO_3*–x*_ nanobelts. Reproduced with permission from ref 53. Copyright 2022 John Wiley and Sons. (c) XRD patterns of bulk VS_2_, crystal VS_2_, 2D amorphous VS_2_, and 2D VO_2_(D). Reproduced with permission from ref 326. Copyright 2020 John Wiley and Sons. (d) HRTEM image of 2H- and 1T-phase coexisted MoSe_2_ nanosheets. Reproduced with permission from ref 175. Copyright 2024 Elsevier. (e) HRTEM images of B_4_C@C. Reproduced with permission from ref 227. Copyright 2021 American Chemical Society. (f) HRTEM image of WO_3*–x*_/C. Reproduced with permission from ref 349. Copyright 2020 American Chemical Society. (g) HRTEM image and SAED pattern of MoO_3_ nanobelts. Reproduced with permission from ref 63. Copyright 2022 John Wiley and Sons. (h) Atomic-resolution HAADF-STEM images of Ti_3_C_2_ and Ti_3_C_2_–SD­(Ti^3+^). Reproduced with permission from ref 208. Copyright 2023 John Wiley and Sons. (i) Atomic-resolution HAADF-STEM images of MoO_3_ and NH-MoO_3*–x*_. Reproduced with permission from ref 53. Copyright 2022 John Wiley and Sons.

Apart from analyzing the structural changes of layered nanomaterials induced by crystal phase engineering and defect engineering, XRD has also demonstrated remarkable capability in elucidating interlayer engineering-mediated structural changes, providing crucial insights into the interlayer spacing, stacking patterns and overall structural integrity of engineered layered nanomaterials. Yasmeen et al. constructed Cl-intercalated porous g-C_3_N_4_ and analyzed the structural changes before and after intercalation using XRD.^307^ The pristine g-C_3_N_4_ showed typical diffraction peaks at 12.9° and 27.3° assignable to the interlayer structural packing and interplanar stacking of the aromatic rings, respectively. After Cl intercalation, the peak intensity caused by the interlayer structural packing decreased slightly, which was proportional to the amount of intercalated Cl. This demonstrated that Cl atoms were effectively intercalated between g-C_3_N_4_ layers, which was conducive to improving charge conductivity and separation for enhanced photocatalytic activity. Similarly, Liu et al. characterized the crystal structure of a series of ion-intercalated g-C_3_N_4_ samples (K^+^-intercalated g-C_3_N_4_ (CN-K), Cl^–^-intercalated g-C_3_N_4_ (CN-Cl), I^–^-intercalated g-C_3_N_4_ (CN-I), K^+^/Cl^–^-intercalated g-C_3_N_4_ (CN-KCl), K^+^/I^–^-intercalated g-C_3_N_4_ (CN-KI), K^+^/I^–^-intercalated g-C_3_N_4_ (CN-KCl/KI)) via XRD.^308^ It was found that a characteristic peak at 27.6° (002) was designated as the stacking of conjugated aromatic systems, while the extremely weak peak at 13.0° (100) was assigned to the in-plane ordered tri-s-triazine units, indicating the formation g-C_3_N_4_ in all samples. Notably, the (002) characteristic peak of K^+^-intercalated g-C_3_N_4_ samples (CN-K, CN-KCl, CN-KI, and CN-KCl/KI) shifted to higher angles in comparison with CN, CN-Cl, and CN-I samples, which may be due to the intercalation of K^+^ reducing the interlayer distance and enhancing the interlayer interaction of g-C_3_N_4_.

Zhou et al. prepared H_2_O/Na^+^ cointercalated NH-MoO_3*–x*_ nanobelts by the interlayer engineering of layered MoO_3_ nanobelts.^53^ The XRD pattern of the MoO_3_ nanobelts aligns well with the standard reference for orthorhombic MoO_3_ (α-MoO_3_, PDF 05-0508), suggesting its crystal structure. As for the NH-MoO_3*–x*_ sample, all diffraction peaks in its XRD pattern closely align with the simulated peaks of hydrated molybdenum bronze [Na­(H_2_O)_2_]_0.25_MoO_3_ that can be considered as layered α-MoO_3_ cointercalated with H_2_O and Na^+^. Moreover, the peak assigned to the (020) planes of the NH-MoO_3*–x*_ nanobelts displays a significant shift to a lower angle compared with that of the MoO_3_ nanobelts (Figure 13b), indicating the expansion of its interlayer spacing. Wu et al. prepared Fe­(II)-Ti_3_C_2_ by anchoring Fe^2+^ into the layer of Ti_3_C_2_ nanosheets through interlayer electrostatic adsorption.^300^ The XRD pattern of Ti_3_C_2_ nanosheets displayed a (002) peak at 6.12°, representing the interlamellar spacing, which shifted to 5.62° in Fe­(II)-Ti_3_C_2_, suggesting an increase in interlayer spacing after anchoring Fe^2+^ into the Ti_3_C_2_ nanosheets. Moreover, the peak intensity of Fe­(II)-Ti_3_C_2_ was significantly higher than that of Ti_3_C_2_ nanosheets, demonstrating its higher crystallinity.

Furthermore, in addition to interlayer engineering, the structural changes induced by crystalline-to-amorphous phase engineering have also been investigated using XRD analysis. Zhou et al. fabricated partial crystallized VO_2_(D) nanosheets and amorphous VS_2_ nanosheets from bulk VS_2_ through supercritical CO_2_ treatment.^326^ The XRD pattern of bulk VS_2_ displayed the characteristic diffraction peaks indexed to hexagonal 2H-VS_2_ bulk material (PDF 33-1445), while all peaks in the XRD pattern of crystal VS_2_ prepared by ultrasonic treatment of bulk VS_2_ were weakened due to the destructive 3D structure (Figure 13c). After supercritical CO_2_ treatment, all diffraction peaks in the XRD patterns of VO_2_(D) and amorphous VS_2_ nanosheets disappeared, ascertaining their amorphous feature. Similarly, Hu et al. successfully fabricated ultrathin amorphous CoW-LDH and NiW-LDH nanosheets through etching strategy.^57^ The XRD patterns of the pristine CoW-LDH and NiW-LDH nanosheets showed typical (003), (006), and (012) diffraction peaks, demonstrating their crystal structure. Whereas no detectable peaks were observed in the XRD patterns of the amorphous CoW-LDH and NiW-LDH nanosheets, indicating their amorphous structure.

In summary, XRD has proven to be an indispensable technique for the structural characterization of advanced layered nanomaterials, demonstrating exceptional capabilities in studying the course of crystal phase engineering, defect engineering, interlayer engineering, and crystalline-to-amorphous phase engineering. The key advantages of XRD technology include: (1) Nondestructive testing: XRD is a noninvasive method, making it ideal for analyzing precious or delicate samples without causing damage. (2) High resolution: XRD delivers high-precision measurements, enabling detailed structural analysis. (3) Rapid analysis: Compared to traditional wet chemistry methods, XRD offers significantly faster analysis speeds. (4) Rich information output: XRD is able to provide a large amount of information about crystal integrity and structure. (5) Broad applicability: XRD is versatile technique applicable to a broad spectrum of materials, including bulk solids and powdered samples. Additionally, XRD combined with Scherrer’s Equation, serves as a fundamental and robust tool for the rapid, nondestructive, and cost-effective determination of average crystallite size in nanomaterials (typically within the range of 1 ∼ 200 nm). Scherrer’s Equation, a classical and widely used method, employs the broadening of diffraction peaks in XRD patterns to estimate the average crystallite size (or coherent scattering domain size) perpendicular to the diffracting crystal planes in polycrystalline or nanocrystalline materials.^343,344^ Known for its excellent statistical representativeness and convenience, this technique is crucial for exploring variations in crystallite size within structurally engineered layered nanomaterials. It also plays an indispensable role in the fields of materials science research, synthesis process development, and industrial quality control.

Whereas its drawbacks involve: (1) Limited ability to identify amorphous materials: XRD is primarily designed for crystalline materials, and it is difficult to provide effective information for amorphous materials or poorly ordered structures. (2) Stringent sample requirements: High-quality samples with well-defined crystal structures are necessary for accurate analysis, as low crystallinity or irregular samples may yield unreliable results. (3) Surface-limited information: XRD typically provides information about the sample’s surface and near-surface regions, with limited ability to probe deeper internal structures. (4) Complex and costly equipment: XRD instruments are sophisticated and expensive, and data interpretation requires specialized knowledge and expertise. (5) Limited chemical and molecular insights: While XRD excels in crystallographic analysis, it offers relatively limited information about the chemical composition and molecular structure of materials. In conclusion, XRD is a powerful tool for structural characterization, but its application must be carefully considered in light of its limitations, particularly when dealing with noncrystalline or complex materials.

### 5.2 Transmission Electron Microscopy

Transmission electron microscopy technology plays an important role in materials science, with three common modes including conventional TEM, HRTEM, and STEM. Each mode has its unique advantages and applicable scenarios.

TEM, as a useful technique for characterizing the phase, crystallinity, size, exposed surface, and growth direction of materials, has been widely employed to characterize the structural changes of layered nanomaterials caused by structural engineering.^345,346^ A TEM image is generated by collecting the interaction signals of electrons when transmitted from the sample at a high accelerating voltage of 60–300 kV (200 kV is commonly used). It should be noted that TEM electron irradiation may cause structure changes or destruction in ultrathin nanomaterials, which can be mitigated by using a low accelerating voltage (e.g., 80 kV) during TEM measurement.^66^ Low-magnification TEM can be utilized to accurately measure the lateral size of layered nanomaterials and roughly evaluate their thickness based on the contrast of TEM images.^347^ For example, Wu et al. synthesized Fe­(II)-Ti_3_C_2_ through interlayer engineering by anchoring Fe^2+^ into the layer of ultrathin Ti_3_C_2_ nanosheets, and compared the thickness of Ti_3_C_2_ and Fe­(II)-Ti_3_C_2_ using TEM.^300^ According to TEM images, Ti_3_C_2_ exhibited a clear lattice fringe of 2.89 nm at the edge of nanosheets, whereas Fe­(II)-Ti_3_C_2_ showed that of 3.42 nm, which is consistent with the thickness determined by AFM.

Unlike low-magnification TEM, HRTEM paired with selected area electron diffraction (SAED) patterns can examine the exposed crystal facets, crystal orientation, crystallinity, and phase of layered nanomaterials.^347^ For crystalline layered nanomaterials, the exposed crystal facets can be identified by analyzing the lattice spacing in HRTEM images, and the corresponding crystal phases of layered nanomaterials can be analyzed. Xia et al. prepared 2H- and 1T-phase coexisted MoSe_2_ nanosheets through Co doping-mediated crystal phase engineering.^175^ HRTEM images showed two different atomic arrangements: the octahedral (1T) phase and the trigonal prismatic (2H) phase in a typical Co-MoSe_2_ nanosheet (Figure 13d). Moreover, abundant defects and stacking faults were found. These results indicated that the introduction of Co into MoSe_2_ nanosheets not only facilitated the phase transformation from 2H-phase to 1T-phase, but also promoted the generation of defects. Mutalik et al. prepared 1T- and 2H-MoS_2_ nanosheets through solvothermal method-mediated crystal phase engineering and characterized their crystal phases using HRTEM.^176^ The results indicated that 1T-MoS_2_ nanosheets possessed a larger interplanar spacing of 0.95 nm assignable to (002) plane in comparison with 2H-MoS_2_ nanosheets with an interlayer distance of 0.62 nm. Wang et al. fabricated an intelligent drug delivery system (Au@LDH/B) via interlayer engineering by intercalating butyrate into NiTi-LDHs film and loading GNR.^348^ The crystal phase of the film (the contact area between GNRs and NiTi-LDHs) could be transformed from LDHs to LDOs under NIR (808 nm) irradiation. Before NIR irradiation, the characteristic fringes (0.275 nm attributed to (009) plane) corresponding to NiTi-LDHs could be found in HRTEM image, no matter away from (green square) or in the area near (red square) GNRs. After NIR irradiation, the characteristic fringes (0.374 nm attributed to (006) plane) associated with NiTi-LDHs could only be found away from GNRs, while regions proximate to GNRs exhibited a phase transition to NiTi-LDOs (0.211 nm attributed to (200) plane). When NIR irradiation was stopped, the crystal phase of LDOs turned back to LDHs in PBS or dodecanoic (DCA) sodium solution by absorbing phosphate anion or DCA ions. As revealed by HRTEM image, only characteristic fringes (0.261 nm attributed to (100) plane or 0.512 nm attributed to (006) plane) of NiTi-LDHs could be found while lattices corresponding to NiTi-LDOs disappeared.

The normal HRTEM mode can also identify vacancies and defects in nanosheets. Guo et al. constructed carbon defect-enriched B_4_C@C nanosheets through hydrothermal method-mediated defect engineering, whose HRTEM image showed that there was a carbon layer in the epitaxial region of B_4_C surface, and obvious distortion accompanied by abundant carbon defects was observed at the interface region (Figure 13e).^227^ Zhang et al. synthesized tungsten trioxide with coexisting OVs and carbon coating (WO_3*–x*_/C) through defect engineering by using a one-pot carbon-coating method.^349^ From the edge of the HRTEM image of WO_3*–x*_/C, an irregular substance approximately 2–3 nm thick (blue circumferential region) was observed, corresponding to the carbon coating on the WO_3*–x*_ surface. Moreover, the lattice fringes in the HRTEM image are unclear, with scattered lattice distortions throughout the entire region (Figure 13f), demonstrating the presence of defects.

In terms of crystallinity, single-crystalline layered nanomaterials exhibit continuous lattice fringes with uniform orientation in HRTEM images and distinct bright diffraction spots in SAED pattern images. Polycrystalline layered nanomaterials show discontinuous lattice domains with random orientations in HRTEM images and ring-like SAED patterns, while neither SAED patterns nor lattice fringes are observed in amorphous layered nanomaterials.^66^ For example, Sun et al. prepared oxygen-poor vacancies BBP and oxygen-rich vacancies BBR through chemical reduction-mediated defect engineering.^218^ The SAED pattern of BBP sample displayed obvious regular square diffraction spots, revealing its single-crystal structure, while the SAED pattern of BBR sample showed multiple diffraction rings, indicating its polycrystalline structure. Hu et al. used HRTEM and SAED pattern to characterize the crystallinity of MoO_3_ nanobelts before interlayer engineering.^63^ As shown in Figure 13g, the continuous crystal lattice fringes and the rectangular-shaped bright diffraction spots can be found in HRTEM image and SAED pattern of a typical MoO_3_ nanobelt, demonstrating its single-crystalline nature. Liu et al. compared the structural characteristics of crystal MoO_3_ nanosheets and amorphous MoO_3_ nanosheets prepared by crystalline-to-amorphous phase engineering using HRTEM and SAED pattern.^325^ Intermittent lattice fringes were observed in HRTEM image of crystal MoO_3_ nanosheet, while strongly disordered atomic arranging manners on the basal surface were found in HRTEM image of amorphous MoO_3_ nanosheet. The corresponding SAED pattern of amorphous MoO_3_ nanosheet displayed a closed diffraction ring, confirming its amorphous nature.

STEM can serve as a powerful technique for imaging individual atoms, especially after introducing aberration-corrected optics.^350–353^ Similar to SEM, the focused electrons in STEM mode are scanned instead of transferred to the sample in a raster. Atomic resolution images can be acquired using high-angle annular dark-field scanning transmission electron microscope (HAADF-STEM), where atomic contrast scales with atomic number.^354^ HAADF-STEM technique can not only precisely identify atomic positions in layered nanomaterials for quantitative determination of atomic ratio, but also distinguish their different crystal phases and layer spacing. For example, Zhou et al. utilized HAADF-STEM to identify the 1T-phase domain in single-layer MoS_2_ nanodots prepared by intercalation-induced crystal phase engineering.^59^ MoS_2_ nanodots display clear atomic arrangements with a size of 5 nm. The filtered HAADF-STEM image clearly shows the triangular shape lattice fringes of Mo atoms, supporting the metallic 1T-phase structure of the as-prepared MoS_2_ nanodots.

Mao et al. employed HAADF-STEM to analyze the Ti_3_C_2_ and Ti_3_C_2_[Ti_3_C_2_–SD­(Ti^3+^)] nanosheets prepared by defect engineering.^208^ The results indicated that Ti atoms were arranged in a regular parallelogram form in the Ti_3_C_2_ nanosheets, while in a regular rectangle form in the Ti_3_C_2_[Ti_3_C_2_–SD­(Ti^3+^)] nanosheets (Figure 13h), which could be attributed to the planar shift of Ti atoms positioned in the second and third layers relative to the first layer, resulting in 2D catalytic planar defects. Geng et al. characterized Ti_3*–x*_C_2_T_*y*_ and Pd_SA_/Ti_3*–x*_C_2_T_*y*_ nanosheets engineered by heteroatom doping via HAADF-STEM, from which Ti vacancies were observed in the Ti_3*–x*_C_2_T_*y*_ sample and dispersive small bright dots of Pd single atoms were found in the crystal lattice of the Pd_SA_/Ti_3*–x*_C_2_T_*y*_ sample.^299^ Zhou et al. compared the structural characteristics of MoO_3_ and H_2_O/Na^+^ cointercalated NH-MoO_3*–x*_ nanobelts via atomic-resolution HAADF-STEM.^53^ Continuous lattice fringes can be detected in the atomic-resolution STEM images of MoO_3_ and NH-MoO_3*–x*_ nanobelts, indicating their single-crystalline properties. The measured interlayer spacing assigned to (020) planes of MoO_3_ nanobelts is ∼0.72 nm, while that of NH-MoO_3*–x*_ nanobelts is ∼0.87 nm (Figure 13i), proving the obvious expansion of the interlayer spacing of NH-MoO_3*–x*_ nanobelts after H_2_O/Na^+^ cointercalation. Meanwhile, some defects can be found in the atomic-resolution STEM image, indicating that the intercalation process also generates some defects. Hu et al. also adopted STEM to explore the structure changes of CoW-LDH nanosheets before and after acid etching.^57^ The atomic resolution STEM image of CoW-LDH nanosheets shows several crystalline domains with negligible defects, consistent with their polycrystalline structure. Whereas no distinct lattice fringes were found in that of a-CoW-LDH nanosheets, confirming their successfully crystalline-to-amorphous phase transformation after acid treatment.

Overall, TEM, HRTEM and STEM have demonstrated exceptional capabilities in characterizing structural changes of layered nanomaterials induced by crystal phase engineering, defect engineering, heteroatom doping, interlayer engineering, and crystalline-to-amorphous phase engineering. These advanced microscopy techniques share several key advantages: (1) Superior resolution: TEM offers subnanometer resolution, ideal for examining material contours and dimensions, while HRTEM achieves atomic resolution, enabling detailed visualization of internal crystal structures and atomic arrangements. STEM, utilizing a point-scan methodology, provides even finer resolution than both TEM and HRTEM. (2) Multifunctionality: Beyond imaging, TEM, HRTEM, and STEM can integrate diffraction and harmonic techniques to gather comprehensive data on chemical composition and crystal structure. (3) Broad applicability: These techniques are versatile, applicable to a wide range of materials including inorganic substances, metals, semiconductors, as well as organic and biological specimens (e.g., cells and organelles). They are instrumental in elucidating critical material characteristics such as microstructure, morphology, crystal structure, defects, chemical composition, and electronic properties. (4) High-energy electron beam utilization: The use of high-energy electron beams in TEM, HRTEM, and STEM allows for the revelation of intricate sample details beyond the capabilities of optical microscopy. This includes insights into crystalline phase orientation and elemental composition, with the exception of the lightest elements (hydrogen and helium).

Nevertheless, the operation and image analysis of TEM, HRTEM, and STEM demand a high degree of expertise and extensive experience. Moreover, the requirement for a high vacuum environment can be a constraint for studying unstable or volatile samples. Additionally, the high-energy electron beams may potentially alter the sample’s surface properties or cause damage, thereby impacting the accuracy of observations. While TEM, HRTEM, and STEM are powerful tools for nanomaterial analysis, their effective application requires careful consideration of their operational complexities and potential sample interactions. It is important to recognize that human bias can influence the data acquisition process in microscopic imaging techniques: researchers might overemphasize targeted morphological features while overlooking the overall feature distribution across the sample. Artificial intelligence (AI) tools, particularly those based on deep learning algorithms like Convolutional Neural Networks (CNNs) and Generative Adversarial Networks (GANs), offer a powerful means to counteract these cognitive biases. These AI technologies have the potential to revolutionize structural engineering research in layered nanomaterials by automating the analysis of microscopy data, enabling statistically representative sampling across the entire specimens rather than just human-selected regions.^355^ AI tools are not intended to replace traditional techniques but to enhance them, with the following application prospects: (1) Bias reduction: AI-powered image segmentation (e.g., U-Net architectures) objectively quantifies morphological features (layer thickness, defect density, domain boundaries), eliminating observer-dependent variability.^356^ (2) High-throughput correlation: Multimodal data fusion algorithms integrate microscopy results with XRD and XPS data to establish structure–property relationships inaccessible through manual analysis. Thus, AI tools are poised to transform the structural characterization of layered nanomaterials into a data-driven feedback loop for precision biomaterial design.

### 5.3 X-ray Photoelectron Spectroscopy

As a highly sensitive spectroscopic technique, XPS has been extensively employed for characterizing the elemental composition of layered nanomaterials, which represents the binding energy of core-level electrons emitted from a material as a function of their number when the material is exposed to the X-ray beam.^357^ The characteristic binding energy peaks corresponding to specific electronic states of elements enable precise identification of elemental composition and chemical states.^358,359^ Notably, XPS has emerged as a pivotal analytical tool for monitoring compositional variations in layered nanomaterials tuned by various structural engineering strategies, including but not limited to crystal phase engineering, defect engineering, interlayer engineering, and crystalline-to-amorphous phase engineering.

Layered nanomaterials with distinct crystal phases can be identified and differentiated through XPS. Xia et al. prepared 2H- and 1T-phase coexisted MoSe_2_ nanosheets through Co doping-mediated crystal phase engineering (CM0, CM5, CM10 and CM20 are assigned to the Co/Mo atomic ratio of 0%, 5%, 10% and 20%, respectively).^175^ In the high-resolution XPS spectra of Co 2p (Figure 14a), the binding energies gradually decrease as Co doping increases, which may be attributed to the electron flowing to Mo, resulting in a change in the electron density of Co. In the Mo 3d XPS spectra (Figure 14b), the peaks at 232.3 eV (Mo^4+^ 3d_3/2_) and 229.3 eV (Mo^4+^ 3d_5/2_) of the undoped MoSe_2_ nanosheets (CM0) are attributable to the characteristic peaks of 2H-phase MoSe_2_. After Co doping, the binding energies in the Mo 3d spectra of MoSe_2_ nanosheets with different Co doping amounts change and shift obviously. Typically, the Mo^4+^ 3d_5/2_ and 3d_3/2_ main peaks of CM20 can be deconvolved into 227.8 eV (1T-phase)/228.8 eV (2H-phase) and 230.9 eV (1T-phase)/231.9 eV (2H-phase), respectively. Similarly, two peaks at 55.5 and 54.8 eV correspond to the 2H-phase are observed in the Se 3d XPS spectra of CM0 (Figure 14c), while two deconvolution peaks at 54.99 and 53.9 eV belong to the 1T-phase are found in the Se 3d spectra of CM5. Notably, the Se 3d spectra show a significant peak shift to lower binding energies with the increase of Co doping, which is due to the generation of V_Se_ caused by Co doping.

**Figure 14:**
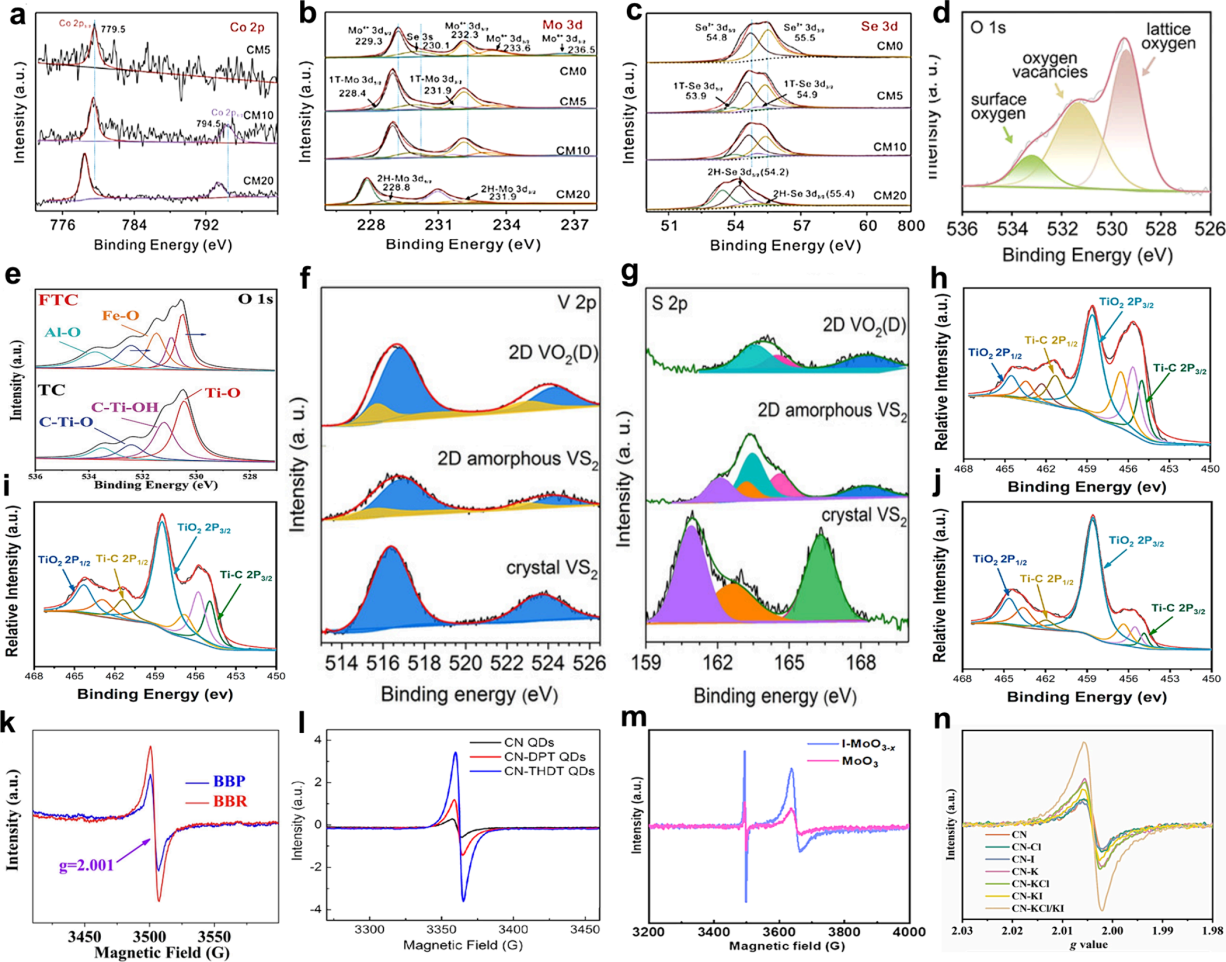
XPS spectra of (a) Co 2p, (b) Mo 3d and (c) Se 3d of CM0, CM5, CM10, and CM20. Reproduced with permission from ref 175. Copyright 2024 Elsevier. (d) High-resolution XPS O 1s spectra of BiO_2*–x*_ nanosheets. Reproduced with permission from ref 52. Copyright 2023 John Wiley and Sons. (e) High-resolution XPS O 1s spectra of Fe­(II)-Ti_3_C_2_ and Ti_3_C_2_. Reproduced with permission from ref 300. Copyright 2021 John Wiley and Sons. High-resolution XPS spectra of (f) V 2p and (g) S 2p of crystal VS_2_, 2D amorphous VS_2_ and 2D VO_2_(D). Reproduced with permission from ref 326. Copyright 2020 John Wiley and Sons. XPS spectra of Ti 2p of (h) Ti_3_C_2_ nanosheets, (i) H_L_–Ti_3_C_2_ nanosheets and (j) H_H_–Ti_3_C_2_ nanosheets. Reproduced with permission from ref 244. Copyright 2022 Elsevier. (k) ESR spectra of BBP and BBR samples. Reproduced with permission from ref 218. Copyright 2022 Elsevier. (l) ESR spectra of three engineered g-C_3_N_4_ QDs samples. Reproduced with permission from ref 242. Copyright 2019 American Chemical Society. (m) ESR spectra of MoO_3_ and I-MoO_3*–x*_. Reproduced with permission from ref 61. Copyright 2023 John Wiley and Sons. (n) ESR spectra of ion-intercalated g-C_3_N_4_ samples. Reproduced with permission from ref 308. Copyright 2023 National Academy of Sciences.

In addition to distinguishing crystal phases, XPS can also be utilized for qualitative analysis of elemental compositions of layered nanomaterials tuned by defect engineering, interlayer engineering and crystalline-to-amorphous phase engineering. For example, Yang et al. prepared BiO_2*–x*_ nanoparticles by hydrothermal method and further synthesized oxygen-defect-rich BiO_2*–x*_ nanosheets through ultrasonic fragmentation treatment-mediated defect engineering.^52^ In the high-resolution XPS spectra of O 1*s*, three main peaks at 529.83, 530.92, and 532.88 eV derived from lattice oxygen, OV, and surface oxygen were observed in both BiO_2*–x*_ nanoparticles and BiO_2*–x*_ nanosheets. Notably, the peak intensity of OV in BiO_2*–x*_ nanosheets was significantly enhanced compared with that in BiO_2*–x*_ nanoparticles (Figure 14d), suggesting an increase in OV concentration. Wu et al. prepared Fe­(II)-Ti_3_C_2_ through interlayer engineering by anchoring Fe^2+^ into the layer of Ti_3_C_2_ nanosheets.^300^ As revealed by high-resolution O 1*s* spectra, there was a peak corresponding to the Fe–O bond at 531.7 eV in the Fe­(II)-Ti_3_C_2_, which was not found in the Ti_3_C_2_ nanosheets (Figure 14e), suggesting that doped Fe^2+^ ions were interacted with the oxygen groups.

Zhou et al. fabricated crystal VS_2_, amorphous VS_2_ and partial crystallized VO_2_(D) nanosheets from bulk VS_2_, and disclosed the changes in the chemical composition of the samples before and after crystalline-to-amorphous phase transformation via XPS.^326^ In the XPS V 2p spectra (Figure 14f), two peaks of crystal VS_2_ located at 516.35 and 523.70 eV are ascribed to V^4+^, while the corresponding peaks of amorphous VS_2_ and VO_2_(D) nanosheets shift to 516.7 and 524.3 eV. Moreover, the banding energies at 515.6 and 523 eV assignable to V^3+^ are discerned in amorphous VS_2_ and VO_2_(D), indicating the partial reduction of V^4+^ induced by crystal phase transformation. In the XPS S 2p spectra (Figure 14g), two banding energies of crystal VS_2_ at 161.10 and 162.50 eV belong to S^2–^, and a peak at 166.40 eV corresponds to S^4+^. Notably, the peaks of S^2–^ in amorphous VS_2_ shift to larger banding energy, demonstrating a low coordination environment of sulfur ion, while the peaks of S^2–^ are unavailable in VO_2_(D), suggesting the presence of pure VO_2_(D) without S-doping. In the XPS O 1s spectra, the V–O bond at 529.60 eV is found in VO_2_(D) due to the production of vanadium oxide, which is unavailable in the crystal VS_2_ or amorphous VS_2_, confirming the successful phase transformation of crystal VS_2_ to VO_2_.

Interestingly, not limited to the qualitative analysis of crystal phases and composition changes, the content of different valence state of elements can be quantitatively determined through XPS by deconvoluting the binding energy peaks and calculating the peak areas. For instance, Li et al. prepared Ti_3_C_2_ nanosheets and further fabricated H_L_–Ti_3_C_2_ and H_H_–Ti_3_C_2_ nanosheets through defect engineering by high-temperature treatment for 1 and 2 h, respectively.^244^ In the XPS Ti 2p spectra (Figure 14h–j), the peaks at 456.52, 463.45, 455.64, 462.29, 455, 461.3, 458.59, and 464.51 eV were assigned to the Ti^3+^ (2p_3/2_), Ti^3+^ (2p_1/2_), Ti^2+^ (2p_3/2_), Ti^2+^ (2p_1/2_), Ti–C (2p_3/2_), Ti–C (2p_1/2_), TiO_2_ (2p_3/2_), and TiO_2_ (2p_1/2_), respectively. It was found that the peak intensity of Ti–C bond decreased while that of the Ti–O bond increased with the high-temperature treatment, as the peak area of the Ti–C bond decreased from ∼19.2% to ∼18.1% (H_L_–Ti_3_C_2_), and further to ∼7.9% (H_H_–Ti_3_C_2_ nanosheets), while those of the Ti–O bond increased from ∼39.6% to ∼51.3%, then to ∼67.7%, respectively, suggesting the oxidation of Ti_3_C_2_ nanosheets to form TiO_*x*_.

Overall, XPS technology has become a pivotal analytical tool for investigating composition changes of layered nanomaterials tuned by crystal phase engineering, defect engineering, heteroatom doping, interlayer engineering, and crystalline-to-amorphous phase engineering. This surface-sensitive technique offers several distinctive advantages: (1) Exceptional sensitivity and microanalytical capability: As an ultrahigh vacuum surface analysis method, XPS exhibits remarkable sensitivity, enabling detection of elements at trace concentrations (typically in the range of 0.1–1.0 atomic percent). (2) Nondestructive characterization: The technique causes minimal sample damage, making it particularly suitable for delicate or beam-sensitive materials. (3) Rapid multielement analysis: XPS facilitates simultaneous qualitative and quantitative analysis of all elements (excluding hydrogen and helium) within a relatively short time frame (typically 5–15 min per sample). (4) Comprehensive chemical state information: The technique provides detailed insights into elemental composition, oxidation states, and chemical bonding environments. However, XPS does present certain limitations, including relatively modest energy resolution (typically 0.3–1.0 eV) and the inability to detect elements with atomic numbers below 11 (sodium), which restricts its application for light element analysis.

### 5.4 Electron Spin Resonance Spectroscopy

ESR spectroscopy is a powerful analytical technique that exploits the fundamental phenomenon of electron spin to detect unpaired electrons in nanomaterials.^360^ This technique has found extensive applications in characterizing paramagnetic species, including free radicals, transition metal ions, and various defect structures in materials, as well as investigating metal ion coordination and active sites in proteins in biomedicine.^361,362^ Owing to its exceptional sensitivity, ESR spectroscopy is particularly effective in detecting subtle magnetic signals, especially those arising from free radicals, paramagnetic transition metal ions, and structural defects in materials.^363,364^ These remarkable capabilities have established ESR as an indispensable tool for characterizing vacancy-type defects in layered nanomaterials engineered through various strategies, such as defect engineering, heteroatom doping, interlayer engineering, and crystalline-to-amorphous phase engineering. The nature and concentration of these defects can be precisely determined through analysis of characteristic g-factors and corresponding signal intensities in the ESR spectra.

Jiang et al. prepared TiO_*x*_@C through defect engineering by exfoliating bulk Ti_3_AlC_2_ with HF and then oxidizing them with H_2_O_2_.^199^ ESR analysis displayed a clear signal of exfoliated Ti_3_C_2_ MXene at g = 1.946, demonstrating the existence of individual Ti vacancies after HF exfoliation. In contrast, a strong ESR signal at g = 2.009 was found in TiO_*x*_@C, which did not match with the reported ESR signals of O_2_^–^ (g = 2.020), Ti^3+^ defects (g = 1.940 to 1.990), or OVs (g = 2.003), indicating that the surface Ti vacancies in TiO_*x*_@C may be located in a new coordination environment due to H_2_O_2_ oxidation. Meanwhile, there was still a relatively weak ESR signal at g = 1.946, suggesting that the Ti^3+^ defects previously detected in the exfoliated Ti_3_C_2_ still existed in the TiO_*x*_@C. Similarly, Sun et al. exploited ESR spectroscopy to characterize the oxygen-poor vacancies BBP and oxygen-rich vacancies BBR prepared by chemical reduction-mediated defect engineering.^218^ The remarkable ESR signal at g = 2.001 was observed in both BBP and BBR samples (Figure 14k), suggesting the presence of OVs. It is worth noting that the ESR signal intensity of BBR sample was significantly higher than that of the BBP sample, implying more OVs generation in BBR via a facile reduction action. Wu et al. synthesized three engineered g-C_3_N_4_ QDs (CN, CN-DPT, and CN-THDT) through defect engineering (calcination and subsequent simple quenching process).^242^ The ESR signal reveals the unpaired electrons on the π-conjugated aromatic rings trapped at defect sites. The increase in signal intensity indicates a stronger ability to capture unpaired electrons on defects to generate photogenerated carriers, which favors ROS generation. Notably, CN-THDT QDs exhibited the strongest catalytic activity, as the strongest ESR signal was observed (Figure 14l).

Aside from characterizing defects in layered nanomaterials tuned by defect engineering, Jana et al. also analyzed the structural changes of Cu-doped MoOx nanozyme prepared by hydrothermal method-mediated heteroatom doping using ESR spectroscopy.^273^ It was found that a strong resonance signal (g = 2.003) attributed to electrons trapped in OVs was observed in Cu-doped MoOx, while a weaker signal was detected on undoped MoOx, demonstrating the generation of OVs during the doping of Cu. Similarly, Yu et al. employed the same method to synthesize Fe-doped MoO_*x*_ nanozyme, which delivered a stronger resonance signal at g = 2.003 compared with undoped MoOx.^274^

In addition to defect engineering and heteroatom doping, Li et al. explored the interlayer engineering-induced structural change of MoO_3_ and I-MoO_3*–x*_ (cointercalated with Na^+^ and H_2_O) nanobelts using ESR spectroscopy, from which two peaks at g = 1.92 and 2.003 originating from Mo^5+^ and OVs were observed (Figure 14m).^61^ As expected, the I-MoO_3*–x*_ nanobelts exhibited stronger intensity than that of the MoO_3_ nanobelts on both peaks, demonstrating the presence of much more Mo^5+^ and OVs. Liu et al. employed ESR spectroscopy to detect the unpaired electrons in a series of ion-intercalated g-C_3_N_4_ samples (CN-K, CN-Cl, CN-I, CN-KCl, CN-KI, CN-KCl/KI) prepared by interlayer engineering.^308^ All samples displayed a Lorentz line at g = 2.004 (Figure 14n), attributing to the unpaired electrons in g-C_3_N_4_. Compared with other samples, CN-KCl/KI demonstrated the highest ESR intensity, demonstrating the most unpaired electrons.

Additionally, Hu et al., Yang et al., and Cao et al. employed ESR spectroscopy to characterize the structural changes of a series of LDHs (e.g., CoW-LDH, Zn-CoMo-LDH, CoBiFe-LDH, etc.) before and after acid etching-mediated crystalline-to-amorphous phase engineering.^57,327,329^ Compared with the pristine CoW-LDH, Zn-CoMo-LDH and CoBiFe-LDH nanosheets without obvious ESR signals, their amorphous phases (a-CoW-LDH, a-Zn-CoMo-LDH and a-CoBiFe-LDH) showed a strong signal at g = 2.2 in the ESR spectra, demonstrating the generation of rich OVs after crystalline-to-amorphous phase engineering.

Several important technical considerations should be noted regarding ESR spectroscopy implementation. The instrumentation requires substantial capital investment due to its sophisticated design and the necessity for cryogenic temperature control systems, typically utilizing liquid helium (4.2 K) or liquid nitrogen (77 K) cooling. Furthermore, ESR measurements demand precise control of experimental parameters, including stable magnetic field strength and temperature conditions, which necessitates specialized infrastructure and rigorous environmental controls. These stringent operational requirements significantly contribute to the complexity of ESR experiments and underscore the need for specialized technical expertise in both instrument operation and data interpretation.

### 5.5 Atomic Force Microscopy

AFM serves as a powerful complementary technique to TEM, operating through the precise measurement of local forces between a nanoscale cantilever tip and the sample surface.^365^ This scanning probe technique enables high-resolution 3D topographic mapping of surfaces with nanometer-scale precision.^366,367^ In the characterization of layered nanomaterials, AFM plays a crucial role in determining material thickness and visualizing nanosheet packing density.^368^ However, for accurate layer number identification, AFM data typically requires correlation with additional characterization methods, including optical microscopy, photoluminescence spectroscopy, and Raman spectroscopy.^369^ The exceptional sensitivity of AFM to surface topography and thickness variations makes it an indispensable tool for monitoring structural changes in layered nanomaterials, particularly in quantifying thickness changes resulting from interlayer engineering processes.

Zhou et al. reported that the MoO_3_ nanobelts have a thickness of 60–130 nm (Figure 15a), while the MoO_3*–x*_ nanobelts obtained by ball milling and lithium intercalation of MoO_3_ have a thickness of 4–13 nm (Figure 15b).^60^ Song et al. compared the thickness of Li-ion-intercalated Ti_3_C_2_T_*x*_ nanosheets with the single-layer Ti_3_C_2_T_*x*_ using AFM.^169^ The results revealed that the thickness of single-layer Ti_3_C_2_T_*x*_ was ∼1.5 nm (Figure 15c) and the Li-ion-intercalated Ti_3_C_2_T_*x*_ nanosheets comprised about 4 ∼ 5 layers with a thickness of ∼6 nm (Figure 15d), validating the few-layer structure of Li-ion-intercalated Ti_3_C_2_T_*x*_. Wu et al. synthesized Fe­(II)-Ti_3_C_2_ through interlayer engineering by anchoring Fe^2+^ into the layer of ultrathin Ti_3_C_2_ nanosheets through interlayer electrostatic adsorption.^300^ The thickness of Ti_3_C_2_ determined by AFM was 2.91 nm, equivalent to five monatomic layers thickness, while Fe­(II)-Ti_3_C_2_ showed a thickness of 3.42 nm, indicating that the doping of Fe^2+^ caused an increased two-layer *d*-spacing. Guo et al. reported that the nitroprusside-intercalated MgMnFe-LDH showed an increase in thickness (3–4 nm) compared with pristine MgMnFe-LDH (∼1 nm).^305^ Similarly, Ning et al. found that the thickness of NiAl-LDH detected by AFM increased from ∼2 nm to ∼20 nm after intercalation of Fe-TCPP.^312^

**Figure 15:**
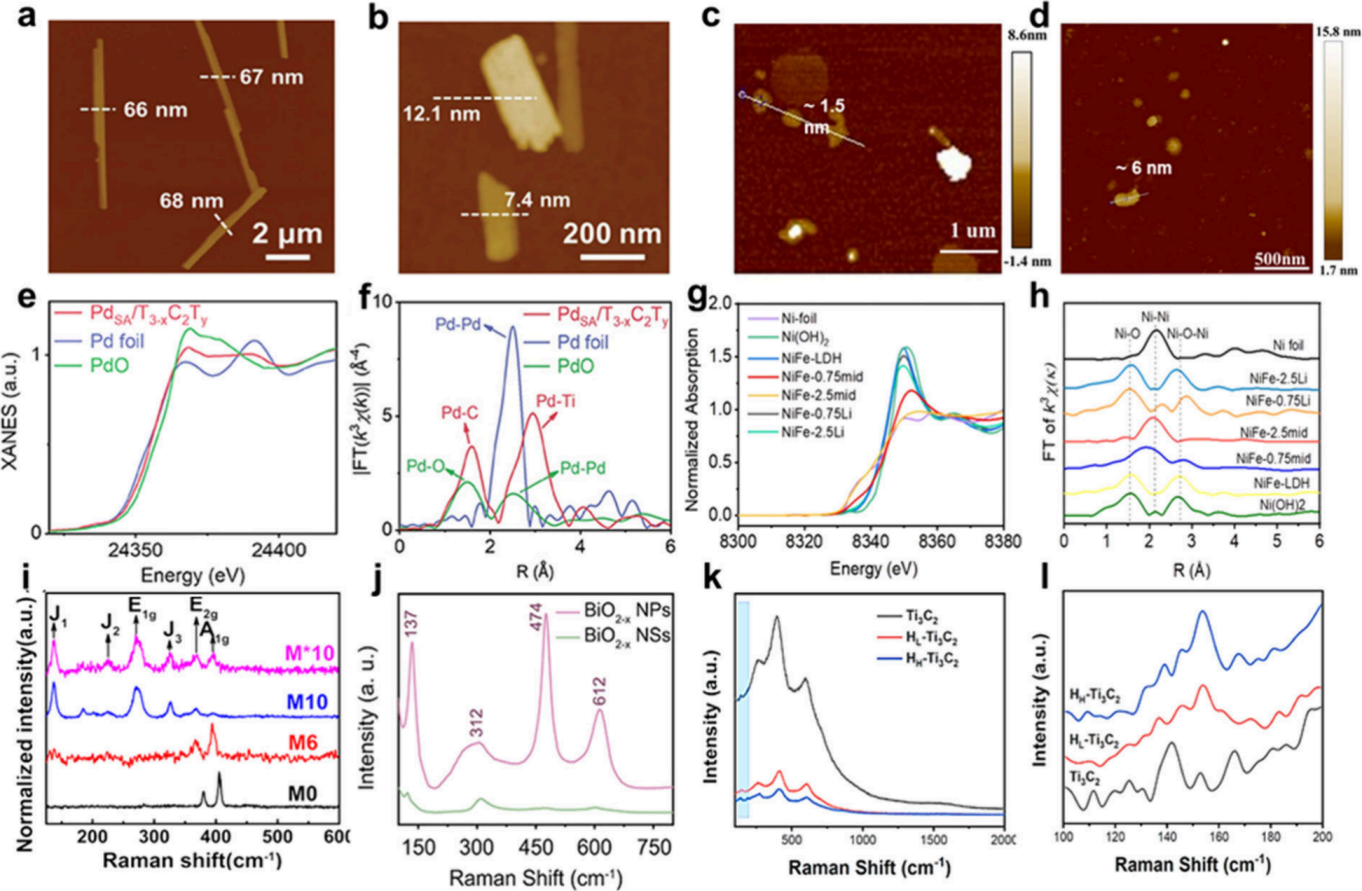
AFM images of (a) MoO_3_ nanobelts and (b) MoO_3*–x*_ nanobelts. Reproduced with permission from ref 60. Copyright 2021 John Wiley and Sons. AFM images of (c) single-layer Ti_3_C_2_T_*x*_ and (d) Li-ion-intercalated Ti_3_C_2_T_*x*_ nanosheets. Reproduced with permission from ref 169. Copyright 2023 Elsevier. (e) Pd K-edge XANES spectra and (f) Fourier-transformed extended EXAFS of Pd_SA_/Ti_3*–x*_C_2_T_*y*_, Pd foil and PdO. Reproduced with permission from ref 299. Copyright 2024 John Wiley and Sons. (g) Ni K-edge XANES and (h) FT plots of the EXAFS k^3^ χ­(k) function in the R-space of Ni foil, NiFe-2.5Li, NiFe-0.75Li, NiFe-2.5mid, NiFe-0.75mid, NiFe-LDH, and Ni­(OH)_2_. Reproduced with permission from ref 338. Copyright 2023 American Chemical Society. (i) Raman spectra of N-doped 1T@2H MoS_2_ sample. Reproduced with permission from ref 167. Copyright 2019 American Chemical Society. (j) Raman spectra of BiO_2–*x*_ nanosheets and BiO_2–*x*_ nanoparticles. Reproduced with permission from ref 52. Copyright 2023 John Wiley and Sons. (k) Raman spectra of Ti_3_C_2_ nanosheets, H_L_–Ti_3_C_2_ nanosheets and H_H_–Ti_3_C_2_ nanosheets and (l) corresponding magnification in the blue area. Reproduced with permission from ref 244. Copyright 2022 Elsevier.

Special consideration must be given when characterizing layered nanomaterials using AFM, as thickness measurements can be significantly influenced by several factors. These include the substantial chemical contrast between the nanomaterials and their supporting substrates, surface-adsorbed contaminants, and the presence of interfacial water layers trapped beneath the nanomaterials. Furthermore, solution-processed layered nanomaterials often exhibit inflated AFM height measurements compared to their theoretical thickness values, primarily due to residual ligand coatings, nonvolatile solvent molecules, and increased surface roughness. Despite these measurement challenges, AFM offers several notable advantages, including atomic-scale resolution, straightforward operation, and broad applicability across diverse material systems. The capability of AFM to generate 3D surface profiles enables precise nanoscale morphological characterization of sample surfaces. However, certain limitations should be acknowledged: AFM typically offers a relatively small imaging area and slower scanning speeds compared to alternative microscopy techniques, making it less suitable for high-throughput analysis of large-area samples. Moreover, the quality and selection of AFM probes play a crucial role in measurement accuracy, necessitating careful probe selection and optimization to ensure reliable and reproducible results.

### 5.6 X-ray Absorption Fine Structure Spectroscopy

XAFS spectroscopy has emerged as a highly sensitive, nondestructive analytical technique capable of probing material structures at the atomic scale. This powerful method, which measures the X-ray absorption coefficient of target elements as a function of incident photon energy, provides detailed information about atomic species, local coordination environments, interatomic distances, and oxidation states.^370,371^ XAFS encompasses two complementary techniques: X-ray absorption near-edge structure (XANES) and extended X-ray absorption fine-structure (EXAFS) spectroscopy. XANES is particularly valuable for determining the oxidation state and local symmetry of absorbing atoms through analysis of electronic transitions, while EXAFS offers quantitative insights into coordination numbers, bond lengths, and the chemical identity of neighboring atoms.^372–374^ Owing to its unique capabilities, including element-specificity and exceptional sensitivity to short-range order, XAFS has become an indispensable tool for investigating the local atomic configurations and electronic structures of layered nanomaterials subjected to various structural engineering strategies, such as heteroatom doping, interlayer engineering, and crystalline-to-amorphous phase engineering.

Jiao et al. adopted Fe K-edge XANES and EXAFS spectra to investigate the detailed coordination environment of Fe atoms in FeSNC (Fe single atoms on S/N codoped porous carbon) and FeNC (Fe single atoms on N-doped porous carbon) SACs.^288^ The Fe K-edge XANES spectra indicated the oxidation states of Fe in above samples between 0 and +3, as the edge positions of FeSNC and FeNC were situated between Fe_2_O_3_ and Fe foil. EXAFS spectra showed that FeSNC and FeNC have major peaks at 1.5 Å assignable to the scattering of Fe–N bond. Notably, a shoulder peak at 1.8 Å attributed to the Fe–S coordination was observed in the FeSNC sample, suggesting the existence of unsymmetrically coordinated Fe–N_3_S_1_ sites. Similarly, Geng et al. characterized the coordination environment of Pd atoms in Pd_SA_/Ti_3*–x*_C_2_T_*y*_ nanosheets prepared by heteroatom doping via XANES and EXAFS spectroscopy.^299^ The Pd K-edge XANES spectra revealed that the edge position of Pd_SA_/Ti_3*–x*_C_2_T_*y*_ was located between Pd foil and PdO, suggesting the oxidation states of Pd between 0 and +2 (Figure 15e). According to its EXAFS spectra, two peaks were found at around 1.9 and 3.0 Å (Figure 15f), corresponding to the first shell of Pd–C coordination and the second shell of Pd–Ti coordination, while no Pd–Pd peak was observed at about 2.5 Å, suggesting that one Pd atom was coordinated by three C atoms at the nearest neighbors and highly atomically dispersed. Zhou et al. characterized the MoO_3_ and H_2_O/Na^+^ cointercalated NH-MoO_3*–x*_ nanobelts by the Mo K-edge EXAFS spectra.^53^ Compared with MoO_3_ nanobelts, NH-MoO_3*–x*_ nanobelts show much lower peak intensity and slight peak position shift, suggesting a significant increase in structural distortion and a slight change in bond length around Mo atoms induced by H_2_O/Na^+^ cointercalation.

Chen et al. synthesized amorphous NiFe-LDHs from crystalline NiFe-LDHs through crystalline-to-amorphous phase engineering via two steps of *n*-BuLi reduction and ethanol oxidization.^338^ For convenience, NiFe-LDHs treated with 2.5 and 0.75 M *n*-BuLi were denoted as NiFe-2.5Li and NiFe-0.75Li, while their intermediate products were denoted as NiFe-2.5mid and NiFe-0.75mid (NiFe-LDHs treated with *n*-BuLi without ethanol), respectively. XANES was employed to analyze the structural transformations during the two processes. The Ni K-edge position of NiFe-0.75mid was between those of Ni foil and NiFe-LDH, while the spectrum of NiFe-2.5mid was similar to that of Ni foil, indicating that the reduction of NiFe-LDH could be controlled by *n*-BuLi concentration, and NiFe-LDH could be reduced to metallic Ni after 2.5 M *n*-BuLi treatment. Following ethanol treatment, the K-edge positions of NiFe-0.75Li and NiFe-2.5Li shifted notably to lower band energy compared with their intermediate products (Figure 15g), indicating a reduction in the Ni valence states during *n*-BuLi treatment and an oxidization during ethanol treatment. The coordination environment of Ni was further investigated by FT-extended EXAFS spectroscopy (Figure 15h). The Ni spectrum of NiFe-LDHs resembled that of Ni­(OH)_2_, exhibiting distinct Ni–O–Ni bond (2.7 Å) and Ni–O bond (1.6 Å) but no Ni–Ni bond (2.1 Å). While the Ni spectrum of NiFe-2.5mid was similar to that of Ni foil showing only Ni–Ni bond, suggesting that Ni was fully reduced to the alloy state after treatment with 2.5 M *n*-BuLi. In contrast, the Ni spectrum of NiFe-0.75mid displayed a broad peak at 1.9 Å associated with the Ni–O and Ni–Ni bonds as well as a peak assigned to the Ni–O–Ni bond, demonstrating partial preservation of the Ni–O structure. After ethanol oxidization, the peaks attributed to the Ni–O–Ni and Ni–O bonds were presented in the spectra of NiFe-2.5Li and NiFe-0.75Li with the disappearance of Ni–Ni bond, demonstrating that the reduced Ni was easily reoxidized. It is worth noting that weaker Ni–O peaks were found in the spectra of NiFe-0.75Li and NiFe-2.5Li compared with that of NiFe-LDH, manifesting a decrease in the coordination number of Ni–O after treatment with *n*-BuLi and ethanol.

As a nondestructive analytical technique, XAFS spectroscopy enables in situ material characterization with atomic-scale resolution while preserving sample integrity. This advanced method provides comprehensive structural information, particularly regarding the local coordination environment surrounding a central atom, including the chemical identity of neighboring atoms, coordination numbers, bond distance distributions, and the degree of structural disorder across multiple coordination shells. However, the implementation of XAFS presents several practical challenges. The technique requires sophisticated and costly synchrotron radiation facilities, which significantly increases operational complexity and experimental costs. Furthermore, the necessity for multiple measurements to achieve statistically reliable data often results in time-consuming experimental procedures and substantial resource investment. Despite these limitations, the unparalleled atomic-scale insights provided by XAFS continue to make it an invaluable tool in materials characterization.

### 5.7 Raman Spectroscopy

Raman spectroscopy has emerged as a powerful analytical technique relying on the inelastic scattering of photons, referred to the Raman scattering effect.^375^ This nondestructive characterization method employs laser-matter interactions to probe the rotational, vibrational, and other low-frequency modes in materials, providing detailed information about chemical composition and structural characteristics.^376^ With its exceptional spectral and spatial resolution, Raman spectroscopy has become an indispensable tool for investigating the structural and electronic properties of layered nanomaterials.^377–379^ The technique has been extensively utilized to characterize critical material parameters, including phase composition, crystallographic orientation, layer thickness, vacancy defects, doping effects, and interlayer vdW interactions in 2D nanomaterials.^66^ These capabilities make Raman spectroscopy particularly valuable for monitoring structural changes induced by various engineering approaches, such as crystal phase engineering, defect engineering, heteroatom doping, and crystalline-to-amorphous phase engineering.

Basu et al. prepared N-doped 1T@2H MoS_2_ through crystal phase engineering by controlling in situ N doping in a sulfur-rich environment.^167^ According to Raman spectroscopy analysis, the 2H bulk MoS_2_ sample exhibits the E_2g_ ^1^ mode at ∼382 cm^–1^ representing in-plane vibration of S and Mo atoms in opposite directions, and the A_1g_ mode at ∼407 cm^–1^ corresponding to out-of-plane vibrations of S atoms relative to Mo atoms. For N-doped 1T@2H MoS_2_ sample, accompanied by the 2H-phase, the emergence of the new E_1g_ (∼273 cm^–1^), J_1_ (∼138 cm^–1^), J_2_ (∼226 cm^–1^), and J_3_ (∼326 cm^–1^) peaks revealing the coexistence of the 1T- and 2H-phase (Figure 15i).

Yang et al. prepared BiO_2–*x*_ nanoparticles by hydrothermal method and further synthesized BiO_2–*x*_ nanosheets through defect engineering (ultrasonic fragmentation treatment).^52^ Raman spectroscopy was utilized to analyze the defect structures of BiO_2–*x*_ nanosheets and BiO_2–*x*_ nanoparticles. Compared with BiO_2–*x*_ nanoparticles, the peak intensities of BiO_2–*x*_ nanosheets at 312, 474, and 612 cm^–1^ decreased significantly (Figure 15j), which could be attributed to an increase in OV content. Joshi et al. prepared a series of rGO films through defect engineering and optimized their microscopic and macroscopic defect densities by varying the energy density and pulse number of excimer laser.^239^ It was found that defect-free graphene exhibited a sharp G peak at ∼1570 cm^–1^. After laser annealing with excimer laser at various energy densities, the obtained defect-rich rGO showed an obvious D peak at ∼1350 cm^–1^, which was caused by the combination of disordered regions and point defects, and could rise with the increase of point defect concentration.

Besides, Raman spectroscopy is also an important means to analyze the surface information and the change process of layered nanomaterials tuned by defect engineering. According to the Raman spectra of Ti_3_C_2_, H_L_–Ti_3_C_2_ (high-temperature treatment for 1 h) and H_H_–Ti_3_C_2_ nanosheets (high-temperature treatment for 2 h), there were three peaks located at 260, 400, and 600 cm^–1^, indicating the presence of a Ti–C bond (Figure 15k).^244^ It is worth noting that the three peak intensities of H_L_–Ti_3_C_2_ and H_H_–Ti_3_C_2_ nanosheets were significantly reduced in comparation with those of Ti_3_C_2_ nanosheets, while a new peak belonging to TiO_*x*_ appeared at 155 cm^–1^ (Figure 15l), demonstrating partial oxidation of Ti_3_C_2_ to TiO_*x*_ after high-temperature treatment. Zhang et al. synthesized tungsten trioxide with coexisting OVs and carbon coating (WO_3*–x*_/C) through defect engineering by using a one-pot carbon-coating method.^349^ The strong characteristic bands at 717 and 808 cm^–1^ attributed to O–W–O stretching vibrations were detected in WO_3_, while low peak intensity was found in WO_3*–x*_/C with peak at 717 cm^–1^ moving to a lower wavenumber at 688 cm^–1^, indicating an altered coordination structure around the W atom. Moreover, the WO_3*–x*_/C sample exhibited two distinct broad peaks at 1592 cm^–1^ (E_2g_ vibration mode of sp^2^-bonded carbon atoms) and 1382 cm^–1^ (A_1g_ vibration mode of carbon atoms), proving that carbon was coated on WO_3_.

Apart from crystal phase engineering and defect engineering, the structural changes caused by heteroatom doping can also be analyzed by Raman spectroscopy. For instance, Yang et al. prepared Fe-doped MoS_2_ through the calcination of MoS_2_ nanosheets and Fe-powders.^271^ In Raman spectra, two peaks at 399.7 and 374.3 cm^–1^ were assigned to the A_1g_ and E_2g_^1^ modes of MoS_2_, which shifted to 401.3 and 374.9 cm^–1^ in Fe-doped MoS_2_ sample due to the lattice distortion caused by Fe doping. Cui et al. successfully prepared O-doped WS_2_ monolayers by CVD, during which O atoms were integrated into SV sites to form W–O bonds.^298^ According to Raman spectra, four Raman modes (the first-order modes of *A*_1_′(Γ), *E′*(*M*), *E*′(Γ), and the second-order mode of 2*LA*(*M*)) were labeled in both O-doped WS_2_ monolayers and undoped WS_2_ monolayers. While the *A*_1_′(Γ) peak of O-doped WS_2_ monolayers shifted to 418.3 cm^–1^ accompanied by intensity increase in comparison with undoped WS_2_ monolayers (416.8 cm^–1^) due to the p-type doping-induced blue shift.

In addition, Kong et al. synthesized 2D ultrathin As/As_*x*_O_*y*_ nanosheets from bulk As powder through crystalline-to-amorphous phase engineering by using ball-grinding and sonication-based liquid exfoliation.^380^ Raman spectra of As/As_*x*_O_*y*_ nanosheets and bulk As showed two peaks at 196 and 258 cm^–1^, which were assigned to the in-plane vibration and out-of-plane vibration modes of arsenic, respectively. Notably, compared with the bulk As, a broadband corresponding to amorphous arsenic with weak peak intensity in the range of 200–260 cm^–1^ was found in As/As_*x*_O_*y*_ nanosheets sample, indicating the amorphization of arsenene following the exfoliation process.

As an analytical technique, Raman spectroscopy offers several distinctive advantages, including exceptional molecular specificity, high sensitivity (with detection limits reaching trace levels), rapid analysis capabilities (typically yielding results within 2 min), and minimal sample requirements (enabling analysis of microscale quantities). This versatile technique is applicable to samples in various states of matter (solid, liquid, and gas) while maintaining sample integrity, and provides unique molecular fingerprints for precise material identification. Furthermore, modern Raman instruments feature user-friendly interfaces and streamlined operation procedures, significantly enhancing experimental efficiency. However, certain technical limitations should be considered: the inherent Raman scattering process generates relatively weak signals due to the small scattering cross-section, which can result in low signal-to-noise ratios. Additionally, fluorescence interference from samples or substrates may obscure Raman features, potentially compromising the accuracy and reliability of spectral interpretation. These limitations can be mitigated through advanced instrumentation and measurement strategies, such as surface-enhanced Raman spectroscopy (SERS) or resonance Raman techniques.^381,382^

### 5.8 Nuclear Magnetic Resonance Spectroscopy and Mass Spectroscopy

NMR spectroscopy is a pivotal technique for elucidating the atomic-scale structure and dynamics of materials, making it essential for the characterization of layered nanomaterials. This method relies on the principle that atomic nuclei (e.g., ^1^H, ^13^C, ^17^O, ^19^F, ^31^P, ^7^Li, etc.) experience energy level splitting in a strong static magnetic field and undergo resonance of nuclear spins when exposed to radiofrequency pulses of specific frequencies.^383,384^ This resonance enables the qualitative analysis of the composition and structure attributes of both organic and inorganic substances. By accurately measuring parameters such as resonance frequency (chemical shift), signal intensity, relaxation times, and spin–spin couplings, NMR provides detailed insights into the chemical environment, bonding configurations, local structural order, and dynamics of molecular motion of the material.^385^ Furthermore, NMR spectroscopy can be synergistically combined with mass spectrometry (MS, which measures the mass-to-charge ratio (*m*/*z*) of ions) to analyze structural information on intercalated bioactive molecules.^386^ These capabilities render NMR spectroscopy exceptionally valuable for tracking structural modifications prompted by various engineering strategies, such as heteroatom doping and interlayer engineering.

For instance, Sideris et al. applied rapid (60 kilohertz) magic angle spinning (MAS) to acquire ^1^H NMR spectra and investigate the distributions of Mg and Al cations in MgAl-LDH with varying Al doping content.^387^ By integrating the ^1^H MAS findings with ^25^ Mg triple-quantum MAS data, they revealed a fully ordered cation arrangement specifically at a Mg:Al ratio of 2:1. At lower Al contents, the cation distribution remained persisted, characterized by an absence of adjacent Al^3+^-Al^3+^ close contacts. Such ordering significantly affects the charge density within the metal hydroxide layers, influencing the bonding characteristics, reactivity, spatial orientation, and mobility of chemical species both within the interlayer region and on the surface. In another study, Zhao et al. employed ^17^O solid-state NMR spectroscopy to characterize the local environments of oxygen atoms in LDHs.^388^ The ^17^O resonances provided superior spectral resolution over ^1^H MAS NMR, due to the distinct oxygen sites within LDHs. Their research identified oxygen atoms in brucite-like Mg­(OH)_2_ domains exhibiting axial symmetry, with Al doping causing a measurable deviation from axial symmetry in the oxygen coordination environment of MgAl-LDHs. These findings underscore the broad applicability and efficacy of rapid MAS NMR techniques in mapping proton distributions across various layered nanomaterials.

Beyond elucidating atomic-scale structure of layered nanomaterials, NMR spectroscopy can also be employed to analyze the structural information on intercalated bioactive molecules, especially when combined with MS. For instance, Hu et al. synthesized organic/inorganic superlattice nanoparticles by intercalating several dye molecules (TPE-I, TPA-I, and PHC-I) into layered MoO_3_ nanobelts.^63^ The structural identities of these dyes were confirmed using ^1^H NMR, ^13^C NMR and high-resolution MS. In another study, Hu et al. explored the structural features of two Ti_3_C_2_T_*x*_ Mxenes, which were produced by etching Ti_3_AlC_2_ with low-concentration (6 mol/L) and high-concentration (15 mol/L) aqueous HF solution.^389^ ^1^H NMR analysis revealed that Ti_3_C_2_T_*x*_-6 M retained a greater number of highly mobile H_2_O molecules within its interlayers compared to Ti_3_C_2_T_*x*_-15M, facilitating enhanced accessibility of hydrogen ions to the active sites of Ti_3_C_2_T_*x*_-6M. Additionally, Piao et al. prepared *n*-alkylamine-intercalated layered titanates and identified two distinct interlayer Na^+^ sites proximate to ammonium head groups/titanate layers and close to the alkyl chains, using ^23^Na MAS NMR spectroscopy.^390^ The intercalation of long-chain molecules increased the population of Na^+^ ions at the latter site, thereby enhancing the electrode activity of the layered titanates.

NMR spectroscopy excels in probing atomic-scale chemical environments, molecular dynamics, and quantitative composition of materials, offering details insights through metrics such as chemical shift, coupling constants, and relaxation times. This technique enables nondestructive, element-specific analysis applicable to both crystalline and amorphous phases. However, its broader application is limited by challenges such as low sensitivity (often requiring isotopic enrichment/large sample volumes), high instrumentation costs, complex interpretation of solid-state data, and paramagnetic interference, which together limit trace-level or in situ applications. Integrating NMR with MS can synergistically enhance capabilities by combining atomic-level structural elucidation (NMR) with high-sensitivity molecular identification (MS). This integration facilitates a comprehensive characterization of complex systems. Despite these advantages, several challenges remain, such as the complexity of real-time integration, high instrumentation costs, potential signal suppression in hyphenated systems (e.g., LC-NMR-MS), and stringent sample preparation requirements for MS-NMR compatibility. To overcome these hurdles, advances in microfluidic-NMR integration, AI-assisted spectral interpretation, and quantum-enhanced sensitivity, are pivotal for enabling resource-efficient, real-time, in situ trace-level analysis of complex systems.

## 6 Biomedical Applications

With the swift advancements in nanotechnology, layered nanomaterials have been recognized as highly promising candidates for biomedical applications, providing unprecedented opportunities in drug delivery, bioimaging, cancer therapy, theranostics, biosensing, antibacteria, etc. In this section, all biomedical applications of layered nanomaterials fine-tuned through structural engineering will be introduced with a particular emphasis on those pioneering works and those demonstrating exceptional performance. Additionally, the underlying mechanisms by which structural engineering strategies optimize the performance of layered nanomaterials for diverse biomedical applications will be thoroughly discussed.

### 6.1 Drug Delivery

The primary objectives of drug delivery via layered nanomaterials are to achieve controlled release kinetics, prolong plasma circulation time, enhance drug bioavailability, promote targeted accumulation at pathological sites, minimize systemic toxicity, and shield therapeutic agents from enzymatic degradation.^391–397^ Among diverse structural engineering strategies, interlayer engineering has emerged as the predominant strategy for designing advanced drug delivery systems based on layered nanomaterials, especially LDHs, owing to its precise spatial control over molecular encapsulation and stimuli-responsive release capabilities. For example, a drug delivery system proposed by Bao et al. for bladder cancer was prepared by intercalating ethylene diamine tetraacetic acid (EDTA) into ZnAl-LDH interlayer via ion exchange for sustained drug release and enhanced anticancer activity.^398^ In a pH 6.5 acidic environment, the release of EDTA from ZnAl-LDH occurred rapidly during the initial hour and persisted over time, reaching a release amount of 25.8 wt % within 16 h. Since EDTA can sequester Ca^2+^ from intercellular connexin through EDTA-Ca^2+^ chelation to disrupt tumor integrity and promote disaggregation, this sustained release of EDTA ensured biosafety and provided an adequate supply of EDTA for Ca^2+^ capture, eventually leading to efficient ablation of bladder tumors.

Recently, Xing et al. constructed an arsenic-intercalated NiTi-LDHs film via ion exchange for gallbladder cancer treatment (Figure 16a).^313^ Since Ni^2+^ in NiTi-LDHs easily lost electrons to form highly oxidizing Ni^3+^ that can react with H_2_O to generate ·OH, NiTi-LDHs could act as a “processor” to catalyze the oxidation of intercalated highly toxic As­(III) ions to lowly toxic As­(V) ions by virtue of ·OH. NiTi-LDHs also functioned as a “quality-inspector” to confine As­(III) in the interlayer and only release As­(V) to the outside, thereby minimizing toxicity upon contact with normal tissue. More importantly, the overexpressed GSH and acidic microenvironment not only accelerated the release of arsenic from LDHs interlayer, but also promote the reduction of low-toxic As­(V) to high-toxic As­(III), thus exerting a strong TEM-responsive antineoplastic effect. In vitro and in vivo assays fully proved that arsenic-intercalated NiTi-LDHs film efficiently prevented tumor overgrowth, metastasis, and postoperative bacterial infection for gallbladder cancer (Figure 16b–d).

**Figure 16:**
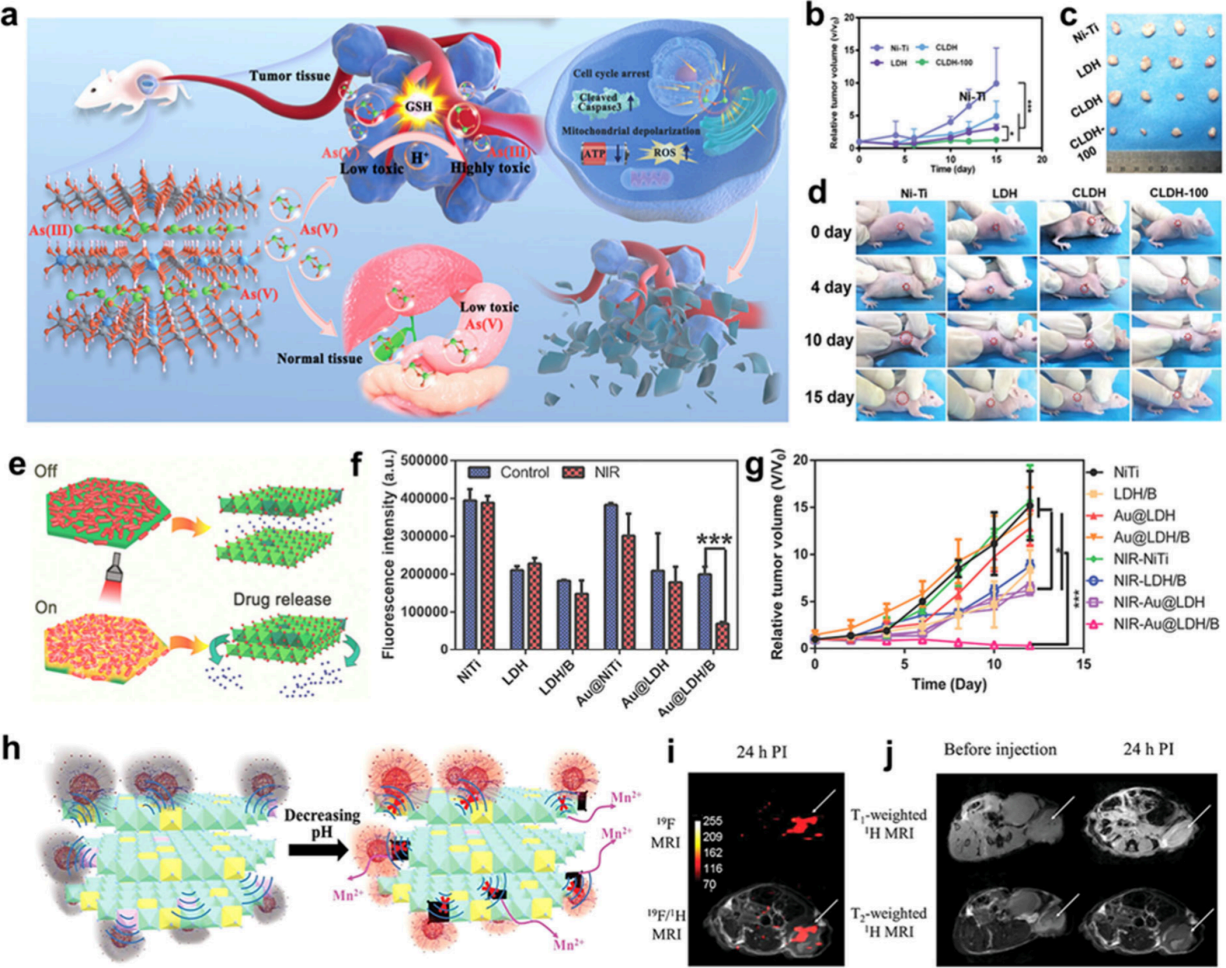
(a) Schematic diagram of therapeutic mechanism of arsenic-intercalated NiTi-LDHs. (b) Relative volume growth curves of mice after different treatments. (c) Representative photographs of tumor-bearing mice after different treatments. (d) Photograph of tumors taken on the 15th day. Reproduced with permission from ref 313. Copyright 2022 John Wiley and Sons. (e) Illustration of the mechanism of the interlayer ions release from Au@LDH/B sample. (f) Viability of cancer cells treated with different samples after NIR irradiation. (g) Relative tumor volume of mice after different treatments. Reproduced with permission from ref 348. Copyright 2018 John Wiley and Sons. (h) The mechanism of Mn-LDH@PFPE for pH-activated ^19^F MRI. (i) ^19^F MRI images of mice after intravenous injection of Mn-LDH@PFPE nanoparticles. (j) T_1_ and T_2_-weighted ^1^H MRI images of Mn-LDH@PFPE nanoparticles. Reproduced with permission from ref 256. Copyright 2019 John Wiley and Sons.

Apart from pH- or TEM-responsive drug release, exogenous stimuli such as heat or light can also mediate the controlled release of drugs. Wang et al. constructed an intelligent LDH-based drug delivery system (Au@LDH/B) by intercalating butyrate (an anticancer agent) into NiTi-LDHs film and loading GNR for localized chemothermal tumor therapy.^348^ Owing to the photothermal performance of GNRs, NIR could trigger the temperature increase of Au@LDH/B. Under 808 nm laser irradiation, the contact area between GNRs and LDH/B film experienced a significant temperature increase, reaching up to 271 °C, thereby leading to the crystal phase transformation of NiTi-LDHs toward LDOs and the release of butyrate (Figure 16e). Interestingly, when NIR irradiation was halted, the crystal phase of LDOs reverted to LDHs in water by reabsorbing excessive drugs from the surrounding environment. This intelligent drug-loading film exhibited controlled drug release behavior and synergistic chemothermal therapy against tumor cells (Figure 16f,g), providing a new paradigm for the potential application of localized drug-eluting systems.

In addition to delivering anticancer drugs, layered nanomaterials can also be employed to deliver anti-inflammatory and functional drugs, such as ibuprofen, diclofenac, mefenamic acid, para-aminosalicylic acid (PAS), thymoquinone, low molecular weight heparin (LMWH), vitamin C, etc. Yousef et al. reported the successful intercalation of ibuprofen or diclofenac into the interlay space of Fe_3_O_4_@LDH preprepared by coprecipitation via ion exchange method.^399^ Thanks to the strong bridging bidentate interaction between the carboxyl groups with trivalent cations, Fe_3_O_4_@LDH-ibuprofen and Fe_3_O_4_@LDH-diclofenac exhibited high thermal stability. In the physiological solution environment (pH 7.4), Fe_3_O_4_@LDH-ibuprofen and Fe_3_O_4_@LDH-diclofenac showed constant release behavior, avoiding the serious side effects of high plasma levels after direct administration of ibuprofen and diclofenac. The release rates of ibuprofen and diclofenac were 96% and 82% within 72 h. The less diclofenac release could be attributed to its lower solubility in water and more sterile effect. Similarly, Abniki et al. prepared mefenamic acid-intercalated CaAl-LDH (ME/LDH) via ion exchange route for controlled drug delivery.^400^ It was found that the release percentage of ME from ME/LDH within 900 min was 58% and 75% in acidic and phosphate-buffered environments, respectively. Such a pH-responsive release behavior was primarily due to the dissolution of hydroxide layers of LDH in an acidic media, and the release of ME occurred through the degradation of LDH layers and ion exchange process.

Recently, Saifullah et al. designed an antituberculosis nanodelivery system (MgLH-PAS) by intercalating PAS into MgLH for the treatment of tuberculosis.^153^ It was found that the release behavior of PAS in MgLH-PAS was sustained, avoiding the side effects such as anorexia, nausea, vomiting, or diarrhea caused by burst release. The release duration was close to 24 h, indicating that MgLH-PAS could maintain the concentration of PAS in the human body for 24 h, significantly improving the bioavailability of PAS and thus enhancing the therapeutic effect. Jeyakumar et al. reported the synthesis of thymoquinone-intercalated ZnFe-LDH [TQ-ZnFe-LDH] for accelerated bone mineralization by promoting osteoblastic differentiation and proliferation.^306^ Owing to the synergistic effect of thymoquinone, Zn and Fe ions that can upregulate the bone morphogenetic protein and augment osteoblast differentiation, TQ-ZnFe-LDH exhibited enhanced osteoconductivity and osteoinductivity by accelerating calcium deposition and subsequent mineralization. Gu et al. engineered a system by intercalating LMWH (an anticoagulant) into MgAl-LDHs using a coprecipitation method.^401^ This innovative design enabled the sustained release of LMWH from LDHs, effectively addressing its inherent pharmaceutical limitation of short half-life. By gradually releasing LMWH over time, the system maintained therapeutic drug levels in the bloodstream, prolonging its anticoagulant activity and enhancing its clinical efficacy in anticoagulation therapy. In another study, Gao et al. prepared vitamin C-intercalated CaAl-LDHs using the same method and demonstrated that the release time of vitamin C in phosphate buffer was significantly extended, achieving sustained release of vitamin C.^402^

In addition, Lee et al. engineered a bioactive 2*D*/3D nanocomposite system by integrating arginylglycylaspartic acid-functionalized LDHs, tonsil-derived mesenchymal stem cells (TMSCs) and kartogenin (KGN) into a thermoresponsive gel matrix through the thermal-driven gelation.^403^ The incorporation of inorganic LDHs enhanced the rigidity of the hydrogel matrix, provided sustained release of a soluble factor (KGN) and promoted favorable cell-material interactions. Crucially, results revealed significantly elevated expression levels of key chondrogenic markers (type II collagen and transcription factor SOX9) in this nanocomposite compared to pure thermogels. Consequently, the current LDH/thermogel 2*D*/3D nanocomposite system represents a promising injectable platform for stem cell-based tissue engineering and therapeutic applications. Similarly, the same group also proposed G/PEG–PA, GO/PEG–PA, GO/PEG-L-PA, and rGO/PEG-L-PA 2*D*/3D hybrid system for drug delivery and stem cell differentiation control.^404,405^ Notably, there are still relatively few reports on the use of structural engineering strategies to tailor the properties of 2*D*/3D composite materials for biomedical applications. Consequently, future research focusing on this area is anticipated to broaden the scope of 2*D*/3D composites in biomedical fields.

A critical consideration for layered nanomaterial drug carriers involves their solute–solvent interactions and potential reactions within biological fluids. Vasti et al. examined these dynamics using positively charged MgAl-LDH nanoparticles (LDH-NPs) and negatively charged giant unilamellar vesicles (GUVs).^406^ They observed that electrostatic attractions led to the accumulation of LDH-NP at the GUV membrane, which altered the phospholipid distribution and increased both membrane stiffness and permeability. Importantly, this interaction was mitigated by passivating the LDH-NP surface with either albumin (LDH@ALB) or poly­(acrylic acid) (LDH@PA), effectively eliminating the long-range electrostatic attraction and preventing membrane-mediated internalization. This modification presents a potential advantage for the use of LDH-NPs in drug or nucleic acid delivery, as target delivery can be enhanced by preventing nonspecific uptake through appropriate surface functionalization. The authors also highlighted that upon contact with biological fluids, LDH nanocarriers experience complex and dynamic changes on their surfaces, including protein corona formation and aggregation/stability shifts.^407^ Crucially, the interaction between LDH nanocarriers and cells is governed by their physicochemical properties, with size, morphology, and interfacial characteristics (charge, potential, hydrophobicity, steric hindrance) being key determinants of cellular internalization.

In conclusion, interlayer-engineered layered nanomaterials hold significant potential as platforms for controlled and sustained drug delivery. However, current limitations hinder their practical application as ideal drug carriers. While existing systems often utilize these nanomaterials merely as passive vehicles to enhance drug stability and bioavailability, they frequently lack intrinsic functional properties capable of synergizing with therapeutic agents. To address this, future research should prioritize the design of multifunctional layered nanomaterials that actively participate in therapeutic mechanisms, for example, by integrating stimuli-responsive, catalytic, or targeting functionalities. Furthermore, expanding the repertoire of stimuli-responsive release triggers beyond conventional pH, light, and thermal activation is critical. Novel exogenous/endogenous stimuli (e.g., enzymatic activity, redox gradients, or US) could enable spatiotemporally precise drug release, improving therapeutic specificity. Additionally, systematic investigations are needed to evaluate how complex physiological environments, particularly competitive ion exchange processes involving coexisting ions, impact drug release kinetics and efficiency. Finally, challenges such as scalability, reproducibility, and long-term biocompatibility must be rigorously addressed to advance interlayer-engineered nanocarriers toward clinical translation. Bridging these gaps will require interdisciplinary collaboration to unlock the full potential of layered nanomaterials in precision medicine and next-generation drug delivery systems.

### 6.2 Bioimaging

Imaging diagnosis represents another prominent field in biomedical applications, playing a pivotal role across various stages, including disease screening, treatment efficacy monitoring, and disease recurrence detection.^408–410^ Over the years, remarkable advancements have been achieved in a variety of cancer imaging techniques, including but not limited to MRI, fluorescence imaging, computed tomography (CT) imaging, US imaging, and photoacoustic imaging (PAI)), which provide detailed structural information about target tissues, enhancing diagnostic accuracy and therapeutic guidance.^411–413^ To date, a series of imaging contrast agents have been proposed by functionalizing layered nanomaterials through structural engineering strategies, especially heteroatom doping and interlayer engineering, for MRI, fluorescence imaging, US imaging, and PAI, demonstrating their versatility and potential to improve imaging performance.

For instance, Peng et al. fabricated Gd^3+^-doped MgAl-LDH nanosheets using facile bottom-up approach, where Gd^3+^ ions were located in the LDH host matrix.^160^ Gd^3+^-doped MgAl-LDH nanosheets exhibited excellent contrast agent capability on T_1_-weighted MRI, of which T_1_-weighted relaxivity (r_1_, 7.93 × 10^–3^ m^–1^ s^–1^) is ∼2.27 times that of the commercial contrast agent Gd-diethylenetriaminepentaacetic acid with a known relaxivity of approximately 3.5 × 10^–3^ m^–1^ s^–1^. The same group further prepared Gd^3+^/Yb^3+^ codoped MgAl-LDH nanosheets for MRI/CT dual-mode imaging.^161^ Li et al. prepared Mn-doped MgAl-LDH (Mn-LDH) as a pH-responsive T_1_-weighted MRI contrast agent by ion exchange method.^257^ Mn-LDH was found to have exceptional longitudinal relaxivity with values of 1.16 mM^–1^ s^–1^ at pH 7.4, 6.82 mM^–1^ s^–1^ at pH 7.0 and 9.48 mM^–1^ s^–1^ at pH 5.0, superior to that of free Mn^2+^ ions, which could be attributed to the unique microstructural coordination environment surrounding Mn atoms in Mn-LDH that favored the T_1_ relaxation of surrounding protons. Notably, compared with pH 7.4, the shrinking of Mn···OH_2_ bond at pH 5.0 led to a decrease in the longitudinal relaxation time of surrounding protons, thereby enhancing T_1_-weighted MRI contrast agent capability.

Similarly, Zhang et al. designed Mn-doped MgAl-LDH nanoparticles conjugated with perfluoropolyether (Mn-LDH@PFPE) via ion exchange strategy as a contrast agent for pH-activated ^19^F MRI (Figure 16h).^256^ The results showed that the ^19^F NMR/MRI signal from Mn-LDH@PFPE nanoparticles was effectively quenched and became undetectable at pH 7.4, but “turned on” and became detectable at pH 6.5. This phenomenon could be explained as follows: the ^19^F nuclei was tightly associated with the surface of LDHs and positioned close to Mn^2+^ within the host matrix when pH = 7.4, and thus undergone intense paramagnetic relaxation resulting from Mn^2+^ ions to shorten the T_2_ relaxation time with undetectable ^19^F MRI signal; while the close proximity between the Mn^2+^ ions and ^19^F nuclei was broken stepwise due to the etching away of Mn^2+^ ions from Mn-LDH when pH = 6.5, and the ^19^F T_2_ was restored to its intrinsic value with strong ^19^F MRI signal. As expected, an intense ^19^F MRI signal was specifically detected at the tumor site following intravenous administration of Mn-LDH@PFPE nanoparticles (Figure 16i,j).

Apart from MRI, layered nanomaterials can also be tuned by heteroatom doping strategies to possess fluorescence properties. Thomas et al. reported a highly fluorescent heteroatom-doped graphene material (N-GO) for cellular bioimaging.^252^ It was found that the N doping had a great effect on the photophysical properties of N-GO, giving rise to prolonged fluorescence lifetime (8.51 ns), increased quantum yield (16%), and improved photostability (92%). Encouraged by its excellent fluorescence emission properties, N-GO has demonstrated significant potential as a biomarker for in vitro imaging. Its strong and stable fluorescence enabled easy and effective labeling of human skin cancer cell lines and human macrophage cell lines. Similarly, Tewari et al. prepared K-doped GO by a one step hydrothermal route, which demonstrated bright blue photoluminescence under UV light irradiation and could serve as an excellent bioimaging agent.^276^

Cui et al. successfully prepared O-doped WS_2_ monolayers by CVD, during which O atoms were integrated into SV sites to form W–O bonds.^298^ The photoluminescence quantum yield of O-doped WS_2_ monolayers (9.3%) was significantly enhanced, showing an improvement of nearly 2 orders of magnitude compared with pristine WS_2_. Moreover, W–O bonds enabled robust and enduring photoluminescence of O-doped WS_2_ monolayers for up to 3 months under ambient conditions without requiring any protective measures. This superior environment stability stemmed from the repair of SV sites through the formation of saturated coordination bonds. Similarly, Hai et al. reported that B-GQDs prepared by one-pot acid-free microwave approach exhibited favorable photoluminescence behavior due to the doping of boron atom and the repair of defects in the graphene structure, with a fluorescence quantum yield of 21.1%.^301^ In vitro assays demonstrated that B-GQDs with good biocompatibility could be used for bioimaging of HeLa cells.

In addition to heteroatom doping, interlayer engineering has also been proposed to endow layered nanomaterials with photoluminescence properties for bioimaging. Liu et al. fabricated fluorescent CN/LDH through interlayer engineering strategy (coprecipitation followed by microwave synthesis) for fluorescence imaging.^149^ The CN/LDH had an absolute solid-state quantum yield of 95.9 ± 2.2%, which was the highest reported for carbon-based fluorescent materials at that time. The extremely high solid-state quantum yield of CN/LDH could be attributed to the confinement effect exerted by the host–guest interaction, which significantly inhibited electron transfer and promoted e^–^–h^+^ recombination. In addition, the LDHs host matrix could act as a spacer to isolate ultrathin CN and avoid aggregation-induced fluorescence quenching. Moreover, a covalent bonding might occur between LDHs matrix and CN, thus forming highly emissive species. Therefore, the proposed CN/LDH could be used as a two-photon bioimaging agent with satisfactory biocompatibility and nontoxicity for cell imaging. Xue et al. reported the preparation of a series of monolayer Ti_3_C_2_ MXene QDs by a hydrothermal-mediated interlayer engineering strategy.^337^ It was found that Ti_3_C_2_ QDs exhibited excitation-dependent photoluminescence spectra with quantum yields of approximately 10% owing to its strong quantum confinement effect. Moreover, the potential of Ti_3_C_2_ QDs as biocompatible cellular imaging probes was demonstrated through their successful application in labeling RAW264.7 cells, underscoring their enormous prospect for use in biomedical applications, such as cellular imaging, diagnostics, and targeted drug delivery.

In conclusion, layered nanomaterials tuned by structural engineering strategies have garnered significant attention as promising contrast agents for various imaging techniques, owing to their unique structural and functional properties. However, their current application is predominantly limited to single-modal imaging, which provides restricted biological information. Given the potential to integrate multiple imaging modalities into a single nanoplatform, layered nanomaterials can be strategically engineered to exhibit combined functionalities, enabling the acquisition of more comprehensive structural information about target tissues compared to single-modal imaging contrast agents. Furthermore, exploring additional structural engineering strategies is essential to optimize the structural and functional properties of layered nanomaterials, thereby expanding their applicability to a broader range of imaging techniques. Overall, the design of layered nanomaterials as in vivo imaging contrast agents is still in its early stages. Significant opportunities remain for the discovery and advancement of novel, high-performance layered nanomaterial-based imaging contrast agents through innovative structural engineering approaches.

### 6.3 Cancer Therapy

Cancer remains one of the most significant global health challenges, ranking among the leading causes of death worldwide, with both morbidity and mortality rates continuing to rise annually.^414–417^ To enhance diagnostic accuracy and therapeutic outcomes, numerous layered nanomaterials have been engineered and fine-tuned using various structural engineering strategies, and have been applied across a broad spectrum of cancer treatments, including PTT, PDT, SDT, catalytic therapy, CDT, immunotherapy, etc. In this section, we will highlight the latest progress in the development of layered nanomaterial-based platforms for cancer treatment, showcasing their potential to revolutionize the field.

#### 6.3.1 Photothermal Therapy

As a noninvasive therapeutic approach, PTT relies on photothermal agents to absorb NIR light and convert it into localized hyperthermia, inducing thermally driven cell killing while minimizing damage to surrounding healthy tissues.^418–420^ Nervertheless, the PCE of most reported photothermal agents remains suboptimal, leaving significant room for improvement.^421,422^ Fortunately, structural engineering strategies such as crystal phase engineering, defect engineering, heteroatom doping, and interlayer engineering, offer a promising avenue to address these limitations, enabling the optimization of the photothermal performance of layered nanomaterials by precisely tuning their crystal phase, electronic structure, and defect configurations, thereby enhancing their therapeutic efficacy.

In 2013, Chou et al. first employed Li intercalation-mediated crystal phase engineering strategy to prepare chemically exfoliated MoS_2_ with a mixed 1T/1T′-phase as a photothermal agent.^154^ During the intercalation process, the electron transfer from *n*-BuLi to MoS_2_ induced the phase transformation from 2H-phase to metallic 1T/1T′-phase, thus endowing the MoS_2_ nanosheets with excellent photothermal performance. This is the first case that MoS_2_ was designed as a photothermal agent through phase engineering strategy. However, in this study, the crucial role of the metallic phase in enhancing its photothermal performance was not yet revealed. Until 2020, Zhou et al. prepared 1T-phase MoS_2_ nanodots from the microsized 2H-phase MoS_2_ crystals by ball milling and Li intercalation-induced exfoliation method, and revealed that the photothermal performance of 1T-MoS_2_ outperformed that of 2H-MoS_2_ owing to the metallic phase-enabled strong absorption under laser irradiation.^59^ Since then, a series of metallic 1T/1T′-phase MoS_2_ nanodots/nanosheets and 1T-phase MoSe_2_ nanosheets proposed by Chen et al. and Huang et al. have also been demonstrated to possess excellent photothermal performance.^172,173^

Recently, Rusciano et al. prepared MoS_2_ nanosheets via sonication-assisted liquid-phase exfoliation and investigated their photothermal effect at the single-cell level via confocal micro-Raman spectroscopy.^423^ The results revealed that localized temperature increase occurred around MoS_2_ aggregates in targeted MCF7 cells upon laser irradiation. Raman mapping further confirmed that these aggregates were confined to regions near the cell membrane, indicating their inability to penetrate the cells. The observed preferential localization of MoS_2_ aggregates on the outer membrane surface suggests that the induced thermal effects could directly impact the membrane’s complex architecture, including its phospholipid bilayer and the regulatory proteins that manage intracellular/extracellular exchanges. These findings lay the foundation for further exploration of MoS_2_’s cellular interactions and offering potential to probe structural evolution of specific biomacromolecules (e.g., lipids, proteins) under thermal stress conditions.

In addition, the influence of layer number of MoS_2_ nanosheets on antitumor performance was also investigated. Kaur et al. prepared few-layered MoS_2_ nanosheets via direct exfoliation in pure water and evaluated their cytotoxic effects against three cell lines (MCF7 (breast cancer), U937 (leukemia), and HaCaT (epithelium)).^163^ The results revealed that MoS_2_ nanosheets with 2–8 layers induced significant cell death in both cancer cell lines (MCF7 and U937), while exhibiting minimal cytotoxic effects in the normal cell line (HaCaT). This selective cytotoxicity suggested their potential as a targeted anticancer system.

Apart from crystal phase engineering, Zhu et al. developed a new photothermal nanoagents RuS_*x*_ nanoclusters via defect engineering for the optimized PTT.^236^ Specifically, RuS_*x*_ nanoclusters containing different oxygen contents and sulfur defects could be obtained by adjusting the initial molar ratios of Ru to S. As the Ru-to-S molar ratio increased, RuS_*x*_ nanoclusters were endowed with increased oxygen contents and sulfur defects, leading to a significant enhancement of PCE from 32.8 to 41.9%, surpassing that of other inorganic photothermal nanoagents. The optimized RuS_*x*_ nanoclusters demonstrated an exceptional photothermal effect, enabling efficient and complete ablation of cancer cells under 808 nm laser irradiation. Miao et al. developed ultrathin NbSe_2_ nanosheets with abundant atomic defects via DNA-assisted exfoliation strategy for anti-inflammation and antitumor theranostics.^197^ Owing to its high RONS scavenging efficiency based on hydrogen atom transfer and redox reaction, NbSe_2_ nanosheets efficiently inhibited lipopolysaccharide-induced rear thigh inflammation. In addition, NbSe_2_ nanosheets showed strong NIR absorbance, enabling rapid PAI-guided tumor ablation under 808 nm laser irradiation. Moreover, the PTT-mediated inflammation was also inhibited after treatment with NbSe_2_ nanosheets.

In addition to the above crystal phase engineering and defect engineering, Deng et al. employed a heteroatom doping strategy (thermal sintering) to design and construct a gallium-doped V_2_C MXene nanoenzyme with an amino-functionalized surface (Ga/V_2_C-NH_2_) for anti-inflammation and photoenhanced antitumor therapy of colon diseases.^270^ Benefiting from multiple enzyme-mimicking activities like SOD, CAT, and POD, Ga/V_2_C-NH_2_ effectively scavenged ROS, reduced proinflammatory cytokines, and inhibited ROS-mediated inflammatory reactions, thereby safeguarding cells and organs from potential harm. In addition, the doping of Ga^3+^ not only disturbed Fe^3+^ uptake, iron metabolism and DNA replication in cancer cells, but also improved the NIR laser absorption ability of V_2_C MXene to enhance the PCE, thus exerting a more effective PTT effect on cancer cells. Therefore, Ga/V_2_C-NH_2_ achieved efficient chemo-photothermal-mediated tumor ablation under 808 nm laser irradiation. Similarly, Huang et al. developed Er-doped WSe_2_ nanosheets as a photothermal agent with a PCE of 35.2%, which was higher than pristine WSe_2_ nanosheets due to the doping of Er ion enhancing the absorption under 808 nm laser irradiation.^272^ Based on the same principle, Wang et al. also proposed N- and B-codoped graphene quantum dots (N-B-GQDs) for enhanced PTT.^424^

Since the NIR-II light (1000–1350 nm) has longer tissue penetration depth than the NIR-I light (750–1000 nm),^425^ various PTT strategies on the basis of NIR-II absorption have also been successively developed. Guo et al. designed carbon defect-enriched B_4_C@C nanosheets through defect engineering by hydrothermal calcination of B_4_C and glucose for NIR-II PTT.^227^ The presence of carbon defects could capture photogenerated electrons and enhance the light absorption performance of B_4_C@C nanosheets in the NIR-II region, which is conductive to translating light energy into heat energy through nonradiative transition, resulting in higher PCE compared with B_4_C (45.4% vs 17.7%) under 1064 nm laser irradiation. In vitro and in vivo results demonstrated that B_4_C@C nanosheets effectively killed cancer HeLa cells and completely eliminated tumors under 1064 nm laser irradiation. Zhou et al. prepared defective MoO_3*–x*_ nanobelts with strong NIR-II absorption through lithium treatment.^60^ Specifically, lithium treatment induced a structural transformation from micrometre-long MoO_3_ nanobelts (white color) to interlayer-expanded MoO_3*–x*_ nanobelts (blue color). Compared with crystalline MoO_3_ nanobelts, the defective MoO_3*–x*_ nanobelts exhibited a high PCE of 46.9% and a large extinction coefficient of 18.2 L g^–1^ cm^–1^ at 1064 nm, attributing to the activation of NIR-II absorption induced by intercalation engineering. After modification with PVP, the PVP-MoO_3*–x*_ nanobelts served as a photothermal reagent for high-efficiency PTT, achieving efficient cancer cell apoptosis and tumor eradication under 1064 nm laser irradiation.

#### 6.3.2 Photodynamic Therapy

PDT is a clinically established treatment method characterized by its reduced side effects, high selectivity and minimal invasiveness, which involves the activation of PSs by visible or NIR light, leading to the generation of ROS (e.g., ^1^O_2_, ·O_2_^–^ and ·OH) to induce cancer cell death.^426–428^ Traditional PSs, however, were primarily activated by visible light, which suffers from limited tissue penetration depth, thereby restricting their efficacy in PDT applications.^429,430^ In recent years, advancements in structural engineering strategies, including defect engineering, heteroatom doping, interlayer engineering, and crystalline-to-amorphous phase engineering, have led to the development of various PSs capable of being excited by NIR light (“optical window” of biological tissue with minimum tissue scattering). These innovations have enabled the emergence of PSs suitable for both NIR-I and NIR-II PDT, significantly expanding the therapeutic potential of PDT by improving tissue penetration and treatment efficacy.

##### 6.3.2.1 NIR-I PDT

As a typical example, Gao et al. proposed a NIR-I activated supramolecular PS (IPA/LDH) by coprecipitation-mediated interlayer engineering for efficient two-photon PDT.^148^ The intercalated IPA molecules were arranged in an orderly manner in the interlayer space of LDHs due to the surface- and space-confinement effects mediated by LDHs, facilitating the generation of long-lived triplet excitons with second-scale lifetimes induced by two-photons, thereby boosting the ^1^O_2_ generation under 808 nm laser irradiation with a ^1^O_2_ quantum yield of 0.74. In vitro and in vivo assays demonstrated that IPA/LDH exhibited strong ability in killing cancer cells and ablating tumors under 808 nm laser irradiation with extremely low biological toxicity. This work may be the first case of achieving two-photon PDT with a single material. After that, Wu et al. synthesized three engineered g-C_3_N_4_ QDs (CN, CN-DPT, and CN-THDT) through defect engineering (calcination and subsequent simple quenching process) for two-photon imaging (TPI) and PDT.^242^ The ability of three g-C_3_N_4_ QDs for TPI and PDT could be modulated under 800 nm light irradiation. Interestingly, the integration of DPT or THDT introduced additional lattice defects and increased the disordered structure within the g-C_3_N_4_ framework, resulting in defect energy level between valence band and conduction band, which could suppress photogenerated carrier recombination and promote ROS production. As expected, CN-DPT and CN-THDT QDs exerted superior TPI abilities and generated more ROS than CN QDs. In comparison, CN-DPT that presented balanced abilities for TPI and TPE-PDT demonstrated highly efficient tumor cell killing effect under 800 nm light irradiation by interfering with mitochondrial function.

##### 6.3.2.2 NIR-II PDT

Shen et al. engineered defect-rich CoMo-LDH nanosheets through acid etching-mediated defect engineering as high-efficiency inorganic PSs for NIR-II PDT.^56^ Thanks to the defect engineering-induced electronic structure changes, e.g., narrow bandgap, more negative conduction band position, suppressed e^–^–h^+^ recombination, defect-rich CoMo-LDH nanosheets exhibited enhanced ROS generation performance when exposed to 1567 nm laser, whose activity was ∼97 times that of pristine CoMo-LDH with a relative ^1^O_2_ quantum yield of 0.87. After PEGylation, the defect-rich CoMo-LDH nanosheets efficiently induced cancer cells apoptosis and eradicated tumors upon 1567 nm laser irradiation. To further improve the targeting ability of LDH-based PSs, Yang et al. also reported a TME-responsive PS (*LA*&LDH) through the coupling of *LA* with CoCuMo-LDH nanosheets.^58^ CoCuMo-LDH nanosheets underwent a transformation from a crystalline to an amorphous structure through in situ etching, driven by the low pH induced by *LA* metabolites and the overexpressed GSH in the TME. This structural change significantly boosted the nanosheets’ ROS generation capability under 1270 nm laser irradiation, achieving a relative ^1^O_2_ quantum yield of 1.06. As expected, the TME-responsive *LA*&LDH effectively induced complete cell apoptosis and tumor eradication, demonstrating its ability to achieve precise and targeted NIR-II PDT.

Most recently, the same group further proposed a series of metal-doped amorphous CoMo-LDH nanosheets (a-M-CoMo-LDH, M = Zn, Mg, Ni, Al, Cu, Mn) through a combination of one-step hydrothermal and acid etching methods for NIR-II PDT.^327^ The generation of a large number of defects and the reduction of bandgap caused by metal doping and acid etching synergistically strengthened the ROS generation performance of LDH by elevating the separation efficiency of e^–^–h^+^ pairs. Especially, a-Zn-CoMo-LDH nanosheets exhibited the highest activity toward ROS generation compared with other a-M-CoMo-LDH nanosheets under 1270 nm laser irradiation. Meanwhile, the ROS generation activity of a-Zn-CoMo-LDH nanosheets was 3.9 times that of a-CoMo-LDH nanosheets without Zn doping, demonstrating the advantage of metal doping in improving ROS generation performance. The ^1^O_2_ quantum yield of a-Zn-CoMo-LDH nanosheets up to 1.86 is the highest among all the reported PSs. As a result, PEG-modified a-Zn-CoMo-LDH nanosheets exerted potent NIR-II PDT performance in killing cancer cells and eliminating tumors under 1270 nm laser irradiation.

In addition, Singh et al. prepared 2D-MoS_2_ nanoflakes using a modified liquid-phase exfoliation technique and for the first time investigated the effects of administering small doses of 511 keV photons to these nanoflakes in water solution.^431^ It was found that the high-energy photons were capable of generating various ROS in water solution through water radiolysis. This discovery opens up a promising new field for investigating the impact of high-energy irradiation on 2D nanomaterials, with potential applications in radiotherapy, oncology, and aerospace biomedical fields.

#### 6.3.3 Sonodynamic Therapy

SDT is an emerging medical approach that combines US with sonosensitizers to generate ROS for the treatment of malignant tumors, offering significant advantages, including minimal side effects, exceptional tissue penetration depth, and high therapeutic precision.^432–434^ Unfortunately, the clinical translation of SDT is significantly hampered by the limited ROS generation efficiency of most conventional sonosensitizers.^435,436^ Recent advancements in structural engineering strategies, such as defect engineering, heteroatom doping, crystalline-to-amorphous phase engineering, etc., have demonstrated remarkable efficacy in elevating the ROS yield of layered nanomaterial-based sonosensitizers, thereby opening new avenues for the development of SDT.

Defect engineering is the most dominant and widespread strategy for constructing layered nanomaterial-based sonosensitizers. For example, Li et al. successfully fabricated H–Ti_3_C_2_ nanosheets with abundant OVs by a two-step process of chemical exfoliation and high-temperature treatment as a sonosensitizer for photothermal-enhanced SDT.^244^ Owing to the presence of OVs promoting e^–^–h^+^ separation and preventing their recombination, H–Ti_3_C_2_ nanosheets exhibited excellent ROS generation performance under US irradiation, which was 3.7 times that of Ti_3_C_2_ nanosheets without high-temperature treatment. Moreover, the H–Ti_3_C_2_ nanosheets had obvious NIR-II absorbance and could generate mild photothermal effect to alleviate the hypoxic microenvironment under 1064 nm laser irradiation. In vitro and in vivo assays demonstrated the synergistic therapeutic effects of H–Ti_3_C_2_ nanosheets on killing cancer cells and suppressing tumor growth. Based on the role of OVs in promoting ROS production by narrowing bandgap, facilitating e^–^–h^+^ separation and inhibiting their recombination, a series of inorganic sonosensitizers have been developed for SDT, such as oxygen-deficient Bi-HJ,^217^ oxygen-deficient MnWO_*x*_ nanoparticles,^228^ OV-rich WO_3*–x*_ nanosheets,^64^ and WO_*x*_ nanobelts.^232^

Besides, Yang et al. successfully synthesized ultrathin BiO_2–*x*_ nanosheets containing rich vacancies with excellent piezoelectric properties via sonication-assisted liquid-phase exfoliation.^52^ Under US irradiation, the defect-rich BiO_2*–x*_ initiated a cascade reaction of ROS generation, as its piezo-potential of 0.25 V could shift the conduction band to a more negative position relative to the redox potentials of O_2_/·O_2_^–^ and H_2_O_2_/·OH (Figure 17a). Moreover, the presence of OVs promoted the separation of electrons and holes, with electrons being captured by H_2_O_2_ and O_2_ to augment ·OH and ·O_2_^–^ generation based on peroxidase and oxidase-like activities, respectively. Furthermore, the rapid movement of electrons enabled an exceptional sono-thermal effect, resulting in a swift temperature increase to nearly 65 °C after US irradiation. Therefore, the defect-rich BiO_2*–x*_ achieved a multimode synergistic cancer treatment of piezocatalytic, enzymatic, and sono-thermal therapies, significantly inducing cancer cell apoptosis and tumor ablation (Figure 17b,c).

**Figure 17:**
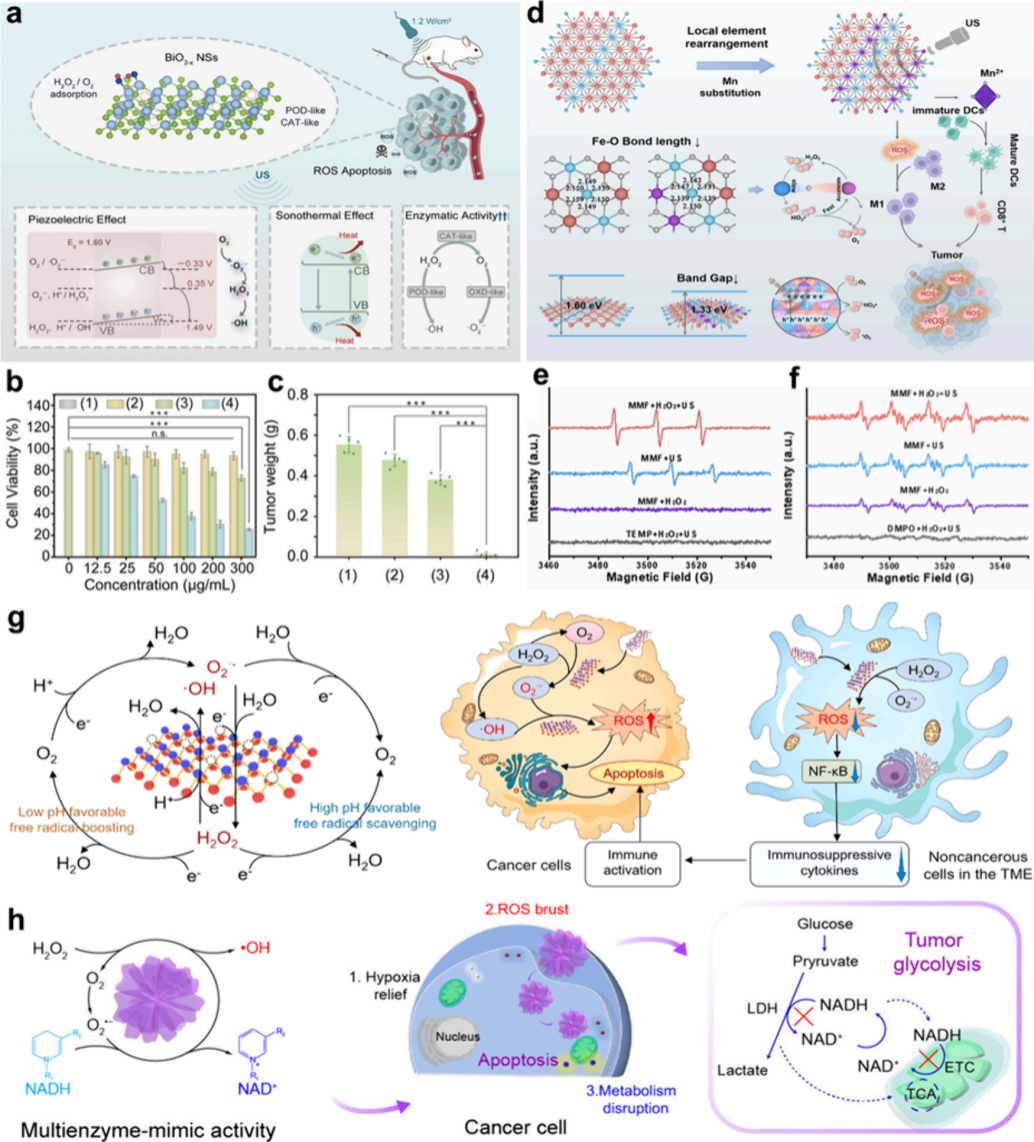
(a) Schematic mechanism of BiO_2–*x*_ nanosheets-mediated multimodal treatment of tumors. (b) Cytotoxicity of 4T1 cells after different treatments. (c) Tumor weight of mice at 14th day after different treatments: (1) control, (2) US, (3) BiO_2–*x*_ nanosheets, and (4) BiO_2–*x*_ nanosheets + US. Reproduced with permission from ref 52. Copyright 2023 John Wiley and Sons. (d) Schematic illustration of MMF nanosheets for efficient SDT combined with immunotherapy. ESR spectra detecting the generation of (e) ^1^O_2_ and (f) ·O_2_^–^ at different conditions. Reproduced with permission from ref 286. Copyright 2023 Elsevier. (g) Schematic illustration of the catalytic activity of OV-rich BiO_2–*x*_ nanosheets. Reproduced with permission from ref 195. Copyright 2021 John Wiley and Sons. (h) Schematic diagram of Fe-MoO_v_-induced antitumor mechanism. Reproduced with permission from ref 274. Copyright 2021 American Chemical Society.

Aside from defect engineering, heteroatom doping has also demonstrated exceptional capabilities in customizing or optimizing SDT performance of layered nanomaterial-based sonosensitizers. Wang et al. proposed a Mn-based LDH sonosensitizer (Mn-doped MgFe-LDH (MMF)) via metal doping strategy.^286^ Compared with undoped MgFe-LDH, the partial substitution of Mg^2+^ ions by Mn^2+^ ions altered the bond length of Fe–O and narrowed the band gap of MMF (Figure 17d), thereby improving the catalytic performance for O_2_ production (∼3 times) and strengthening the SDT effect under US irradiation (Figure 17e,f). Moreover, the doped Mn^2+^ could effectively promote DCs maturation, induce macrophage polarization, and regulate the immunosuppressive microenvironment. Consequently, MMF achieved synergistic sonodynamic-immunotherapy by killing cancer cells, ablating tumors and inhibiting tumor metastasis. Lei et al. synthesized Fe-doped VS_2_ nanosheets (Fe-VS_2_) for enhanced SDT.^278^ Thanks to the prolonged e^–^–h^+^ recombination time mediated by Fe doping, the ROS generation performance of Fe-VS_2_ nanosheets was greatly boosted under US irradiation in comparison with pristine VS_2_ nanosheets. Moreover, the multivalent V and Fe elements in Fe-VS_2_ nanosheets consumed GSH to amplify the ROS-induced oxidative stress. In vitro and in vivo assays demonstrated the excellent therapeutic effect of Fe-VS_2_ nanosheets on killing cancer cells and ablating tumors under US irradiation. Similarly, Ding et al. also proposed a piezoelectric sonosensitizer (BWO-Fe nanosheets) through metal doping engineering.^275^ Fe doping not only introduced oxygen defects into BWO-Fe, but also endowed it with Fenton activity. The defect-mediated inhibition of e^–^–h^+^ recombination, Fenton activity-mediated ·OH generation, and piezoelectric potential-facilitated e^–^–h^+^ separation synergistically realized enhanced SDT to suppress tumor growth.

Additionally, Hu et al. successfully fabricated amorphous CoW-LDH nanosheets via acid etching-mediated crystalline-to-amorphous phase transformation strategy as highly efficient sonosensitizers for SDT.^57^ Owing to the defects generation and electronic structure changes caused by phase transformation, amorphous CoW-LDH nanosheets exerted superior ROS generation performance when subjected to US irradiation, whose activity was ≈17 times that of the commercial TiO_2_ sonosensitizer, leading to in vitro cell death and in vivo tumor eradication. Similarly, we also reported the preparation of amorphous 2D Mn-doped CoMo-LDH nanosheets by the same phase transformation strategy as a high-efficiency sonosensitizer for MRI-guided SDT.^437^ The doped Mn^4+^ not only decomposed H_2_O_2_ into O_2_ to alleviate tumor hypoxia, but also consumed GSH to prevent ROS clearance, synergistically promoting ROS generation upon US irradiation.

In addition to strengthening the SDT performance of layered nanomaterials by introducing appropriate surface defects in the above strategies, the ROS generation activity of layered nanomaterials can also be optimized by reducing bulk defects that can act as recombination centers for charge carriers. As a typical example, Yang et al. designed PHI nanosheets (defect-repaired g-C_3_N_4_ nanosheets with a poly heptazine imide structure) for enhanced SDT.^245^ By virtue of the highly delocalized π-conjugated structure, the prepared PHI nanosheets with few bulk defects facilitated rapid charge migration and exhibited high ROS generation activity when exposed to US irradiation, which was stronger than PCN with numerous bulk defects. In vitro and in vivo assays confirmed the effectiveness of the designed PHI nanosheets in killing tumor cells under US irradiation.

#### 6.3.4 Catalytic Therapy/Chemodynamic Therapy

Catalytic therapy has proven to be one of the most appealing cancer treatment strategies, with the advantages of minimize the side effects, no need for the external stimulus, and TME-responsive capability.^438,439^ Catalytic therapy is usually based on the multienzyme activities (e.g., SOD, CAT, POD, and OXD, etc.) of nanozymes to generate ROS (e.g., H_2_O_2_, ·OH, ·O_2_^–^) for killing tumor cells.^440^ Similar to POD activity, CDT, as an emerging noninvasive and tumor-specific therapeutic approach, relies on Fenton or Fenton-like reactions initiated by metal ions (such as Fe^2+^, Cu^+^, Co^2+^, Ni^2+^, Mn^2+^, and Ti^3+^) to catalyze the conversion of overexpressed H_2_O_2_ in the TME into highly toxic ·OH, thereby specifically inducing tumor cell apoptosis.^441–444^ In recent years, a variety of nanozymes or Fenton reagents have been successfully constructed by regulating the composition and structure of layered nanomaterials at the atomic level using structural engineering strategies, especially defect engineering, heteroatom doping and interlayer engineering.

Defects have been demonstrated to play an important role in dominating multienzyme activities by providing abundant reactive active sites and efficient electronic orbitals that facilitate energy transfer.^48,50^ In view of this, various defect engineering strategies have been employed to activate the enzyme activities of layered nanomaterials. Yuan et al. first proposed OV-rich BiO_2*–x*_ nanosheets obtained by sonication-assisted liquid-phase exfoliation as a radical regulator for environment-adaptive catalytic therapy.^195^ Specifically, OV-rich BiO_2*–x*_ nanosheets could generate toxic free radicals ·OH and ·O_2_^–^ by virtue of their OV-dominated multienzyme activities (SOD, CAT, POD, and OXD). In vitro and in vivo assays indicated that OV-rich BiO_2*–x*_ nanosheets exerted effective antitumor effects by eliciting tumor cell apoptosis and enhancing T-cell infiltration (Figure 17g). Jiang et al. reported a defect engineering strategy that combined HF exfoliation and H_2_O_2_ oxidation to fabricate TiO_*x*_@C with abundant surface defects as a Fenton-like catalyst for CDT.^199^ The existence of mixed-valence Ti^δ+^ (δ = 4, 3, 2, 0) within TiO_*x*_@C significantly promoted ROS generation, resulting in stable and superior Fenton-like catalytic activity. Given that heat can accelerate the Fenton reaction rate, Zhu et al. constructed sulfur-deficient engineered biodegradable cobalt sulfide quantum dots (CoS_*x*_ QDs) for hyperthermal-enhanced CDT.^445^ The defect-engineered CoS_*x*_ QDs exhibited exceptional photothermal performance when subjected to 808 nm laser irradiation, which further enhanced the Fenton reaction rate and catalyzed endogenous H_2_O_2_ to generate harmful ROS, thus leading to effective ablation of cancer cells.

Aside from defect engineering, heteroatom doping has also been utilized to design nanozymes or Fenton reagents by introducing Fenton catalytic active ions, creating active sites, or generating defects. For example, the defective Cu-doped MgAl-LDH proposed by Sun et al. was constructed through ion exchange and acid etching for CDT.^258^ The introduction of Cu^2+^ ions endowed MgAl-LDH with Fenton catalytic activity, which was further enhanced by GSH-mediated Cu^2+^ reduction and laser-induced temperature rise. Consequently, Cu-doped MgAl-LDH significantly inhibited 4T1 cell proliferation and tumor growth. Jiao et al. synthesized FeSNC SACs (Fe single atoms on S/N codoped carbon) through calcination-mediated heteroatom doping engineering for catalytic therapy.^288^ It was found that FeSNC characterized by unsymmetrically coordinated Fe–N_3_S_1_ sites exhibited much higher POD-like activity in comparison with FeNC containing Fe–N_4_ sites. This enhancement could be attributed to the S doping-induced electronic and geometric effects, which lengthened the O–O bond distance of adsorbed H_2_O_2_, accelerated the electronic transfer between O and Fe, and reduced the energy barrier for forming active intermediate, thereby boosting POD-like activity. Wu et al. synthesized a Fe–N doped graphene (FeNGR) that could mimic the activity of NADPH oxidase to catalyze the conversion of NADPH into NADP^+^ and trigger oxygen radical generation.^446^ The same group further discovered that Co–N and Cu–N doped graphene also displayed strong NADPH oxidase activity.^287^

Yu et al. designed a Fe-MoO_v_ nanozyme using doping strategy, which possessed reinforced enzyme-mimicking activities (CAT, OXD, POD, and NADH oxidase) and photothermal performance due to Fe doping-facilitated structural reconstruction with abundant OVs and Fe substitution (Figure 17h).^274^ Under NIR-II (l064 nm) laser irradiation, Fe-MoO_v_ nanozyme effectively induced substantial destruction of REDOX and metabolic homeostasis in the tumor region, achieving excellent photoenhanced catalytic therapeutic effects. Most recently, Cheng et al. developed Fe-doped VO_*x*_ (Fe-VO_*x*_) nanozymes for osteoradionecrosis therapy.^447^ Fe doping-induced OVs generation and Fe substitution substantially augmented triple-enzyme-mimicking activities (CAT, SOD, and glutathione peroxidase (GPx)), effectively remodeling the jawbone microenvironment, restoring mitochondrial integrity through amplified mitophagy. Moreover, Fe-VO_*x*_ exhibited pronounced surface plasmon resonance, enabling potent photothermal action against jaw infections. Collectively, Fe-VO_*x*_ nanozymes demonstrated comprehensive reactive oxygen/nitrogen species scavenging, robust mitophagy activation and significant antibacterial efficacy, establishing their therapeutic potential for osteoradionecrosis intervention.

In addition to defect engineering and heteroatom doping, interlayer engineering can also activate the enzyme activities of layered nanomaterials by inducing defect generation and valence change of active metal ions. Zhou et al. reported the activation of layered MoO_3_ nanobelts through interlayer engineering as a promising nanozyme for PTT-enhanced catalytic therapy.^53^ The obtained NH-MoO_3*–x*_ nanobelts cointercalated with Na^+^ and H_2_O exhibited exceptional enzyme-mimicking catalytic activity for ROS generation in comparison with the pristine MoO_3_ nanobelts, which could be credited to the intercalation-induced defect generation, partial reduction of Mo^6+^, and interlayer spacing expansion. Moreover, the enhanced absorption of NH-MoO_3*–x*_ nanobelts in the NIR-II region significantly promoted their enzymatic activity via 1064 nm laser-induced photothermal effect. After modification with BSA, NH-MoO_3*–x*_ nanobelts efficiently killed cancer cells and eliminated tumors upon 1064 nm laser irradiation. Similarly, Hu et al. successfully fabricated PANI- or dye molecule-intercalated MoO_3*–x*_ organic/inorganic superlattices using the above Na^+^/H_2_O cointercalation strategy combined with ion exchange method.^62,63^ The intercalation of PANI with good conductivity could facilitate electron transport during the MoO_3*–x*_-mediated Fenton-like reaction, thus achieving highly efficient CDT to eliminate cancer cells and ablate tumors. The intercalation of fluorescence dye molecules such as NB, TPA-I, TPE-I, and PHC-I endowed MoO_3–*x*_ with the fluorescence imaging capability, enabling imaging-guided catalytic therapy to efficiently induce cancer cell death and completely eradicate tumors.

#### 6.3.5 Immunotherapy

Immunotherapy, which strategically activates the immune system, has emerged as the fourth pillar of cancer treatment alongside conventional modalities including surgery, radiotherapy, and chemotherapy.^448,449^ This therapeutic paradigm has achieved remarkable breakthroughs in clinical management of diverse malignancies.^450–453^ Central to its efficacy are immunoadjuvants, which are typically incorporated to amplify antigen immunogenicity and activate antigen-presenting cells.^454–456^ Surprisingly, advanced structural engineering strategies, especially crystal phase engineering, defect engineering and heteroatom doping, provide guarantees for the structural design, material synthesis, and performance enhancement of layered nanomaterial-based immunoadjuvants.

As a typical example, Huang et al. developed 1T-phase MoSe_2_ nanosheets with abundant defects through Li intercalation-mediated crystal phase engineering for enhanced NIR-II photothermal immunotherapy.^173^ 1T-phase MoSe_2_ nanosheets not only exhibited evidently enhanced NIR-II photothermal performance as compared to 2H-phase MoSe_2_, but also amplified oxidative stress by depleting GSH duo to the rich exposed active Mo centers. The synergistic effect of MoSe_2_-mediated high-performance PTT and efficient GSH consumption promoted the release of tumor-associated antigens, thereby significantly inducing robust ICD and activating systemic immune response to suppress tumor cell growth and metastasis. Similarly, Zhu et al. reported biodegradable metallic 1T-phase MoS_2_ nanosheets prepared by a one-pot hydrothermal method for synergistic NIR-II photothermal immunotherapy.^457^ MoS_2_ nanosheets not only showcased exceptional NIR-II photothermal performance with a high PCE of 56%, but also displayed stimulus-responsive biodegradation in H_2_O_2_ environments. When applied to the primary tumor site and exposed to NIR-II light, MoS_2_ nanosheets generated a potent photothermal effect, directly ablating the tumor mass while simultaneously triggering ICD in cancer cells. The ICD cascade promoted dendritic cell maturation and primed T cells. Consequently, MoS_2_-mediated NIR-II photothermal immunotherapy elicited a robust systemic immune response, effectively suppressing growth in both the primary tumor and untreated distant metastases in murine models.

Apart from crystal phase engineering, the introduction of defects has also been demonstrated to confer immunological activity on layered nanomaterials. Du et al. prepared CBNO-OV1 nanosheets for synergistic necroptosis and immunotherapy (Figure 18a).^243^ The piezocatalytic efficiency of CBNO-OV1 nanosheets was optimized by modulating the OV concentration using glyoxal. Compared with the CBNO nanosheets without OVs, the CBNO-OV1 nanosheets exhibited much higher ROS generation performance under US irradiation. ROS burst and Ca^2+^ inward flow stimulated RIPK and MLKL activity, thus inducing necroptosis (Figure 18b,c), which further promoted dendritic cell maturation, induced macrophage polarization, activated toxic T cells, and ultimately enhanced immunotherapy. Similarly, Wang et al. synthesized oxygen-deficient MoO_*x*_ nanoparticles by high-temperature thermal decomposition method as a novel nanosensitizer for US-enhanced cancer metalloimmunotherapy.^458^ MoO_*x*_ nanoparticles not only exhibited effective ROS generation activity when exposed to US irradiation due to their OV structure, but also eliminated GSH to attenuate the antioxidant capacity of solid tumor, synergistically elevating ROS levels to induce cancer cell damage. Moreover, MoO_*x*_ nanoparticles could further stimulate dendritic cells maturation mediated by the ICD process and activate the cGAS-STING pathway based on Mo ions. Therefore, MoO_*x*_-mediated SDT combined with aCTLA-4 achieved a significant enhancement in antitumor therapeutic effect, effectively inhibiting cancer metastases and tumor growth by triggering immune responses.

**Figure 18:**
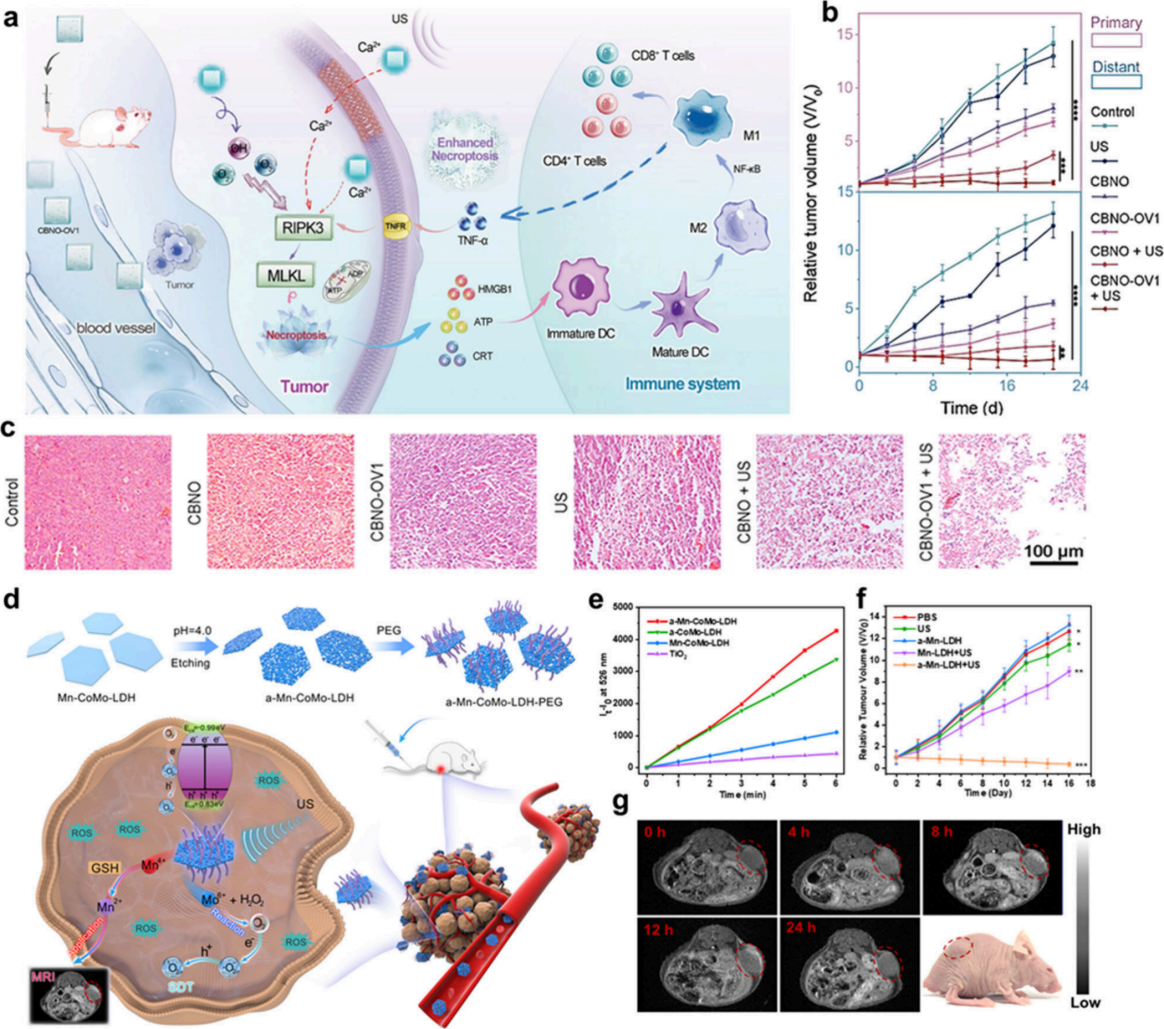
(a) Schematic diagram of CBNO-OV1 nanosheets activating immune response and inducing necroptosis through immunotherapy. (b) Relative tumor (primary and distant) volume of mice after different treatments. (c) H&E staining of primary tumor sections of CT26 cell bearing mice. Reproduced with permission from ref 243. Copyright 2024 John Wiley and Sons. (d) Schematic illustration of the synthesis of a-Mn-CoMo-LDH-PEG and its application for MRI-guided SDT. (e) Fluorescence intensity of SOSG in the presence of Mn-CoMo-LDH, a-Mn-CoMo-LDH, a-CoMo-LDH, and TiO_2_ under US irradiation. (f) Relative tumor growth curve of mice with different treatments. (g) T_1_-weighted MRI of mice after intravenous injection of a-Mn-CoMo-LDH-PEG. Reproduced with permission from ref 437. Copyright 2024 Elsevier.

In addition, heteroatom doping is the most widely used strategy for engineering layered nanomaterials as immunoadjuvants, where the doped heteroatoms can bring about metal immune activity, induce ICD, ferroptosis and cuproptosis, and activate the cGas-STING signaling pathway. For example, Zhang et al. reported Zn^2+^-doped MgAl-LDH (Zn-LDH) as an immunomodulating adjuvant for cancer metalloimmunotherapy.^262^ The Zn–OH bond in Zn-LDH was readily cleaved through hydrolysis in the acidic TME to release abundant Zn^2+^ and elevate the pH of tumor tissues, thereby reversing the immunosuppressive TME and stimulating the activation of tumor-resident immune cells including M1 macrophages, natural-killer cells and cytotoxic T cells. After internalization by tumor cells, Zn-LDH effectively caused mitochondrial damage and disrupted endosomes/lysosomes to inhibit autophagy. Moreover, the released Zn^2+^ activated the cGas-STING signaling pathway, thereby inducing ICD. Owing to the synergistic effect of neutralizing tumor acidity, blocking autophagy and supplementing Zn^2+^, Zn-LDH effectively provoked robust antitumor immunity and effectively inhibited the growth or metastasis of breast cancer and melanoma in mice.

Jana et al. prepared a high-efficiency nanozyme (CMO-R@4T1) by doing Cu into MoO_*x*_ using a hydrothermal method and then coating it with 4T1 cell membranes.^273^ The obtained CMO-R@4T1 nanozyme exhibited multienzyme-mimicking (POD, OXD, and NADH oxidase) activities to generate ROS. Moreover, Cu doping engineering led to a strong NIR-II absorption of CMO-R@4T1 due to the intensified electron delocalization, giving rise to significant photothermal effect under 1064 nm laser irradiation. In combination, CMO-R@4T1 triggered heightened cellular apoptosis by virtue of ROS and hyperthermia, and further induced ICD and dendritic cells maturation, ultimately achieving targeted photoimmunotherapy of tumors.

Aside from hyperthermia-enhanced immunotherapy, Wu et al. synthesized Ca-doped MgFe-LDH (MCF) by a coprecipitation approach for SDT-enhanced cancer immunotherapy.^170^ The doping of Ca^2+^ not only successfully modulated the energy band of MCF and efficiently promoted e^–^–h^+^ separation under US irradiation to boost ROS generation, but also provoked an immune effect through Ca^2+^- and Mg^2+^-mediated polarization of M1 macrophages and activation of CD8^+^ T cells. Moreover, Fe^3+^-mediated GSH consumption and Fe^2+^-mediated Fenton catalytic activity and ferroptosis allowed MCF to overcome immunosuppressive TME, thereby heightening oxidative stress. Eventually, MCF exerted potent antitumor effect to induce tumor ablation through a synergistic effect between superior immune regulation and oxidative stress. Similarly, Geng et al. proposed Pd single atom-doped Ti_3*–x*_C_2_T_*y*_ nanosheets as a rich-defect sonosensitizer for synergistic US-induced SDT/ROS-mediated immunotherapy.^299^

Additionally, Liu et al. synthesized Mn-doped LDH nanosheets using a facile bottom-up method with the aid of H_2_O for ferroptosis and immunotherapy.^459^ The Mn doping significantly contributed to REDOX reaction-mediated GSH consumption and Fenton-like activity-mediated ·OH generation. After loading with pro-inflammatory cytokine interferon (IFNγ), the obtained IFNγ/uMn-LDHs not only impeded GSH synthesis by disrupting the expression of SLC7A11 caused by IFNγ accumulation, but also reinforced the ferroptosis and systemic immunity to induce ICD. Moreover, cGAS-STING pathway activation induced by Mn^2+^ and the damage-associated molecular patterns (DAMPs) released from the ferroptotic tumor cells synergistically boosted dendritic cells maturation and cytotoxic immune cell activation. Taken together, IFNγ/uMn-LDHs achieved highly effective ferroptosis and CDT-enhanced immunotherapy, as evidenced by in vitro and in vivo assays. Gong et al. also reported FeWO_X_ nanosheets as cascade bioreactors for CDT-enhanced immunotherapy by generating ·OH and activating the immune system.^229^

Most recently, Kaur et al. fabricated Sb nanosheets via sonication-assisted exfoliation followed by noncovalent functionalization with β-cyclodextrin (β-CD).^460^ They examined the effects of human myeloperoxidase and plant peroxidase on the biodegradability of both pristine and functionalized Sb nanosheets in the presence of H_2_O_2_. The findings indicated that the β-CD surface coating conferred reduced biodegradability to functionalized Sb nanosheets. Moreover, neither pristine/functionalized Sb nanosheets nor their degradation byproducts exhibited significant cytotoxicity toward THP1 cells. Furthermore, immunomodulatory assessment of THP1 cells confirmed no significant induction of TNF-α production by Sb nanosheets before or after partial degradation, demonstrating their negligible role in immune activation. These findings provide deeper insights into the biodegradation ability of Sb nanosheets and their potential biomedical applications.

#### 6.3.6 Summary

In conclusion, structural engineering strategies enable precise structural regulation and performance optimization of layered nanomaterials across diverse cancer therapeutic methods. Fundamentally, these engineered nanomaterial-based systems demonstrate unique therapeutic advantages, including superior biocompatibility, composition-structure controllability, and facile integration of multifunctional components, all critical for efficient oncological interventions. However, existing research remains predominantly concentrated on the performance enhancement of layered nanomaterials mediated by structural engineering strategies. To bridge the translational gap, comprehensive investigations into their in vivo toxicological profiles, drug delivery kinetics, mechanistic interactions at cellular/subcellular levels, and systemic metabolic pathways must be prioritized. Addressing these critical aspects will be pivotal for advancing the clinical translation of layered nanomaterials and unlocking their full potential in next-generation biomedical applications.

### 6.4 Theranostics

As an innovative advancement in medical science, theranostics that integrates diagnostic and therapeutic functions into a single platform, has shown significant potential in personalized medicine by enabling real-time monitoring of treatment process and outcomes.^461–465^ With the rapid evolution of imaging technologies and cancer therapies, a variety of layered nanomaterials capable of achieving theranostic functions have been developed in recent years through structural engineering strategies, e.g., crystal phase engineering, defect engineering, heteroatom doping, crystalline-to-amorphous phase engineering, etc. Examples of such theranostic applications include MRI-guided PTT, MRI-guided PTT/chemotherapy, MRI-guided PTT/CDT, PAI-guided PTT/CDT, NIR-II fluorescence imaging-guided PTT, MRI or US imaging-guided SDT, CT/PAI-guided SDT/NIR-II phototherapy, etc. These innovations highlight the transformative potential of layered nanomaterials in advancing precision medicine.

Since Gd^3+^-based contrast agents are the most prevailing and primary choice in clinical MRI,^466^ structural engineering strategies have been employed to optimize key parameters to achieve high relaxation and enhanced imaging performance. For instance, Ni et al. fabricated oxygen-deficient Gd^3+^-doped Na_*x*_WO_3_ (Na_*x*_GdWO_3_) nanorods through hydrothermal-mediated defect engineering for MRI-guided PTT.^467^ The presence of OVs largely accelerated proton relaxation of Na_*x*_GdWO_3_ nanorods, resulting in a remarkable relaxivity up to 80 mM^–1^ s^–1^ at 0.7 T and a high relaxivity of 32.1 mM^–1^ s^–1^ at clinical 3.0 T for MRI. Moreover, OVs and/or free electron-induced small polarons conferred Na_*x*_GdWO_3_ nanorods significant photothermal performance under 980 nm laser irradiation, thus achieving MRI-guided PTT.

Based on the role of OVs in promoting photothermal performance, Li et al. also reported a theranostic nanoplatform by engineering Cu-doped MgAl-LDH (Cu-LDH) via acid etching.^205^ The etched Cu-LDH possessed a considerable number of defects around Cu cations, which enabled an enhanced photothermal performance under 808 nm laser irradiation. Moreover, the peculiar microstructure of the doped Cu cations endowed LDH with pH-ultrasensitive T_1_-weighted MRI capacity due to the temporarily formed “H_2_O” around paramagnetic Cu^2+^ center under acidic environments. Consequently, after loading with 5-fluorouracil (an anticancer drug), Cu-LDH achieved pH-sensitive T_1_-weighted MRI, acid-enhanced PTT and heat-facilitated chemotherapy.

Wu et al. designed and synthesized a novel multifunctional Fe­(II)-Ti_3_C_2_ nanoshell as a multimodal PTT/CDT/MRI nanoplatform for theranostic.^300^ Specifically, Fe­(II)-Ti_3_C_2_ exhibited superior photothermal performance than undoped Ti_3_C_2_ nanosheets due to the enhanced conductivity promoted by interlaminar Fe^2+^ ions. Moreover, Fe^2+^-mediated Fenton reaction endowed Fe­(II)-Ti_3_C_2_ with the ability to generate ·OH for CDT. In addition, Fe­(II)-Ti_3_C_2_ could function as a MRI contrast agent attributing to the paramagnetic properties of unpaired electron in Fe^2+^. In vitro and in vivo assays revealed that Fe­(II)-Ti_3_C_2_ effectively induced cancer cell apoptosis and tumor ablation under 808 nm laser irradiation, achieving MRI-guided PTT/CDT synergistic therapy. Similarly, Zhu et al. constructed ultrathin Fe-doped CoMn dichalcogenide nanosheets (CFMS) by sulfuration of Fe-doped CoMn-LDH for PAI-guided PTT/CDT.^468^ CFMS nanosheets containing CoS_2_/FeS_2_ crystal phase inherited the dispersed metal atom arrangement, large specific surface area, ultrathin plate-like morphology, and particle size of LDH precursor. More importantly, the sulfuration process activated the NIR-I absorbance ability of CoFeMn-LDH precursor, resulting in a high PCE of 89.0% for CFMS nanosheets to induce hyperthermia and achieve PAI under 808 nm laser irradiation. With the aid of Fe^2+^-mediated Fenton reaction, CFMS nanosheets played a significant role in cancer cells apoptosis and tumor elimination by PAI-guided PTT/CDT.

Recently, Wang et al. presented N and B dual-doped GODs (N-B-GODs) for NIR-II fluorescence imaging-guided PTT.^424^ N and B atoms doping could cause significant local electron energy distortion and generate additional energy gaps, while vacancy defects in N-B-GQDs could red-shift the emission peak to the NIR-II window. The synergistic effect of atom doping and defects endowed N-B-GQDs with NIR-II fluorescence imaging capability. Moreover, N-B-GQDs efficiently absorbed NIR light and converted it into heat under 808 nm laser irradiation, thereby exerting a PTT-mediated therapeutic effect to kill cancer cells and inhibit tumor growth. Similarly, Huang et al. develop Er-doped WSe_2_ nanosheets for NIR-II bioimaging and PTT.^272^ Thanks to the radiative transition (from ^4^I_13/2_ state to the ground state ^4^I_15/2_) of photons emitted by Er ions, Er-doped WSe_2_ nanosheets exhibited typical luminescence at 1542 nm, a characteristic absent in pure WSe_2_ nanosheets. The NIR II emission from Er ions demonstrated significant potential for deep-tissue optical imaging owing to its superior biopenetration capacity. Moreover, Er-doped WSe_2_ nanosheets possessed high PCE (35.2%) under 808 nm laser irradiation, showing great potential in photothermal imaging and PTT.

Apart from PTT excited by light in the NIR-I window (e.g., 808 and 980 nm laser), crystal phase engineering has been demonstrated to activate the NIR-II absorbance ability of layered nanomaterials for imaging-guided NIR-II PTT. Zhou et al. successfully prepared ultrasmall single-layer 1T-MoS_2_ nanodots from microsized 2H-phase MoS_2_ crystals via Li-intercalation method and ultrasmall single-layer 2H-MoS_2_ nanodots from 1T-MoS_2_ nanodots through hydrothermal method, aiming to reveal the impact of the crystal phase of MoS_2_ on its PAI and NIR-II PTT performance.^59^ It was found that the 1T-MoS_2_ nanodots possessed a larger PCE (43.3%) and extinction coefficient (25.6 L g^–1^ cm^–1^) at 1064 nm compared with the 2H-MoS_2_ nanodots with a PCE of 21.3% and an extinction coefficient of 5.3 L g^–1^ cm^–1^. Moreover, compared to the negligible PAI signal of 2H-MoS_2_ nanodots, the 1T-MoS_2_ nanodots exhibited stronger PAI signal in the NIR-II window, showing more promising imaging contrast potential. The superior photothermal properties of 1T-MoS_2_ nanodots than 2H-MoS_2_ (semiconducting phase) could be attributed to metallic nature originated from metallic 1T-phase, implying that the crystal phase governs the photothermal performance of TMDs-based nanomaterials in NIR-II PTT guided by PAI.

In addition to imaging-guided NIR-I or NIR-II PTT combined with CDT or chemotherapy, layered nanomaterials have been engineered for imaging-guided SDT. Cui et al. reported the preparation of amorphous Mn-doped CoMo-LDH nanosheets (a-Mn-CoMo-LDH) through acid etching-mediated phase transformation for MRI-guided SDT (Figure 18d).^437^ The a-Mn-CoMo-LDH nanosheets possessed superior ROS generation activity than amorphous CoMo-LDH nanosheets (∼1.3 times) and pristine Mn-CoMo-LDH nanosheets (∼3.9 times) under US irradiation (Figure 18e). The performance enhancement of a-Mn-CoMo-LDH nanosheets could be attributed the synergistic effect of acid etching and metal doping in narrowing the bandgap, introducing defects, and changing energy levels. More importantly, the doping of Mn^4+^ in the a-Mn-CoMo-LDH nanosheets not only alleviated the hypoxia level in the TME by decomposing H_2_O_2_ into O_2_, but also protected ROS from clearance by consuming GSH to generate Mn^2+^, synergistically boosting the SDT performance (Figure 18f). The generation of Mn^2+^ also endowed the a-Mn-CoMo-LDH nanosheets with T_1_-weighted MRI contrast ability (Figure 18g), thus achieving MRI-guided SDT-mediated theranostic. Similarly, Cao et al. proposed amorphous CoBiMn-LDH (a-CoBiMn-LDH) and amorphous CoBiFe-LDH (a-CoBiFe-LDH) nanoparticles as multifunctional sonosensitizers for US imaging-guided SDT due to Mn^4+^-mediated O_2_ generation and T_2_-weighted MRI-guided SDT owing to the paramagnetic properties of Fe ion, respectively.^328,329^

Most recently, Hong et al. proposed an ultrasonic cavitation effect enhanced sonodynamic combined with 1208 nm laser-induced phototherapy strategy based on thermoelectric/piezoelectric bismuth oxychloride nanosheets (BNs) engineered with oxygen defects for CT/PAI-guided tumor treatment.^469^ The oxygen-defect BNs were fabricated through a sequential process involving a Tween-20-assisted hydrothermal reaction and NaBH_4_-mediated reduction reaction. The incorporated OVs significantly amplified the absorption of oxygen-containing molecules, facilitated electron transport, and boosted the conversion efficiency of externally applied energy (1208 nm light and US). This synergistic enhancement drove substantially localized heat generation and ROS production, dramatically improving the performance of both SDT and NIR-II phototherapy under hypoxic conditions. Furthermore, the high-Z element bismuth provided strong X-ray attenuation for CT imaging, while the BNs’ pronounced NIR-II absorption enabled effective PA imaging. In vivo validation demonstrated outstanding therapeutic outcomes with the BNs platform, achieving complete eradication of tumors within a 10-day period without recurrence.

In summary, layered nanomaterials, fine-tuned through structural engineering strategies, hold significant promise for applications in theranostics. However, the current scope of engineered layered nanomaterials is predominantly confined to MRI-guided PTT and SDT, with a relatively limited range of imaging modalities and therapeutic approaches achievable. The integration of multiple imaging techniques and treatment modalities into a singular nanosystem presents an opportunity for the rational design and engineering of layered nanomaterials, facilitating the simultaneous acquisition of comprehensive biological information and the attainment of optimal therapeutic outcomes. Future endeavors should focus on the exploration of new in vivo diagnostic probes based on layered nanomaterials and further synergizing them with other promising therapeutic strategies. This integration, achieved through advanced structural engineering, is essential for realizing superior theranostic efficacy. Moreover, subsequent research should rigorously assess the potential long-term side effects of layered nanomaterials in living organisms and evaluate their clinical viability through comprehensive toxicological analyses. This dual focus will be pivotal in advancing the field and ensuring the safe, effective application of layered nanomaterials in clinical settings.

### 6.5 Biosensing

Biosensing technology serves as a critical platform in modern healthcare, enabling early disease diagnosis, risk prediction, clinical screening of high-risk groups, pharmacological evaluation of therapeutic efficacy and safety.^470^ With the expansion of the application of biosensors in the medical field, the demand for enhanced performance metrics, particularly high sensitivity, good stability, and low cost are becoming increasingly high.^471–473^ The unique properties of engineered layered nanomaterials, e.g., redox, magnetism, alkalinity, acidity, and conductivity, etc., enable them to interact with biomolecules, thereby accelerating interface electron transfer, promoting catalytic reactions on electrode surfaces, and boosting the performance of biosensors.^474–476^ Therefore, biosensors based on layered nanomaterials are of great interest to researchers. Structural engineering strategies such as defect engineering, heteroatom doping, and crystalline-to-amorphous phase engineering, have been instrumental in improving the sensitivity and stability of layered nanomaterial-based biosensors.

For example, Chen et al. constructed defect-rich MoS_2_ QDs via hydrothermal method-mediated defect engineering as a ECL immunosensor for carcinoembryonic antigen (CEA) detection (Figure 19a).^225^ Surface defects in MoS_2_ QDs not only significantly enhanced their fluorescence emission with a quantum yield of 7.7%, but also conferred robust ECL activity using K_2_S_2_O_8_ as a coreactant due to the strong binding affinity of sulfur defects toward SO_4_^•–^, resulting in a maximum emission at ∼810 nm. The proposed MoS_2_ QDs could function as a biosensor for CEA detection with a linear range from 0.005 to 400 ng/mL and a detection limit of 0.005 ng/mL (Figure 19b,c), which demonstrated high selectivity and exhibited significant potential for practical application in clinical serum analysis.

**Figure 19:**
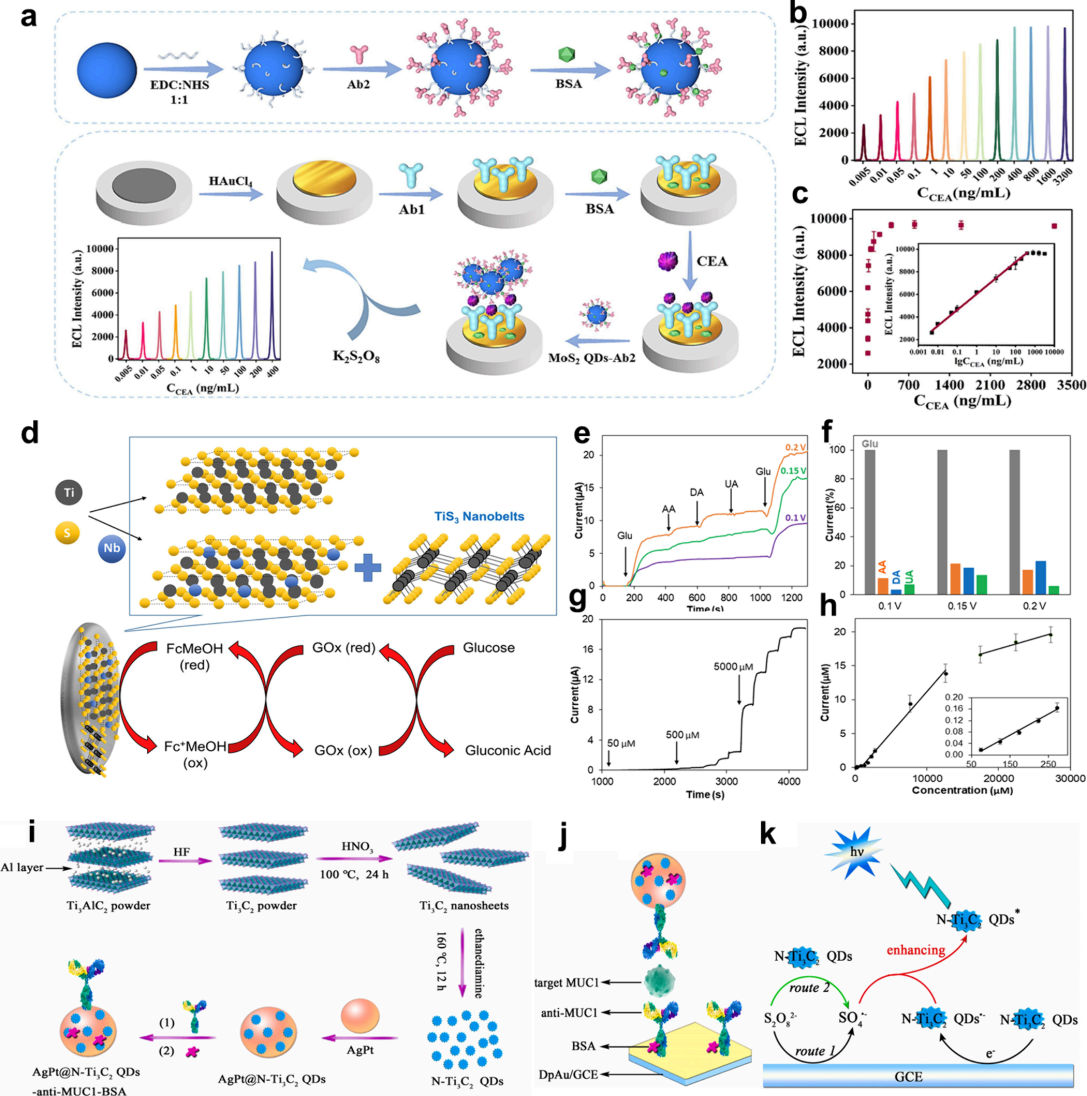
(a) Schematic illustration of the synthesis of MoS_2_ QDs for CEA detection. (b) ECL-time profiles of MoS_2_ QDs-based immunosensor for different CEA concentrations with K_2_S_2_O_8_. (c) The relationship between ECL intensity and CEA concentration. Reproduced with permission from ref 225. Copyright 2023 Elsevier. (d) Schematic diagram of Ti_1–*x*_Nb_*x*_S_2_-based biosensor for glucose detection. (e) Chronoamperometry data after additions of glucose and interferences at different operating potentials. (f) Current signals from interferences as a percentage of glucose. (g) Chronoamperometry data after additions of 50, 500, and 5000 μM glucose. (h) Calibration graph of current against glucose concentration with three linear ranges. Reproduced with permission from ref 289. Copyright 2020 Elsevier. (i) Schematic illustration of the synthesis of N-doped Ti_3_C_2_ QDs, (j) construction of the ECL immunosensor, and (k) its application for sensitive mucin 1 detection. Reproduced with permission from ref 253. Copyright 2022 Elsevier.

Joshi et al. prepared rGO films with adjustable microscopic and macroscopic defect densities using pulsed laser deposition and laser annealing methods for H_2_O_2_ electrochemical sensing.^239^ Pulsed laser led to surface melting of the a-C thin film, thus forming defect-rich rGO with dangling bonds. The presence of dangling bonds and defects (e.g., point defects, holes, and corrugations) could serve as active sites for H_2_O_2_ reduction, improving the detection limit by several orders of magnitude. It was found that the rGO film treated with 0.6 J cm^–2^ and 10 pulses exhibited the best catalytic response toward electrochemical H_2_O_2_ reduction with a detection limit of 7.15 nM and sensitivity of 0.235 μA/mM cm^–2^, holding great potential for nonenzymatic electrochemical sensing.

In addition to introducing a large number of defects to boost biosensing performance, Li et al. proposed GSH-Ti_3_C_2_ QDs through a defect repair strategy as an ECL biosensor for detecting miRNA221 in breast cancer tumor.^198^ The binding of metal atoms on Ti_3_C_2_ to the sulfhydryl group of GSH reduced the defects in the structure, resulting in a single narrow emission peak of GSH-Ti_3_C_2_ QDs and significantly improving their luminescence performance. Compared with Ti_3_C_2_ QDs, GSH-Ti_3_C_2_ QDs exhibited higher ECL stability. The ECL intensities of GSH-Ti_3_C_2_ QDs could maintain 98.7% within 5 days and decreased to 57.1% within 20 days, while the ECL intensities of Ti_3_C_2_ QDs decreased to 20.9% within 20 days. The MXene-based ECL biosensor constructed in this work displayed a good selectivity for miRNA221 detection, with an appropriate linear range (10 fM ∼ 10 nM) and low detection limit (10 fM), showing great potential for clinical analysis.

Aside from the above defect engineering, Wu et al. proposed a highly active S-doped rGO though hydrothermal-mediated heteroatom doping for the detection of H_2_O_2_ and glucose.^250^ It was found that S-rGO exhibited markedly strengthened peroxidase-like activity in comparison with the undoped rGO, primarily mediated by sulfur-derived catalytic sites. Leveraging this optimized nanozyme activity of S-rGO, a sensitive and efficient colorimetric sensing platform was established for dual detection of H_2_O_2_ and glucose, with low detection limits of 0.042 μM and 0.38 μM, and the linear ranges of 0.1–1 μM and 1–100 μM, respectively. Similarly, Kim et al. prepared N- and B-codoped rGO (NB-rGO) as a peroxidase-mimicking nanozyme for C-reactive protein (CRP) and acetylcholine (ACh^+^) bioassays.^266^ Benefiting from the synergistic effect of dual-doped N and B atoms promoting electron transfer, NB-rGO demonstrated higher catalytic efficiency than undoped rGO with 3 orders of magnitude. The enhancement of peroxidase-like activity of NB-rGO could be attributed to the increase in active site density caused by N and B codoping. When used as a biosensor, NB-rGO had higher sensitivity, selectivity, and linearity for ACh^+^ and CRP bioassays than horseradish peroxidase or Pt nanoparticles. The linear range/limit of detection of NB-rGO for detection of ACh^+^ and CRP were 50–5000 nM/30 nM and 1–5000 ng mL^–1^/5 ng mL^–1^, respectively.

Rohaizad et al. adopted niobium-doped TiS_2_ (Ti_1–*x*_Nb_*x*_S_2_) as an enzymatic biosensor for glucose detection (Figure 19d).^289^ The doping of niobium with appropriate concentration (*x* = 0.05) could endow Ti_0.95_Nb_0.05_S_2_ with a large electrochemically active area, less electron transfer resistance, and superior conductivity. When applied to glucose detection, Ti_0.95_Nb_0.05_S_2_ exhibited the highest sensitivity compared to other glucose biosensors based on layered materials, with a wide linear range from μM to mM and a detection limit of 25.7 μM (Figure 19e–h). This was the first case about the electrochemical biosensors reported based on TiS_2_. Similarly, Zhang et al. proposed N-rGO as an electrochemical biosensor for simultaneous detection of dopamine, uric acid and ascorbic acid.^267^ The doping of N enhanced the conductivity and binding ability of N-rGO, making it highly effective in detecting dopamine, uric acid and ascorbic acid, with the linear ranges of 1–60 μM, 1–30 μM, and 0.1–4 mM, and the detection limits of 0.1 μM, 0.2 μM, and 9.6 μM, respectively.

Besides, Yan et al. prepared S and N codoped Nb_2_C MQDs (S, N-MQDs) with high quantum yield by hydrothermal method for cell imaging and Cu^2+^ ion sensing.^255^ The proposed S, N-MQDs demonstrated dual stability (photostability and colloidal dispersion) alongside green-emitting fluorescence with a high quantum yield of 17.25%, serving as a dual-function fluorescent probe for Caco-2 cells labeling while achieving Cu^2+^ ions detection with a detection limit of 2 μmol/L. Jiang et al. utilized N-doped Ti_3_C_2_ QDs to fabricate ECL immunosensor for sensitive mucin 1 detection (Figure 19i–k).^253^ N-doped Ti_3_C_2_ QDs exhibited enhanced ECL quantum efficiency compared with undoped counterparts with a relative ECL quantum efficiency of 1.58, which is attributed to its abundant active catalytic sites and exceptional electronic conductivity, thus achieving sensitive mucin 1 detection with a low detection limit of 0.31 fg mL^–1^. Similarly, Wang et al. and Luo et al. also employed nitrogen-doped Ti_3_C_2_ MXene quantum dots as electrochemical biosensors for ultrasensitive H_2_O_2_ detection and Fe^3+^ detection/GSH fluorescence imaging, respectively.^254,477^

In addition, Liu et al. prepared amorphous MoO_3_ nanosheets through crystalline-to-amorphous phase engineering by combining MoS_2_ oxidation and supercritical CO_2_ treatment as a biosensing system for BSA detection.^325^ The crystal phase transformation of MoO_3_ nanosheets enabled the tunable plasmon resonances in the visible and NIR regions under illumination. In a BSA-based optical biosensing system, the amorphous MoO_3_ nanosheets exhibited superior sensitivity and unique response than crystal MoO_3_ nanosheets. For crystal MoO_3_ nanosheets, the negatively charged BSA was immobilized on their surface and repelled the free electrons, leading to a decrease in the free electron density. In contrast, the BSA preferentially accumulated within defect sites of amorphous MoO_3_ nanosheets, resulting in increased electron density and enhanced plasmon resonance. This disparity in adsorption behavior arises from distinct atomic arrangements (amorphous vs crystalline phases) within 2D MoO_3_ nanosheets, though the precise interfacial dynamics remain to be elucidated.

In general, layered nanomaterials have demonstrated exceptional capabilities in biosensing applications, particularly for the detection of critical biomarkers including CEA, H_2_O_2_, mRNA, and glucose, etc., achieving significant improvements in selectivity, sensitivity and operational stability of biosensors. Despite these advancements, several challenges persist in the field: Material diversity limitation: Current biosensing platforms predominantly utilize rGO and Ti_3_C_2_ MXene, representing only a fraction of available layered nanomaterials. Performance gaps: Detection thresholds for many biomarkers remain above clinically relevant concentrations, and the deposition of reaction intermediates and/or active substances may block working electrode surface to affect specificity. Device accessibility: The lack of cost-effective and portable (hand-held) detection systems limits point-of-care applicability. With the development of biosensors based on engineered layered nanomaterials and the advancement of sensor devices, the detection thresholds of biosensors are projected to attain marked improvements, opening up a new path for clinical analysis of more biomolecules with important functions. These innovations will enable next-generation biosensors capable of multiplexed detection at clinically relevant concentrations, revolutionizing biomarker analysis for precision medicine applications.

### 6.6 Antibacteria

Bacterial infections have emerged as a critical global health challenge, traditionally managed through antibiotic therapies.^478,479^ However, the escalating inefficacy of conventional antibiotics, compounded by the rapid development of microbial resistance mechanisms, has necessitated the exploration of alternative antimicrobial strategies.^480–486^ Layered nanomaterials have emerged as a promising platform for next-generation antimicrobial development, owing to their precisely tunable composition structure and modifiable electronic properties. Through structural engineering strategies (e.g., crystal phase engineering, defect engineering, heteroatom doping, and interlayer engineering), layered nanomaterials can be functionally enhanced to address current antimicrobial limitations. These engineering strategies enable: optimization of intrinsic antibacterial properties of layered nanomaterials through precise control of surface chemistry and electronic structure; integration of multifunctional capabilities for synergistic antimicrobial action; reduction of resistance development through multimodal mechanisms of action; minimization of cytotoxic effects while maintaining potent bactericidal efficacy. Overall, structural engineering represents a paradigm shift in antimicrobial development, leveraging precisely controlled nanoscale physical and chemical interactions for more effective infection treatment.

Chen et al. prepared MoS_2_ nanosheets with different phases (1*T*/1T′-phase via Li-intercalation method or 2H-phase via hydrothermal method) and explored the crystal phase-dependent enhancement mechanisms in photothermal-augmented sonodynamic antibacterial efficacy.^172^ Interestingly, the metallic 1*T*/1T′-phase MoS_2_ nanosheets with abundant defects exhibited superior activity toward US-triggered ROS generation in comparation with the semiconducting 2H-phase MoS_2_ nanosheets. More importantly, thanks to the metallic phase-enabled strong NIR-II absorption, the US-triggered ROS generation activity of 1*T*/1T′-phase MoS_2_ nanosheets was further enhanced under 1064 nm laser irradiation due to the photothermal effect. In vitro assays indicated that 1*T*/1T′-phase MoS_2_ nanosheets exhibited exceedingly effective sterilization performance (∼100%) on *Pseudomonas aeruginosa* (*P. aeruginosa*) and *S. aureus* under both US and laser irradiation. It can be inferred that the crystal phase emerges as a pivotal factor governing the PCE and sonodynamic performance activation in layered nanomaterials. Similarly, Mutalik et al. also demonstrated superior photothermal-mediated antibacterial effect of 1T-MoS_2_ nanosheets compared with the semiconducting 2H-MoS_2_ nanosheets under 808 nm laser irradiation.^176^

Kim et al. also examined the antimicrobial efficacy of 1T-phase MoSe_2_, MoS_2_, and WS_2_ nanosheets synthesized by Li-intercalation method against *E. coli*.^164^ It was found that 1T-phase TMD nanomaterials exhibited excellent antibacterial activity to kill *E. coli* by generating ROS, oxidating GSH, and inducing cellular membrane destabilization and cytoplasmic constituent efflux through direct interfacial interactions with bacterial surfaces, which was correlated with the levels of physical membrane stress, oxidative stress, electrical conductivity, and charge transfer feasibility. Consequently, 1T-phase MoSe_2_, MoS_2_, and WS_2_ nanosheets realized sterilization rates of 46.3%, 55.2%, and 65.6%, respectively. Similarly, Basu et al. synthesized 1T-phase MoS_2_ nanosheets from 2H-phase MoS_2_ nanosheets through atomic doping-mediated crystal phase engineering for antibacteria.^167^ 1T-phase MoS_2_ nanosheets demonstrated the commendable antimicrobial activity against *Alternaria alternata* by generating ROS, which could be attributed to the introduction of defects in the lattice structure of 1T-phase MoS_2_ nanosheets promoting the sorption of O_2_ and subsequent conversion into ROS.

In addition to the crystal phase, the effect of the layer number (thickness) of MoS_2_ nanosheets on the antibacterial function has also been explored. Kaur et al. prepared few-layered MoS_2_ nanosheets through direct exfoliation in pure water and evaluated its antibacterial action against two types of *Salmonellas*.^163^ It was found that MoS_2_ nanosheets with 2–5 layers exhibited significant bactericidal effects by inducing membranes mechanical injury and oxidative stress, which may be due to the fact that MoS_2_ nanosheets could serve as nanoblades to cut the external cell wall of *Salmonella* as its thickness is less than or approximately the same as that of the walls.

Apart from crystal phase engineering, defect engineering has also been shown to confer antimicrobial activity on layered nanomaterials. Sun et al. constructed oxygen-rich vacancies BBR for NIR light-driven antibacteria.^218^ The results indicated that BBR possessed much stronger antibacterial activity than oxygen-poor vacancies BBP in killing *B. subtilis* and *E. coli* when exposed to 808 nm laser irradiation. The enhancement of antimicrobial efficacy could be attributed to the rich OVs on BBR, which enabled stronger light absorption capacity, longer charge carrier lifetime, better adsorption of O_2_, more intimate interactions with bacteria, and more ROS generation, thus achieving efficient photocatalytic antibacterial effects. Similarly, Ma et al. designed BiOI-DE nanosheets with oxygen defects and Bi decoration using NaBH_4_ reduction approach.^216^ Thanks to the presence of oxygen defects and semimetal bismuth, as well as changes in band structure and electron distribution, BiOI-DE nanosheets exhibited high PCE and strong photocatalytic performance, thus employing hyperthermia and abundant ROS to kill *E. coli* under 808 nm laser irradiation, with a sterilization efficiency of 99%. Additionally, Mao et al. fabricated Ti_3_C_2_[Ti_3_C_2_-SD­(Ti^3+^)] nanosheets containing abundant Ti^3+^ species by acid etching strategy.^208^ The generated slip dislocations with Ti^3+^ species could reduce the O_2_ activation energy barrier and form surface-bound O because of the activation of O_2_ under US irradiation, thereby leading to a substantial amount of ^1^O_2_ generation through the REDOX reaction between O_2_ and electrons (Figure 20a). Consequently, Ti_3_C_2_[Ti_3_C_2_-SD­(Ti^3+^)] demonstrated superior therapeutic efficacy in bony tissue infection in comparison with traditional antibiotics (Figure 20b,c), with a bactericidal ability of 99.72% ± 0.03%.

**Figure 20:**
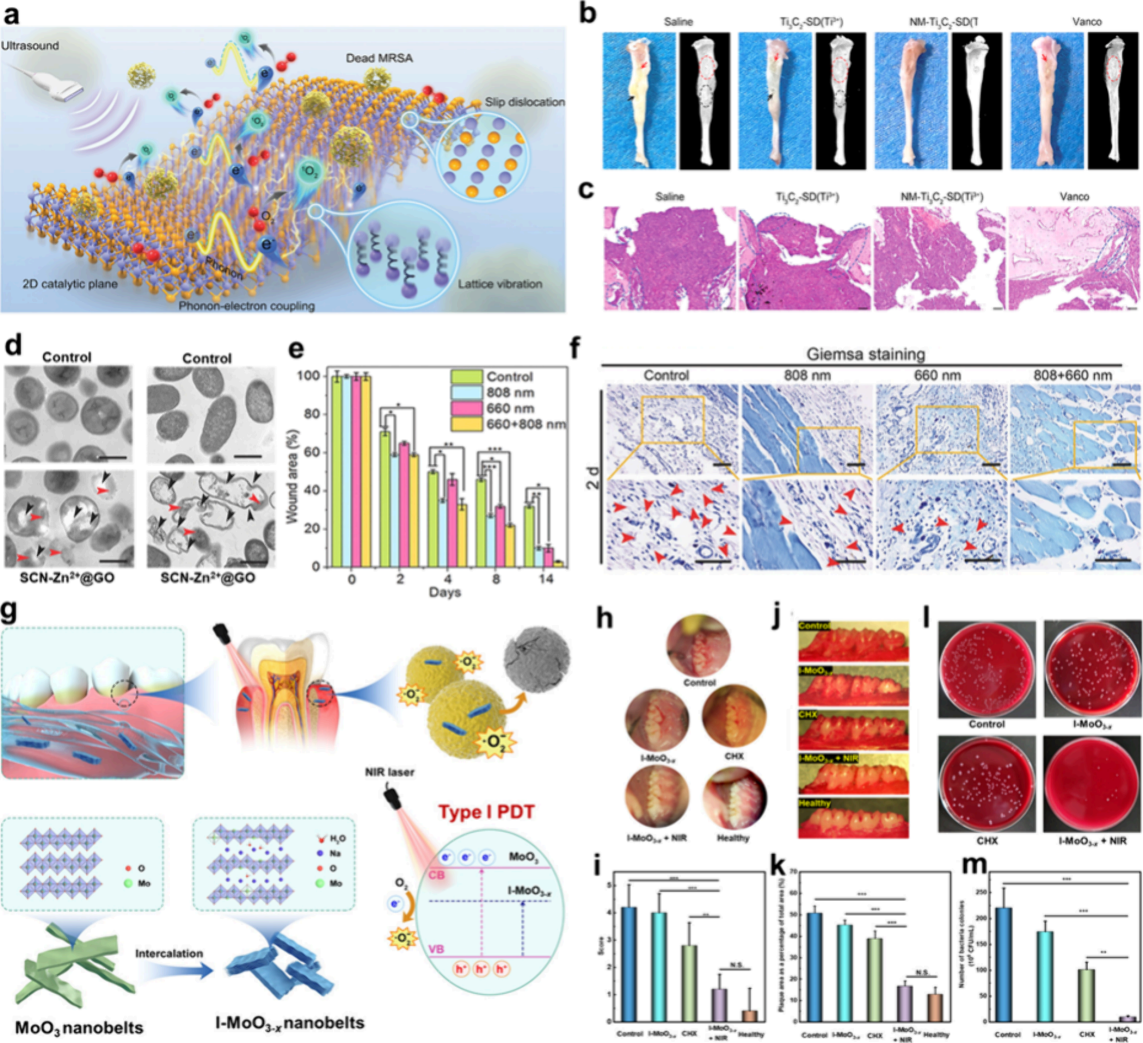
(a) Schematic diagram of Ti_3_C_2_[Ti_3_C_2_–SD­(Ti^3+^)] nanosheets-mediated ROS generation. (b) Macroscopic images of infected tibia specimens and (c) H&E staining images of infected bony tissues of mice after 14 days of various treatments. Reproduced with permission from ref 208. Copyright 2023 John Wiley and Sons. (d) TEM morphology of *S. aureus* and *E. coli* after different treatments. (e) Mean rate of wound healing based on the original wounded area of rats. (f) Immunohistochemical images of Giemsa-stained skin tissues on rat wounds. Reproduced with permission from ref 268. Copyright 2018 John Wiley and Sons. (g) Schematic illustration of intercalated MoO_3*–x*_ nanobelts for periodontitis treatment. (h) Photographs of the gingival bleeding of the oral cavity after various treatments and (i) corresponding quantitative results. (j) Photographs of the basic fuchsin-stained plaques and (k) corresponding quantitative results. (l) Digital photos of oral cavity bacteria reculture after various treatments and (m) corresponding quantitative results. Reproduced with permission from ref 61. Copyright 2023 John Wiley and Sons.

In addition, heteroatom doping is also useful for engineering layered nanomaterials and endowing them with antibacterial effects. Wang et al. prepared defect-rich N-doped WS_2_ (N-WS_2_) and N-doped MoS_2_ (N-MoS_2_) nanosheets by partly replacing sulfur atoms with N atoms.^487^ N-WS_2_ and N-MoS_2_ nanosheets exhibited superior peroxidase-like catalytic activities than pristine WS_2_ and MoS_2_ nanosheets, effectively killing *E. coli* (sterilization rates of 85.28% and 94.8%) and *B. subtilis* (sterilization rates of 80.96% and 86.04%) by generating toxic ·OH and promoting bacteria-infected wound healing. Similarly, Chen et al. proposed N- and B-codoped graphene oxide nanozyme (N-B-GO) for bactericidal application.^488^ The doping of N and B atoms significantly enhanced the peroxidase-mimetic activity of GO with high stability, acceptable recyclability and excellent reproducibility. Consequently, N-B-GO nanozyme exerted enhanced bactericidal ability against *S. aureus* and *E. coli* by virtue of ·OH generation mediated by peroxidase-mimetic activity. Li et al. also reported Zn^2+^-doped g-C_3_N_4_ modified with GO (SCN-Zn^2+^@GO) for antibacteria (Figure 20d–f).^268^ Due to Zn^2+^ doping and GO modification, SCN-Zn^2+^@GO possessed enhanced antibacterial efficacy with an antibacterial ratio over 99% owing to the synergistic effects of photodynamic and photothermal treatments under 660 and 808 nm laser irradiation.

Additionally, Li et al. activated the ·O_2_^–^ generation activity of MoO_3_ nanobelts via a Na^+^/H_2_O cointercalation strategy as a Type I PS for periodontitis treatment (Figure 20g).^61^ The intercalation engineering of MoO_3_ nanobelts induced multiple structural changes, including creating rich OVs, partially reducing the Mo^6+^ to Mo^5+^, enlarging its interlayer spacing, shortening its length, and yielding a color change from white to dark blue with enhanced NIR absorption. Therefore, the intercalated MoO_3*–x*_ nanobelts exhibited much higher ·O_2_^–^ generation activity than the pristine MoO_3_ nanobelts under 808 nm laser irradiation, exerting spectral antibacterial activity against *Saccharomyces aureus* and *E. coli* and yielding satisfactory therapeutic effect on periodontitis (Figure 20h–m).

In general, layered nanomaterials engineered by structural engineering strategies have demonstrated remarkable efficacy in antimicrobial applications. Nevertheless, current research predominantly focuses on sterilization mechanisms mediated by photothermal effects and enzymatic catalytic activities of structurally engineered layered nanomaterials, representing a limited spectrum of antimicrobial approaches. This underscores the critical need to develop a broader range of layered nanomaterials based on structural engineering strategies capable of diverse antimicrobial modalities, including but not limited to photodynamic inactivation, ion-interference therapy, physical disruption mechanisms, biofilm penetration and disruption, immunomodulatory effects. Such diversification is essential to address complex antimicrobial challenges across various clinical and environmental scenarios.

Furthermore, significant barriers remain in two critical areas: Synthesis optimization: current fabrication processes of engineering layered nanomaterials often lack reproducibility and scalability. Biosafety evaluation: comprehensive in vivo assessments of long-term biocompatibility, biodegradation pathways, and potential immunogenicity of engineering layered nanomaterials remain insufficient. Addressing these challenges requires: cross-disciplinary collaboration between materials scientists, microbiologists and clinical researchers; the development of standardized evaluation protocols for antimicrobial efficacy and safety; industry-academia partnerships to bridge the gap between laboratory-scale innovation and commercial-scale production. Only through such concerted efforts can the full potential of engineered layered nanomaterials as next-generation antimicrobial agents be realized in practical applications.

## 7 Summary and Outlook

In this Review, we have systematically summarized the advances in layered nanomaterials explored for diverse biomedical applications through structural engineering, including graphene and its derivatives, LDHs, TMDs, layered metal oxides, g-C_3_N_4_, and MXenes, and comprehensively discussed their composition, structural characteristics, and functional properties. More importantly, we provided a summary of the structural engineering strategies of layered nanomaterials, including crystal phase engineering, defect engineering, heteroatom doping, interlayer engineering, and crystalline-to-amorphous phase engineering, in which the detailed preparation methods and underlying mechanisms are introduced. Of particular interest is that these engineering strategies can enable precise regulation of varying structural features of layered nanomaterials, such as crystal phase, defect density, doping configurations, crystallinity, thickness, lateral dimensions, and surface properties, which are critical for diverse biomedical applications. Advanced characterization techniques, such as XRD, TEM/STEM, XPS, ESR, AFM, XAFS, Raman spectroscopy, and NMR spectroscopy, have been instrumental in clearly visualizing the structural changes induced by structural engineering on layered nanomaterials at the atomic level, facilitating the establishment of critical structure–property relationships. Furthermore, recent progress regarding the structural engineering of layered nanomaterials for diverse biomedical applications, e.g., drug delivery, bioimaging, cancer therapy (PTT, PDT, SDT, catalytic therapy, CDT, immunotherapy), theranostics, biosensing, and antibacteria, are also comprehensively illustrated.

Despite these advancements, the extensive exploration in the structural engineering of layered nanomaterials also poses critical interdisciplinary challenges and bottlenecks:

(1) Stability-performance trade-offs: From the perspective of property modification, structural engineering strategies can enhance the performance of layered nanomaterials in given applications, while they may also compromise the structural stability and activity. For instance, crystal phase engineering enables the design and preparation of heterophase nanostructures and their related layered hybrid materials, where the synergistic effects between different phases and/or varying components can result in enhanced performance for specific applications. How to achieve long-range ordered arrangement of different phases in heterophase layered nanomaterials to ensure stability and activity while leveraging synergistic effects is an intriguing problem. Manufacturing defects via defect engineering, heteroatom doping, or crystalline-to-amorphous phase engineering can create more active sites to achieve better performance, whereas it also causes the production of impurities or even damages the structure of layered nanomaterials. Interlayer engineering can reduce the diffusion barrier of ions within the host, but it may also lower the overall structural stability of layered nanomaterials and destabilize the layered framework. These issues necessitate controlling defect density and selective intercalation without sacrificing the structural stability of layered nanomaterials. In such cases, a trade-off is usually expected between stability and performance, and precise optimization is necessary to find the sweet spot.
(2) Mechanism understanding limitations: From the perspective of mechanism understanding, these reported structural engineering strategies usually involve complex multiparameter design rules with insufficient mechanism insights. For instance, both acid and alkali etching can construct defect-rich or amorphous LDHs materials. It is not yet clear how distinct design rules or their combination affect the structure and properties of layered nanomaterials. The core of this deficiency is the lack of explanation for the relevant mechanisms. To bridge this knowledge gap, a deeper understanding of these mechanisms through advanced theoretical modeling and in situ characterization techniques will be of great significance, providing valuable information for the rational design of layered nanomaterials with new structures and functions. It is foreseeable that the future development of characterization methods, including various in situ observation techniques, will enable a more in-depth analysis of the structural changes in layered nanomaterials. Furthermore, with the help of advanced high-performance computing systems and even emerging artificial intelligence (e.g., machine learning),^489^ it is possible to screen the most appropriate structural engineering strategies to engineer layered nanomaterials, predict new structures that have not yet been experimentally observed, and decode structure–property correlations.
(3) Scalability challenges: From a practical application perspective, the current quantity, yield, quality and structural consistency of layered nanomaterials in mass production still fall significantly short of the criteria necessary for commercial viability. A significant disparity persists between laboratory-scale structurally engineered layered nanomaterials and their practical large-scale applications. Key challenges arise from the inherent chemical or physical instability of these engineered layered nanomaterials, as well as difficulties in scaling up production. Layered nanomaterials usually suffer from inherent instability during processing and are prone to environment-induced degradation in real-world applications, particularly when their engineered structures are considered. A controlled preparation of layered nanomaterials with desired structures remains a significant challenge for most existing structural engineering strategies. Some structural engineering strategies, especially those involving atomic-level structural modulation, require specialized synthesis techniques or conditions and lack the scalability for practical applications. Therefore, the engineering of layered nanomaterials with desired structure, performance and functionality in a highly controllable manner remains one of the critical hurdles in this field. Great efforts should be focused on exploring simple, robust, and scalable structural engineering methods while preserving structural precision to produce layered nanomaterials with high stability on a large scale. Additionally, constraints arising from the limited scope of public knowledge databases, pose significant challenges in developing universal theories for the rational design and optimization of synthesis pathways for structurally engineered layered nanomaterials. This limitation also hampers the scalability of these materials. To overcome these obstacles, a forward-looking strategy to accelerate progress in this field involves: screening and identifying existing literature-reported process routes with scale-up potential, with focused optimization of critical factors including: scalability (e.g., mildness of reaction conditions, raw material accessibility, process compatibility), supply demand gaps (e.g., clinical requirements), and safety (e.g., metabolic pathways, cytotoxicity thresholds). By integrating literature data with experimental validation, candidate pathways will be subjected to parametrized prioritization. The results of this screening will be organized into a structured database, which will provide foundational data to support the future development of comprehensive universal models linking structure, property, and process.

In future work, existing structural engineering strategies will continue to serve as powerful tools for optimizing the structure and intrinsic properties of layered nanomaterials and strengthening their performance. Despite the challenges, the field of structural engineering of layered nanomaterials for biomedical applications is rapidly evolving, and several promising directions can be identified to further advance this area of research. These future directions not only aim to expand the scope of materials and applications, but also to deepen our understanding of the structure–property relationships that underpin their functionality.

(1) Expansion to other 2D nanomaterials: While significant progress has been made in structural engineering of layered nanomaterials like graphene, LDHs, TMDs, layered metal oxides, g-C_3_N_4_, and MXenes, there is considerable potential in engineering other 2D nanomaterials (such as black phosphorus, telluride, layered oxides, oxyhalides, covalent organic frameworks, metal–organic frameworks, and perovskites, etc.) through existing structural engineering strategies for diverse biomedical applications. From a microscopic perspective, structural engineering strategies can be used to guide the synthesis and stabilization of atomic pairs, single atoms, or other forms of atomic arrangements. From a macroscopic perspective, structural engineering can also be employed to construct nanomaterials from building blocks other than ions and atoms, e.g., superlattice materials or nanoparticles. Expanding the concept of structural engineering to other types of nanomaterials will open a pathway for discovering a variety of new functional biomedical nanomaterials. Future research should focus on adapting existing structural engineering methods to these emerging 2D nanomaterials to unlock their full potential in biomedicine.
(2) Development of novel structural engineering strategies: Inspired by the field of catalysis, new structural engineering strategies could be developed to enhance the performance of layered nanomaterials in biomedical applications. For example, structural engineering strategies involving electrochemistry, optical radiation, or the use of magnetic field and electric field could be explored to regulate the structure of layered nanomaterials.^103^ Techniques such as in situ growth of single atoms within the interlayer spaces,^490^ creation of cation vacancies,^491^ precise control of layer stacking,^492^ and construction of porous/holey architectures and 2D heterostructures,^42^ may lead to layered nanomaterials with improved drug loading capacities, enhanced targeting efficiency, and better therapeutic outcomes. In addition, atomic-level structural engineering strategies can be combined with other engineering strategies, such as composition, dimensionality or architecture engineering, to achieve a higher level of control over various layered nanomaterials. These strategies could also enable the fine-tuning of electronic and optical properties, which are crucial for applications like photothermal therapy, photodynamic therapy and bioimaging.
(3) Exploration of new biomedical applications: Beyond the well-established applications in drug delivery, bioimaging, cancer therapy, theranostics, biosensing, and antibacteria, there is a growing interest in exploring new biomedical applications for layered nanomaterials enabled by structural engineering. For example, endowing layered nanomaterials with anti-inflammatory properties through structural engineering strategies could be leveraged to develop novel treatments for chronic inflammatory diseases. Additionally, enhancing the mechanical properties and biocompatibility of layered nanomaterials through structural engineering strategies make them suitable candidates for bone repair and tissue engineering. Future research should aim to systematically investigate these new applications and develop tailored nanomaterials through structural engineering to meet the specific requirements of each application.
(4) Adoption of advanced characterization techniques: To fully grasp the relationship between structural modifications and material functions, it is essential to employ advanced characterization techniques. In situ characterization methods, such as in situ XRD, in situ TEM, in situ ESR, in situ Raman, in situ XAFS,^493^ in situ irradiated XPS, in situ surface photovoltage microscopy (SPVM),^494^ scanning tunneling spectroscopy (STS), and scanning tunneling microscopy (STM),^66^ can provide real-time insights into the structural changes, charges transfer and distribution occurring during material synthesis and application. These techniques will enable researchers to establish clear structure–property relationships, which are crucial for the rational design of next-generation layered nanomaterials for biomedical applications. Furthermore, high-performance computing systems and artificial intelligence can be utilized to predict unexplored structural configurations and optimize synthesis parameters.

By pursuing these future directions, the field of engineered layered nanomaterials for biomedical applications can continue to grow and provide innovative solutions to some of the most pressing challenges in medicine and healthcare. Ultimately, structural engineering will remain pivotal in advancing functional layered nanomaterials for biomedicine, provided that fundamental studies address current limitations in stability control, mechanism understanding, and scalable fabrication.
